# Alternative splice variants of rhomboid proteins: Comparative analysis of database entries for select model organisms and validation of functional potential

**DOI:** 10.12688/f1000research.13383.2

**Published:** 2018-05-31

**Authors:** Joshua Powles, Kenton Ko

**Affiliations:** 1 Department of Biology, Queen's University, Kingston, Ontario, K7L 3N6, Canada

**Keywords:** Rhomboid proteins, alternative splicing, activity, transgenic expression, recombinant rhomboid proteins, protein structure, model organisms, databases

## Abstract

**Background:** Rhomboid serine proteases are present across many species and are often encoded in each species by more than one predicted gene. Based on protein sequence comparisons, rhomboids can be differentiated into groups - secretases, presenilin-like associated rhomboid-like (PARL) proteases, iRhoms, and “inactive” rhomboid proteins. Although these rhomboid groups are distinct, the different types can operate simultaneously. Studies in *Arabidopsis* showed that the number of rhomboid proteins working simultaneously can be further diversified by alternative splicing. This phenomenon was confirmed for the *Arabidopsis* plastid rhomboid proteins At1g25290 and At1g74130. Although alternative splicing was determined to be a significant mechanism for diversifying these two *Arabidopsis* plastid rhomboids, there has yet to be an assessment as to whether this mechanism extends to other rhomboids and to other species.

**Methods:** We thus conducted a comparative analysis of select databases to determine if the alternative splicing mechanism observed for the two *Arabidopsis* plastid rhomboids was utilized in other species to expand the repertoire of rhomboid proteins. To help verify the *in silico* observations, select splice variants from different groups were tested for activity using transgenic- and additive-based assays. These assays aimed to uncover evidence that the selected splice variants display capacities to influence processes like antimicrobial sensitivity.

**Results:** A comparison of database entries of six widely used eukaryotic experimental models  (human, mouse, *Arabidopsis*, *Drosophila*, nematode, and yeast) revealed robust usage of alternative splicing to diversify rhomboid protein structure across the various motifs or regions, especially in human, mouse and *Arabidopsis*. Subsequent validation studies uncover evidence that the splice variants selected for testing displayed functionality in the different activity assays.

**Conclusions: **The combined results support the hypothesis that alternative splicing is likely used to diversify and expand rhomboid protein functionality, and this potentially occurred across the various motifs or regions of the protein.

## Introduction

Rhomboid proteins are found widely in all types of organisms, spanning bacteria, archaea, eukaryotes. In higher-order organisms, rhomboid proteins are often encoded by a large group of genes ^1– 4^, for example, upwards of twenty-two database entries reported for *Arabidopsis* and thirteen for humans (assessed as of May 2017). Phylogenetic studies, such as that conducted by Lemberg and Freeman (2007), suggest that rhomboid genes can be divided into two subgroups encoding proteolytically active secretases- and Presenilin Associated Rhomboid Like (PARL)-type rhomboid forms, and two subgroups of catalytically inactive forms (“non-proteases”) such as iRhoms, Derlins, and other distantly related forms ^1, 5– 8^.

The ‘active’ category of structurally diverse rhomboid proteases are often found to occupy regulatory roles of different cellular activities, typically by interacting with and cleaving specific membrane-residing substrates. Examples being the epidermal growth factor signaling pathway in fruit flies ^9^, the quorum-sensing mechanism of the bacterium *Providencia stuartii* ^10, 11^, yeast mitochondrial membrane remodelling ^12^, and the health status of mitochondria in human cell lines ^13^.

The ‘inactive’ subcategories consist of structurally diverse rhomboid proteins as well and are believed to lack the needed catalytic residues used by the active rhomboid proteases ^1^. Despite the absence of the catalytic residues, some of the inactive rhomboid proteins are found to be functionally significant without being active proteases ^9– 12, 14^. In some of the reported cases, interaction alone with an inactive rhomboid, without proteolysis, is sufficient to cause effects, such as growth signaling in cancer cells (human iRhom1), dislocation of proteins in the endoplasmic reticulum protein (ER) (mammalian Derlins), ER protein quality control *(Drosophila* iRhom Rhomboid-5), development, and organelle biogenesis ( *Arabidopsis* At1g74130) ^5– 8, 14– 20^. Even the active human rhomboid protease, RHBDL4, a promoter of ER-associated degradation of membrane proteins, physically interacts with ubiquitin in order to proceed with its protease activities ^21^.

In addition to the plethora of already functionally diverse active and inactive rhomboid proteases, alternative splicing appears to generate even more structural variants with modified functionalities ^1^. For example, one case was confirmed for the human RHBDD2 gene ^22^. Levels of the two alternatively spliced RHBDD2 mRNAs were elevated in breast cancer cell lines ^22^, suggesting a link between cancer cell activity, and the presence of splice variants with a sizable difference between the two variant protein structures. In *Arabidopsis,* the active plastid rhomboid At1g25290 was confirmed to exist as two functionally significant splice variants that differ by the presence of a potential cyclin-binding motif, a motif known to be involved in cell cycling ^23^. One of the inactive plastid rhomboids predicted for *Arabidopsis*, At1g74130 ^1^, also exists as three splice variants, with distinct functionalities and different levels of interactions with the Tic40 substrate ^24^. Alternative splicing resulted in a substantial impact to the carboxyl transmembrane segment of At1g74130, changing from a seven predicted to six transmembrane structure. Functionality differences of the three At1g74130 splice variant proteins were apparent upon testing at the whole cell level in bacteria and yeast. Despite being plant-derived, the At1g74130 splice variants exhibited physiological interactions with the mitochondrial rhomboid protease Rbd1 in yeast cells, and modulated differently the cleavage ratio of the resident mitochondrial protein Mgm1, a ratio that governs mitochondria remodeling and respiratory status ^24^.

To date, the phenomenon of diversifying rhomboid protein functionality through alternative splicing has been documented for two *Arabidopsis* plastid rhomboids. It has not yet been assessed as to whether this phenomenon is limited, or represents a mechanism for expanding the number of functional rhomboid forms in a wide range of rhomboid systems and organisms. Therefore, using the findings reported for At1g25290 and At1g74130 as guidance, we conducted a comparative analysis of entries available in the genetic databases of six widely used eukaryotic experimental models to assess how splice variants may be reflective of ways to diversify functionality, from limited amino acid changes to substantive structural deletions. A limited selection of alternatively spliced variants were then analyzed to document evidence of potential activity as proteins using different types of assays.

## Methods

### Comparison of variant sequences in current databases and categorization scheme

The sequence entries compared were assembled from current versions of the publicly-accessible databases (last sampled as recent as May 31, 2017). We used the NCBI database ( RefSeq, RRID:SCR_003496) as our primary source for the six widely-used model organisms ( *Homo sapiens* (human), *Mus musculus* (mouse), *Arabidopsis thaliana (Arabidopsis*), *Drosophila melanogaster* (fruit fly), *Caenorhabditis elegans* (nematode) and *Saccharomyces cerevisiae* (Baker’s yeast). We also cross-checked with other databases such as the Mouse Genome Informatics resource (Mouse Genome Informatics, RRID:SCR_006460), TAIR for *Arabidopsis* (TAIR, RRID:SCR_004618), FlyBase (FlyBase, RRID:SCR_006549), WormBase (WormBase, RRID:SCR_003098), and the Saccharomyces Genome Database (SGD, RRID:SCR_004694). All entries used and assessed are compiled in Table S1 along with their relevant details. Note that some of the entries listed in supplementary table are for the predicted forms, despite being assessed by comparison to RNA-seq data, for example. Table S2 provides a listing of formal and alternate names used for each rhomboid, along with Gene ID numbers and related references. Database entries were compiled and assessed for alternative splicing before use by comparing entries using publicly available bioinformatic tools for alignment work (the suite of BLAST (RRID:SCR_007190), Clustal (RRID:SCR_001591), and LALIGN tools). Entries were retrieved from databases for cDNA/EST sequences, RNA-seq, genomic sequences, and proteins. This assessment stage was not designed to correct predictions, address gaps, or add predictions, but to assess the existing entries and the overall capacity of alternative splicing to diversify rhomboid protein functionality. This study’s objective was thus focused on surveying the functional potential of the available entries with the understanding that some will require further validation work. The categorization of alternative splicing events/splice variants was based on the potential impact of changes on motifs along the protein, from amino to carboxyl terminus. The use of six eukaryotic rhomboid gene systems was to help determine common trends. The protein models and motifs used for categorization were adapted from the models reported by Lemberg and Freeman ^1^. In many cases, alternative splicing events tend to influence one motif, but there were a number of cases where the impact may affect one or more motifs, depending on the type and location of the splicing event. For the purpose of comparing the impacts of alternative splicing, all variant sequences were assumed to result in the generation of functional proteins in some capacity, including extensively truncated products. For this study, sequences and splicing events are categorized at the motif-specific or region-specific level and may thus appear in more than one category. For instance, afrequent occurrence is an alternative splicing event which occurs in a particular motif that would likely impact the adjacent linker region. The various splice variants are listed in Tables S3 to S10 by category. Information concerning the impact of the splicing events is also included in these Supplementary Tables. Overviews are provided in the Results section. Specific examples are highlighted when applicable.

### Alignments and analyses of changes to protein structure

Linear protein alignments were carried out first using the publicly available bioinformatics tools listed in the above section, such as Clustal Omega (RRID:SCR_001591). Select sets of the linear alignments are included in the Supplementary Material. Structural predictions were facilitated by comparing alignments with predicted structures constructed and reported by Lemberg and Freeman ^1^. Three-dimensional predictions of splice variant protein structures were created and compared by utilizing Phyre Version 2 services (RRID:SCR 010270) ^25^ and visualized using PyMol 1.1 (RRID:SCR 000305). Comparisons were conducted using the bacterial rhomboid GlpG model ^26^. These 3D predictions were carried out solely for the purpose of investigating and speculating the capacity for structural impact of the different splicing events. All of these 3D predictions are provided and reported in the Supplementary Material for consideration only and do not represent established, solved structure data.

### Production of select splice rhomboid protein variants and immunoblot assessments

Select proteins were synthesized in *Escherichia coli* JM109 (DE3) and facilitated by the T7 promoter of pET20b (EMD4 Biosciences, Rockland, MA, USA). Histidine tags were joined in-frame to the carboxyl-terminus. Cells were grown at 16°C in ampicillin-containing Terrific Broth (Bioshops Inc., Burlington, Canada) (25 µg/ml ampicillin). Recombinant histidine-tagged proteins were purified using nickel – nitriloacetic acid affinity chromatography (Qiagen, Toronto, Canada). The protein expression and chromatography procedures followed and the composition of the buffers used were as reported and cited previously ^24^. Briefly, bacterial cells were harvested by centrifugation, resuspended in a small volume of extraction buffer, and disrupted using a French Pressure Cell Press (2 cycles of 15,000 psi using a medium cell). Elution was carried out in one step using a small volume of the cited pH 7.5 elution buffer and 400 mM imidazole. Proteins were quantified using the Bradford assay system (Biorad, Hercules, CA, USA) and normalized before use. Proteins were typically stored as concentrated stocks at -80°C and used later for the various assays. Freeze-thawing of recombinant rhomboid proteins did not have an observable impact on activity.

Protein samples, typically 0.5 μg per lane, were analyzed when needed using standard one-dimensional (1D) 12% (m/v) sodium dodecyl sulfate (SDS) – polyacrylamide gels. Electrophoretic and immunoblotting protocols were performed according to Laemmli ^27^ and Towbin *et al*. ^28^. Immunoreactive bands were analyzed using scans, quantitated by densitometry (Scion Image 4.0.3.2, Scion Corporation, USA), normalized, and compared relative to internal references when applicable. All immunoblots were repeated at least three times within each experiment and with biological replicates. Representative results are then shown in the figures. When needed, quantitations were conducted using nonsaturated scans of the images presented in the figures. Various recombinant protein preparations were checked by immunoblotting using rabbit polyclonal anti-rhomboid protein antibodies that were established by our lab and validated previously as reported in Powles *et al*. ^29^. For samples derived from transgenic yeast cell assays, rabbit polyclonal anti-yeast mtHsp70 antibodies (Antibodies-Online Cat# ABIN488515, RRID:AB_11209968) were used for normalization as reported previously in Powles *et al.* ^29^. The normalized ratio strategy was designed to allow semi-quantitative assessment of profile changes independent of experiment, gel origin, and exposures. Statistical analyses or details are noted where applicable.

### Assays for testing biological activity of select splice rhomboid protein variants

*Activity assays using transgenic yeast* – Protein expression in *S. cerevisiae* (Baker’s yeast) was facilitated by the yeast – *E. coli* shuttle vector pACT2 (BD Biosciences-Clontech, San Jose, CA, USA). In all cases, expression of the inserted cDNAs was driven by the cloned yeast Rbd1 promoter. The introduction of plasmids was carried out using standard yeast transformation techniques. The host yeast strain C6000 used was acquired from EUROSCARF (EUROpean Saccharomyces Cerevisiae ARchive for Functional Analysis, RRID:SCR_003093) (Frankfurt, Germany). Cells were grown in glucose-supplemented media (at 30°C in standard glucose-supplemented yeast Complete Medium without leucine where applicable (20 g/l glucose, 6.8 g/l yeast nitrogen base without amino acids, (Sigma-Aldrich, Oakville, ON, Canada), 1.6 g/l drop out mix (Sigma-Aldrich, Oakville, ON, Canada), and 20 g/l agar when used). All strains were prepared as populations (as opposed to from single colonies) and stored at -80°C as glycerol stocks.

A disk diffusion method was employed to test different yeast strains for changes to nystatin sensitivity as a result of the indicated splice variant proteins being expressed. Yeast cells were suspended in top agar (1% w/v) media at 5 × 10 ^7^ cells/mL and poured onto agar plates of the same medium used above (2% w/v). Sterilized filter paper disks (38.5 mm ^2^) infused with nystatin, were evenly positioned on the top agar, at a typical concentration of 10 disks per 90 mm plate. Each disk was infused with 10 µL of a 2.4% (v/v) nystatin solution, diluted with the appropriate yeast culturing medium. Plates were incubated overnight at 30°C. The zone of growth inhibition around each disk was then measured, and the corresponding area calculated. Multiple independent experiments were conducted to confirm the functional potential of the various splice variants.

*Activity assays using yeast and externally-added proteins* - Non-transformed cells (without plasmids) were also assessed with exogenously added splice variant proteins. Since amphotericin B (AmB) has the ability to introduce transmembrane channels for protein delivery, cells were incubated for one hour with 10 µg (at 1 µg/mL, made in 23X diluted elution buffer (diluted with growth media) of the indicated recombinant variant protein along with a 1% (v/v) AmB solution (this level has no detrimental effect on yeast cell survival). Controls were carried out prior to the experimental assays with various diluted elution buffer levels to assess the base level effects without recombinant rhomboid proteins. When shown, the controls or mock assays were conducted with the above 23X diluted elution buffer. Minimal volumes of cells, typically at 5,000 cells/mL, were used. Various levels of nystatin were then added and incubated for the last 15 minutes of the 1 hour treatment period. After the 1 hour incubation-treatment, 500 cells were plated (per 90 mm petri plate) and grown for 48 hours at 30°C to assess sensitivity to nystatin as a test for biological activity and for any differences between splice variants. Results from independent replicates were analyzed statistically (t-test) between control (or mock) and experimental assays and noted with details where applicable.

*Activity assays using transgenic bacteria – E. coli* JM109 (DE3) cells harboring various pET20b-based splice variant constructs were plated on LB agar containing varying concentrations of ampicillin as indicated. The assessment of ampicillin sensitivity was not dependent on induction of expression, but instead relied upon the inherent “leaky” expression. Plating was normalized with equal colony numbers. Results from independent experiments were analyzed statistically (t-test) between control (or mock) and experimental assays, and noted with details where applicable. Changes in ampicillin sensitivity were further examined using whole cell extracts and immunoblotting to assess β-lactamase expression, secretion, and processing of the precursor β-lactamase form. Whole cell extracts were prepared from cell pellets harvested by centrifugation of liquid cultures and boiled in standard protein gel loading dye.

*Activity assays using bacteria and externally added proteins* - Bacteria (HB101) were assessed in some cases with exogenously added splice variant proteins. Cells harboring pET20b were used as the ampicillin resistance model for testing biological activity and differences between splice variant proteins. Dimethyl sulfoxide (DMSO) was utilized to permit the delivery of proteins into cells ^30^ by an initial incubation of 30 minutes with 5% (v/v) DMSO, 10 μg of variant protein (normalized with 23X diluted elution buffer (diluted with LB broth)), and LB broth. The controls used here for bacterial cells were established and conducted in the same manner as described for the above yeast cell assays. After the 30 minute protein delivery period, each treatment was incubated with 1.25 or 1.5 mg/mL of ampicillin (and adjusted if needed) for an additional 45 minutes. Treatments were carried out at 37°C with shaking (100 rpm). Bacteria were normalized to 800 cells per treatment in a total volume of 200 μL, and plated in its entirety on LB plates and grown overnight at 37°C. Surviving colonies were counted and compared to mock treatments, which consisted of all components without proteins. Results from independent experiments were analyzed statistically (t-test) between control (or mock) and experimental assays and noted with details where applicable.

## Results

### Rationale and justification for this database study

We previously verified in separate studies a mechanism for diversifying rhomboid proteins and their functionality. This alternative splicing mechanism played diversifying roles for two different *Arabidopsis* plastid rhomboid genes - the active secretase type At1g25290 and the inactive PARL type At1g74130. Alternative splicing impacted different parts of the proteins with no apparent functional similarities to each other. The At1g25290 splice variants were focused on controlling the appearance of the cyclin-binding RVL motif in the protein’s middle segment, right after the third predicted transmembrane region ^23^. The data underlying the characterization of the At1g25290 splice variants (designated L and S) and the splicing events involved to generate the protein variants were reported previously ^23^. The composition of the resulting At1g25290 protein variants (L and S) used later in this study are also shown in the Supplementary Material. The splice variants created for At1g74130 resulted in different shortened proteins, each missing a key glutamine residue in the last carboxyl transmembrane region ^24^. Alternative splicing resulted in a substantial impact to the carboxyl transmembrane segment of At1g74130, changing from a seven predicted to six transmembrane structure ^24^. The data underlying the characterization of the At1g74130 splice variants (designated L, M, and S) and the splicing events involved to generate the protein variants were reported previously ^24^. The composition of the resulting At1g74130 protein variants (L, M, and S) used later in this study are also shown in the Supplementary Material. Both studies provided evidence that the resulting variant proteins display altered functionality ^23, 24^. These two sets of findings alone bring the total number of plastid rhomboid forms in *Arabidopsis* to at least seven, two for At1g25290, three for At1g74130 and at least one each for At1g74140 and At5g25752.

The outcomes discussed above prompted us to look at rhomboid genes of other eukaryoticspecies for evidence of similar diversification mechanisms. We thus compared and analyzed the RNA sequence databases of six eukaryotic organisms used widely as experimental models - human, mouse, *Arabidopsis*, *Drosophila*, *C. elegans*, and *S. cerevisiae*. Even though the databases continue to evolve, the observations disclosed here should continue to be applicable.

### Is alternative splicing present in different rhomboid gene systems?

**Figure 1.**
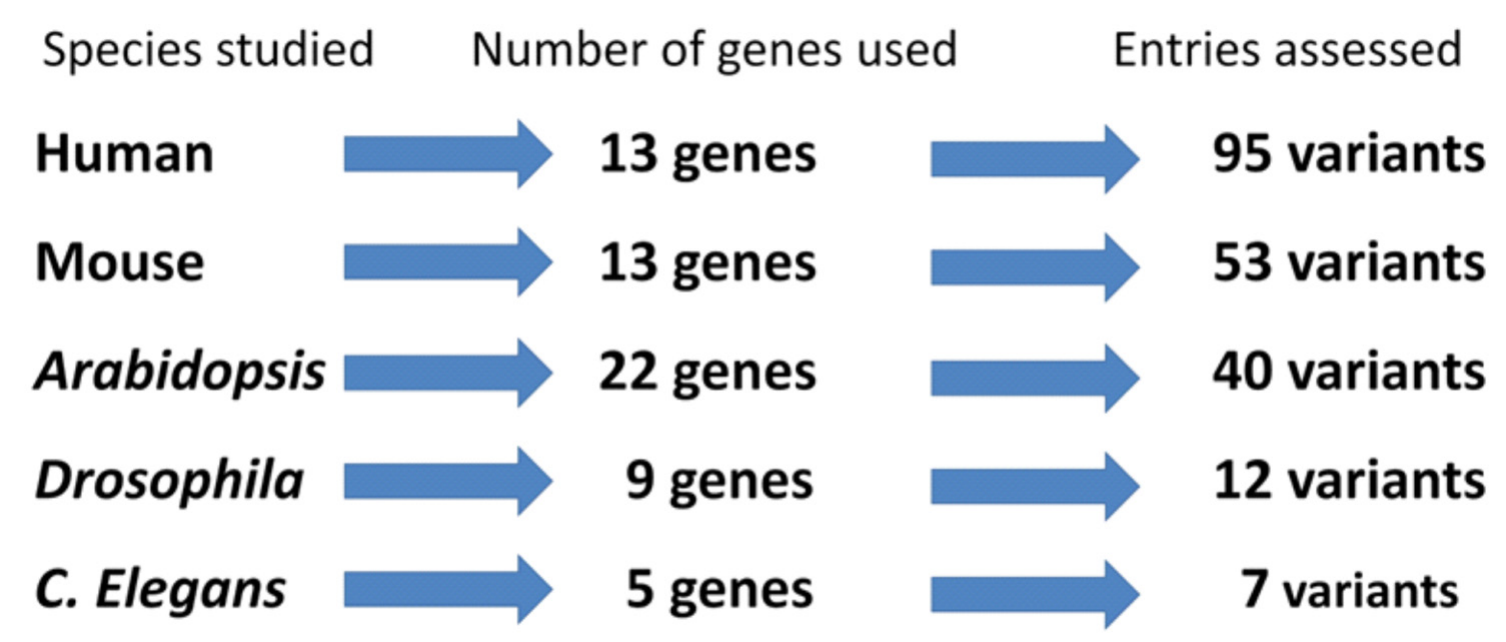
Visual summary of available sequence entries retrieved and studied for the selected eukaryotic organisms. Each of the selected eukaryotic organism is indicated along with the total number of rhomboid and rhomboid-like genes found in the databases and the number of accompanying variant sequences obtained and studied in this report. Entry details are summarized in Table S1. All entry types were analyzed.

The first aspect to establish was the presence of alternative splicing and its extent within a selected eukaryotic species, and across the different selected species. Using the current versions of the RNA sequence databases, we compiled and assessed all possible RNA sequence entries that were derived from alternative splicing. The human, mouse, and *Arabidopsis* assessments revealed many potential alternative splicing events for different rhomboid genes of these species. There were 95 entries in human, 53 in mouse and 40 in *Arabidopsis* ( Figure 1). In contrast, similar analyses of *Drosophila*, *C. elegans* and *S. cerevisiae*, revealed minimal levels to no evidence of alternative splicing. We found one possibility each for *Drosophila* and *C. elegans* and none so far for *S. cerevisiae.* It is, however, possible that the outcomes observed for the latter three model species were due to the number of reported alternate RNA sequences at the time of assessment. This was the case in *Arabidopsis* where alternative splice variants for At1g25290 and At1g74130 were discovered and verified upon further analysis of transcript populations ^23, 24^.

**Figure 2.**
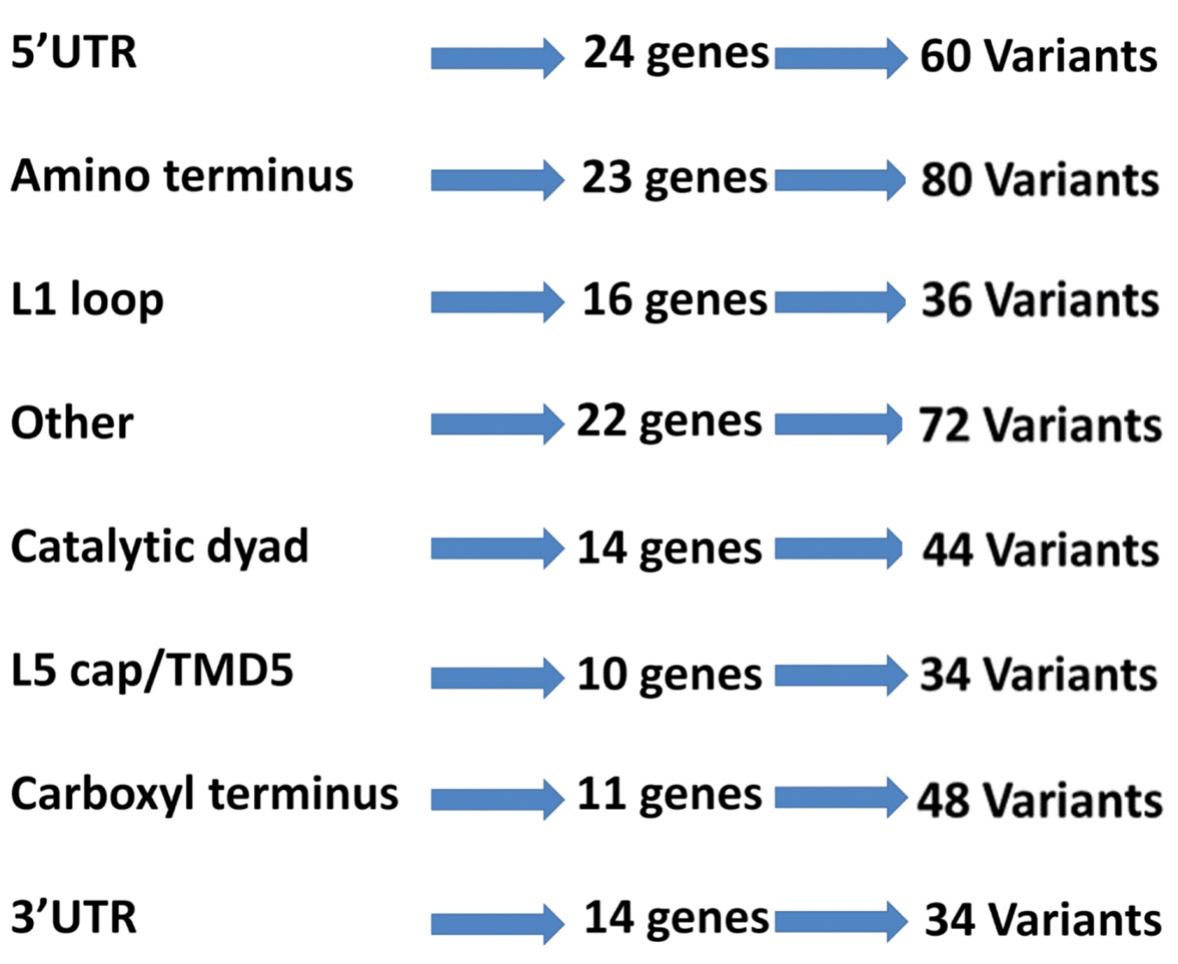
Schematic summary of splice variant types associated with different regions of a generalized rhomboid protein. Each region of the generalized rhomboid protein is indicated along with the total number of rhomboid and rhomboid-like genes that were accompanied by splice variants found in the databases. The genes considered belong only to the eukaryotic organisms selected for study here and are tallied together independent of species. The total number of accompanying alternative splice variants determined for each region is also provided. These variants are again tallied independent of species. Entry details are summarized in Table S1.

Of the six eukaryotic species analyzed, the human rhomboid system appears to exhibit the most alternative splice variants. All 13 of the rhomboid or like genes display multiple entries reflective of alternative splicing (see Figure 2 and Table S1). For instance, human PARL contains verified splice variants and additional predicted mRNA sequences or proteins. Similar situations were observed for human RHBDF2 (iRhom2), RHBDL1, RHBDD1, and RHBDD2.

The use of alternative splicing is also evident in the mouse rhomboid genes. The assessment revealed evidence of multiple alternative splice variants for mouse rhomboids ( Figure 1 and Table S1).

The relatively small genome of *Arabidopsis* appears to possess a high number of rhomboid and like genes. There are 22 entries and 10 are accompanied by 1 or 2 additional splice variant sequences ( Figure 1 and Table S1). The splice variants arising from At1g25290 and At1g74130 were discovered and verified in two other studies ^23, 24^. Based on the trends observed for human and mouse, it is likely that there are other splice variants in *Arabidopsis* awaiting discovery, especially for the other 12 gene entries currently without accompanying sequence variants in the database.

### What are the potential types of changes introduced by alternative splicing?

**Figure 3.**
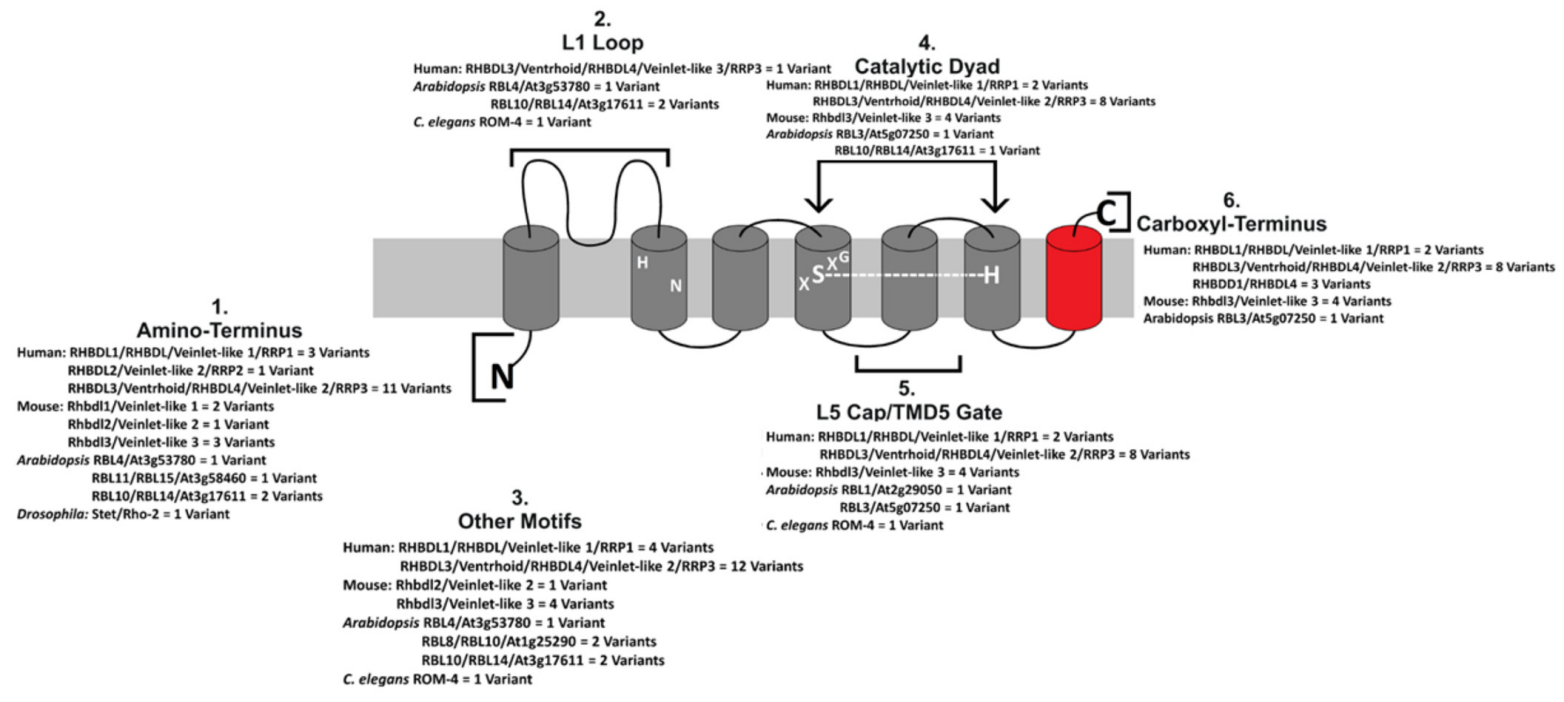
Locations of splice variants in the secretase-type rhomboids. Alternative splicing and resulting changes to the secretase-type rhomboid proteases (6+1 model). Amino acid sequences of the splice variant proteins were aligned and compared. The outcomes of these comparisons are compiled in the Supplementary Tables. The results are summarized in this figure. The regions of the protein potentially influenced by the changes are annotated. The protein structure is constructed using information from Lemberg and Freeman ^1^. Information concerning the names used in the figure is provided in Table S2.

Further analyses of the Figure 1 entries indicate that many of the occurrences are likely reflective of mechanisms for diversifying functionality ( Figure 2 and Table S3–Table S10). Structural changes were observed for both active rhomboid proteases (secretases and PARLs) and rhomboid-like proteins (inactive rhomboids and iRhoms) ( Figure 3– Figure 6, Table S4–Table S9). Potentially impactful changes were located in domains found across the entire protein structure ( Figure 2– Figure 6 and Table S3–Table S9). Changes were also observed within the 5’ UTRs that may affect translation and 3’ UTRs that may affect transcript properties ( Table S3 and Table S10). The changes impacting the protein can be subtle, affecting a few amino acid residues, to entire sections of the protein. In some instances, there were extensive deletions, insertions, truncations, or shortenings of the protein.

**Figure 4.**
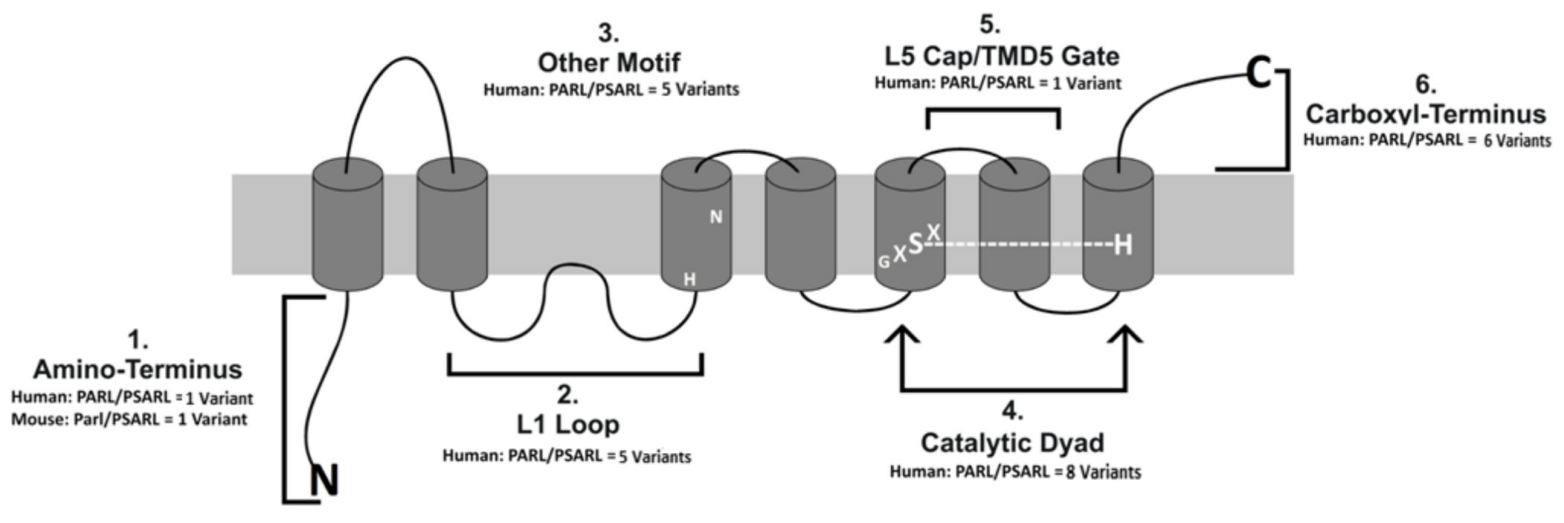
Locations of splice variants in the PARL-type rhomboids. Alternative splicing and resulting changes to the PARL-type rhomboid proteases. Amino acid sequences of the splice variant proteins were aligned and compared. The outcomes of these comparisons are compiled in the Supplementary Tables. The results are summarized in this figure. The regions of the protein potentially influenced by the changes are annotated. The protein structure is constructed using information from Lemberg and Freeman ^1^. Information concerning the names used in the figure is provided in Table S2.

**Figure 5.**
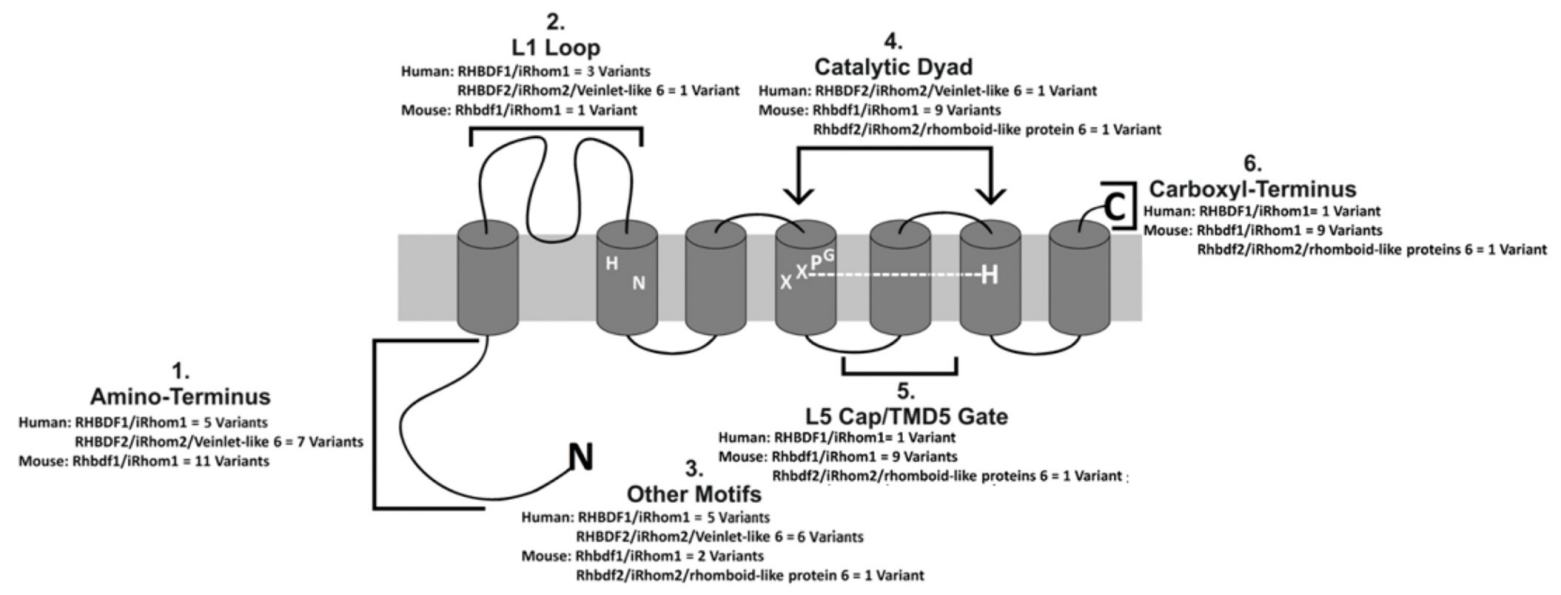
Locations of splice variants in the category of iRhom proteins. Alternative splicing and resulting changes to iRhom proteins. Amino acid sequences of the splice variant proteins were aligned and compared. The outcomes of these comparisons are compiled in the Supplementary Tables. The results are in this figure. The regions of the protein potentially influenced by the changes are annotated. The protein structure is constructed using information from Lemberg and Freeman ^1^. Information concerning the names used in the figure is provided in Table S2.

**Figure 6.**
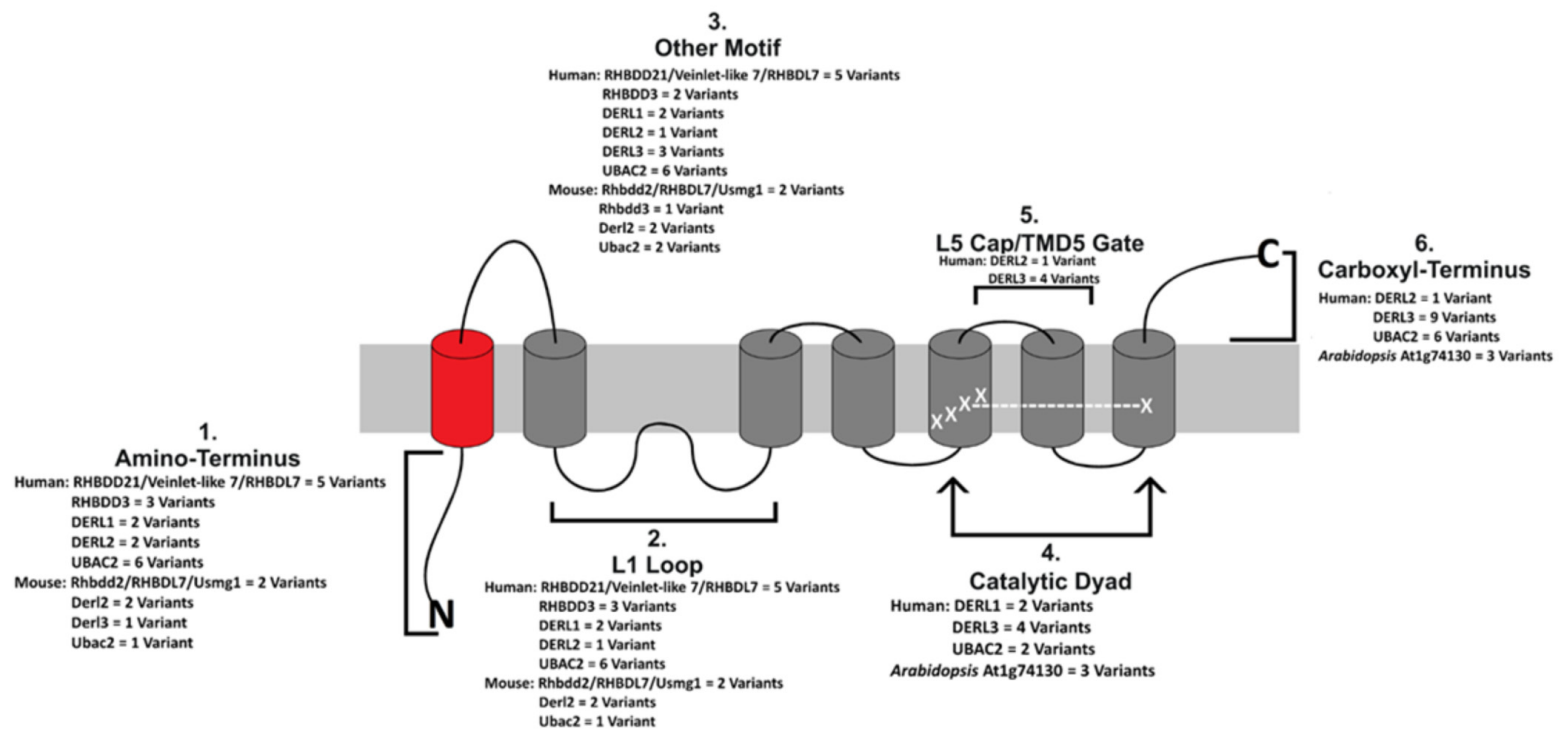
Locations of splice variants in the “inactive” category of rhomboid proteins. Alternative splicing and resulting changes to “inactive” rhomboid proteins (using the 1+6 model). Amino acid sequences of the splice variant proteins were aligned and compared. The outcomes of these comparisons are compiled in the Supplementary Tables. The results are in this figure. The regions of the protein potentially influenced by the changes are annotated. The protein structure is adapted from Lemberg and Freeman ^1^. Information concerning the names used in the figure is provided in Table S2.

*Changes to the 5’ untranslated region of the transcript* - One of the most widely reported alternative splicing mechanisms is designed to control entry into translation or protein translation itself ^31^. This continues to be the case for the rhomboid genes examined here ( Table S3). Alternative splice variants with potential effects on translation were found in human, mouse and *Arabidopsis.* Twelve of the 13 human, and nine of the 13 mouse rhomboid genes were accompanied by entries with changes to the 5’ UTRs. Interestingly, despite the higher number of genes documented for *Arabidopsis*, 5’ UTR splice variants were found only for 7 of the 22 rhomboid genes. However, the absence of evidence for the other 15 *Arabidopsis* rhomboid genes may be due to unreported sequences or awaiting discovery, which was the case with At1g74130 and At1g25290 ^23, 24^.

*Changes to the amino terminal region* - Alternative splicing events affecting the amino termini appear to be common as well in our set of RNA sequences ( Figure 2– Figure 6 and Table S4). Changes affecting the region between the start methionine to the first predicted TMD are placed into this category which includes frame-shifts, alternate starting methionines, insertions and deletions. Changes in this region could affect functional aspects like protein targeting and transport, membrane insertion, assembly and topology, and assembly with complexes. With the exception of human RHBDD1, all of the other 12 human rhomboid genes are accompanied by alternative splice variants impacting their amino end sequences. The situation is slightly different in the mouse rhomboid system where 9 of the 13 genes contain variants impacting the amino end. Based on the mouse database, Rhbdf2, Rhbdd1 and Rhbdd3 do not so far have any variants impacting this region. Interestingly, only 3 *Arabidopsis* rhomboid genes have entries with predicted alternative splicing within the amino region - RBL4, RBL14 and RBL15. One *Drosophila* gene, Stet, has an altered amino terminus resulting in an alternative start methionine. Of the 5 *C. elegans* genes, one has an altered amino terminus due to alternative splicing. This ROM-4, displays a frame-shift that shortens the length of the region that could still possess functionality as with the case of At1g74130 ^24^.

*Changes to the L1 Loop region* – The L1 region contains a conserved “loop” structure. This structure lies either side of transmembrane helices and plays a role in rhomboid protease activity ^9^. The L1 loop is also partially inserted into the membrane ^32^. Site-directed mutagenesis of the conserved L1 loop residues revealed evidence that this loop controls the way in which rhomboids interact with lipid membranes ^32^. Our analyses of the Figure 1 entries indicate that the L1 Loop region is likely subject to alternative splicing ( Figure 2– Figure 6 and Table S5).

In human, splice variants involving the L1 loop accompany 9 different rhomboid genes. Generally, the outcomes of the variants range from deletion of residues from part of the L1 loop region, to altering a few residues at one end or the other of the structure.

A similar trend was found for 4 of the mouse rhomboid genes, Rhbdf1 (iRhom1), Rhbdd2, Derlin2, and Ubac2. Again, the two splice events resulted in the loss of residues from the L1 loop.

The *Arabidopsis* database contains splice variants of the L1 loop for two genes, RBL4 and RBL14. Like in human and mouse, alternative splicing resulted in the removal of residues or large sections of the L1 loop.

The *C. elegans* ROM-4 gene exhibits an alternate start methionine and a frame-shift within the L1 loop, giving rise to only the beginning part of the loop.

*Changes to regions affecting other structural aspects* - This category is defined as changes to the linker or other transmembrane regions (TMD) with no currently assigned functions, as opposed to the regions with distinct functions discussed above and below. Since changes to these regions, subtle or extensive, could potentially affect the other functional aspects of the protein itself, or its interactions, it would be important to assess these splicing events.

In human, 11 of the 13 rhomboid gene entries examined are accompanied by splice variants in regions of the protein that fall under this category. Examples being PARL variants lacking TMD3 and part of TMD4, or the amino end of TMD1 (see Figure 3– Figure 6 and Table S9).

*Changes to neighbouring regions of the catalytic dyad* - The catalytic sites of rhomboid proteases consist of residues contributed from two different transmembrane domains, TMD4 and TMD6, when using the “6+1” model ^1^. PARL and secretase-type catalytic residues are characterized by the amino acids GxSx – H. The catalytic residues of iRhoms are characterized by the residues GPxx – H.

There are currently 4 entries for human PARL rhomboids accompanied by splice variants that impact catalytic potential by altering the GASG or H sites through limited deletions, or extensions and the subsequent loss of the serine and glycine residues (GA SG to GA) ( Figure 2– Figure 6, Table S6).

Both human iRhoms, RHBDF1 and RHBDF2 (iRhom1 and iRhom2), contain frame shifts and early terminations within the L1 loop. RHBDL1 has a predicted variant resulting from a frame shift early in the transcript. The RHBDL1 frame shift alters the peptide sequence and removes the catalytic residues. The predicted mRNA/peptide for RHBDL3 displayed the same outcome as RHBDL1. The RHBDD1 gene is accompanied by two predicted forms with frame shifts and early terminations occurring before the catalytic residues.

In mice, there are 3 entries in our data set with splice variants that impact the catalytic residues. Rhbdf1 (iRhom1) is associated with extensive alternative splicing predictions. Nine different forms are predicted to impact the catalytic potential of the protein. Eight of these predictions display 2 additional residues within TMD4 (GPAG catalytic site), a loss of a residue within TMD6, and a changing of the histidine to a proline. The predicted transcript for the ninth form contains a frame shift in TMD2 that ultimately impacts the catalytic sites. Rhbdf2 has a predicted form with a frame shift in TMD2. Rhbdl3 gene is also accompanied by a splice variant with the potential to alter catalysis. Four of the seven forms result in a frame shift within TMD3 with resulting alterations to the catalytic regions. The resulting peptides remain out of frame, altering the peptide sequence downstream from TMD3.

In our *Arabidopsis* entries, 3 genes show evidence of altered catalytic potential through alternative splicing. One At1g74130 mRNA database entry exhibits a frame shift and early termination. The early termination resulted in the removal of the last TMD which basically eliminates the final catalytic residue. Two additional splice variants of At1g74130 were discovered experimentally by Powles *et al*. ^24^. These two variants displayed similar outcomes as the predicted form from the database entry above, namely early termination sites resulting in two different lengths at the carboxyl end of the protein. RBL3 (At5g07250) is accompanied by a form where the TMD containing the catalytic histidine is removed entirely. Although the Gate and the catalytic histidine residue are removed, the predicted carboxyl terminus of RBL3 is maintained in this RBL3 variant. The last *Arabidopsis* gene to highlight in this category is RBL 14 (At3g17611). RBL14 is accompanied by a splice variant that may alter the catalytic potential of this rhomboid by using an alternate start methionine located immediately after the catalytic GFSG residues.

*C. elegans* ROM-4 has an alternate starting methionine and a frame shift that results in the removal of the catalytic residues.

*Changes to neighbouring regions of the L5 Cap and the transmembrane domain 5 Gate (TMD5)* – Based on the 6+1 rhomboid protein model ^1^, transmembrane domain 5 (TMD5) is postulated to be a feature that controls the entry of a substrate into the active site - a gating control that determines enzymatic activity ^33, 34^. The Gate (TMD5) appears to be a region of potentially active alternative splicing activity ( Figure 2– Figure 6, Table S7).

Of the 13 human rhomboid genes with entries in our data set, 6 are accompanied by alterations to the Gating TMD. PARL, RHBDF1, RHBDL1, RHBDL3, DERL2 and DERL3 are accompanied by forms with altered TMD6 (based on the 1+6 model). The most common resulting event appears to be early termination of the protein.

The same splicing outcomes appear to be present in our set of entries for mouse, *Arabidopsis* and *C. elegans* rhomboids. Mouse Rhbdl3, Rhbdf1 and Rhbdf2 (iRhom1 and iRhom2) are accompanied by variants with deletions or early terminations caused by frame shifts. The *Arabidopsis* data set contains two genes with predicted alternative spliced sequences affecting the gating TMD region. *Arabidopsis* RBL1 possesses an insertion between the catalytic TMD4 and the linker to the Gate. The RBL3 variant is missing both the gating TMD5 and the catalytic TMD6. The *C. elegans* ROM-4 variant is also missing the gating TMD as a result of a frame shift.

*Changes to the carboxyl terminus region* - Most of the carboxyl termini changes are due to frame shifts, giving rise to different carboxyl sequences. In human, PARL, RHBDF2, RHBDL1, RHBDD1, and Derlin3, all contain variants with different carboxyl ends. The situation is similar in mice where Rhbdf1, Rhbdf2 and Rhbdl3 are accompanied by variants with different carboxyl ends ( Figure 2– Figure 6, Table S8).

In *Arabidopsis*, At1g74130 and At5g07250 variants have shortened carboxyl termini. The three alternative splice variants of At1g74130 lack the entire predicted carboxyl terminus. The introduction of a stop codon in TMD6 (or further upstream) resulted in the early termination of translation. The At5g07250 (RBL3) variant lacks TMD5 and TMD6, but the carboxyl terminus is restored with the removal of the first 4 residues of the predicted motif.

*Changes to the 3’ UTR region of the transcript* - There are also changes associated with 3’ UTR of rhomboid transcripts ( Table S10). In our set of entries, there are 9 human genes with splice variants in the 3’ UTR. Some of the variants possess longer 3’ UTR sequences, whereas others exhibit shorter 3’ UTRs.

A similar situation is observed for mice where 5 genes are associated with variants containing altered 3’ UTRs. Rhbdl2, Rhbdf1 and Rhbdf2 variants contain extended 3’ UTRs, whereas Rhbdl3 variants exhibit shorter 3’ UTRs.

The *Arabidopsis* genes At1g74130, At3g17611 and At3g58460 are accompanied by one variant each with shortened 3' UTRs. One other gene, At2g29050 (NM_001084504.1), is represented without a predicted 3' UTR in the database.

### What are the potential impacts on the rhomboid protein structure upon alternative splicing?

The data compiled above suggest potentially impactful changes to the functionality of the affected proteins, but this speculation is limited to linear protein sequences and motifs. We were next interested in testing out possible functionality changes using currently available predictive tools for 3-D protein structures, despite this highly speculative assessment tool. To this end, we decided to use the established 3-D structure/model of the bacterial rhomboid GlpG to test the potential impact exerted by the various splice variant types. The GlpG model is the most established of the rhomboids and offers a more complete structure for this analysis. It should be noted that there are caveats associated with the use of the bacterial GlpG to assess other rhomboid types, but this analysis is strictly focused on how changes could theoretically impact such a rhomboid structure. Because these assessments are judged as being too speculative, the outcomes are provided only as Supplementary Material ( Supplementary File 1 and Figure S1– Figure S8). These outcomes may be useful starting points for guiding future structural studies that validate the outcomes. The actual impacts to functionality and structure of particular alternatively spliced protein variants also need to be studied individually through experimentation. This notion was assessed here for a selection of splice variants using the different types of activity tests. These tests were devised mainly to uncover evidence of functionality in splice variant proteins, as opposed to assigning possible biological roles. Different contexts were used to assess functionality of the selected splice variants, contexts ranging from transgenic expression to assays using recombinant proteins as exogenous additives.

**Figure 7.**
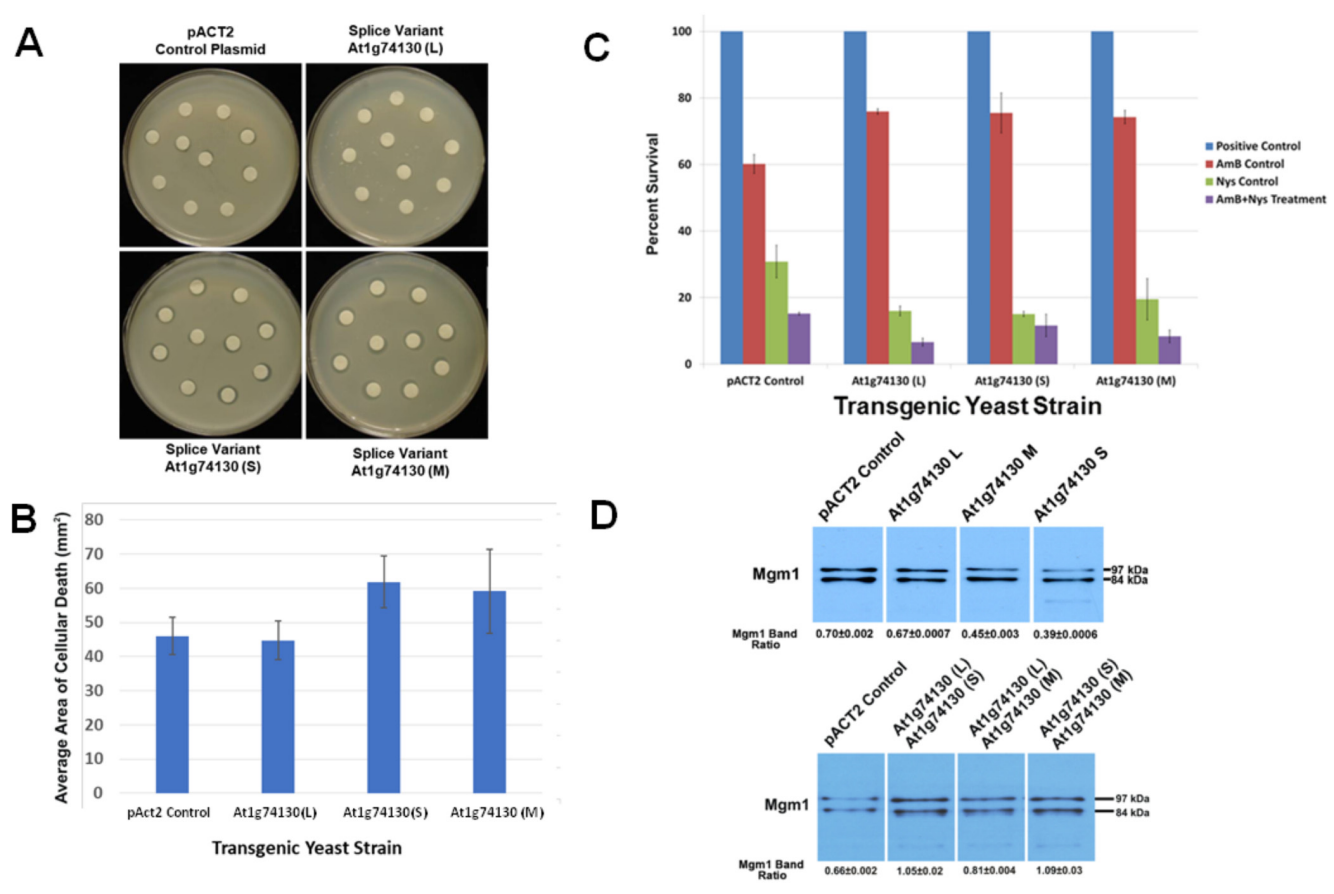
Activity assays for At1g74130 splice variant proteins using transgenic yeasts. ( **A**) Expression of At1g74130 splice variant proteins in yeast shows activity as increased nystatin sensitivity in a disk assay. Nystatin infused disks were applied on top of transgenic *S. cerevisiae* lines prepared as top agar cultures at a density of 5 × 10 ^7^ cells per mL. The transgenic yeast line being assessed is indicated: The pACT2 control plasmid line (top left), the line expressing the At1g74130 (L) variant (top right), the line expressing the At1g74130 (S) variant (bottom left) and the line expressing the At1g74130 (M) variant (bottom right). A representative experiment is presented in this panel. ( **B**) The areas of clearance (degree of cell death) around the nystatin infused disks were compared for the above set of plates (panel **A**). The pACT2 control line is labelled as pACT2 Control. The splice variant-expressing lines are labelled as in panel **A**. The average areas of clearance are shown with error bars (standard deviation) (n=10). ( **C**) Expression of At1g74130 variants in yeast also shows activity as changes to susceptibility to nystatin and amphotericin B. The same lines used in panels **A** and **B** were tested here. Each line was treated and then plated on appropriate agar media to determine susceptibility. The Percent Survival was calculated relative to untreated condition (untreated or mock treatments with no antifungals). The blue bars represent the control (no nystatin or amphotericin B), red bars represent cells treated with amphotericin B (AmB), green bars represent cells treated with nystatin (Nys) and the purple bars represent cells treated with both AmB and Nys. Error bars represent variation between two experiments. ( **D**) Analysis of possible interactions of between yeast Mgm1 and different combinations of At1g74130 splice variant proteins. The top row represents the Mgm1 levels in strains containing pACT2 or expressing one of the At1g74130 variant proteins (note that these immunoblotting results were reported previously from Powles *et al*. ^24^ and adapted here for comparative purposes). The bottom row of immunoblots depicts the changes in the ratio of Mgm1 (the uncleaved 97 kDa form versus the cleaved 84 kDa form) when various combinations of At1g74130 splice variant proteins were co-expressed. Each representative immunoblot is labelled accordingly. Only the relevant parts of the immunoblot images are displayed. The ratios were derived from two experiments and the bars represent variation between the two experiments. The immunoblot images used for analyzing the rhomboid combination experiments are provided in Figure S9.

The first series of tests were conducted for the *Arabidopsis* At1g74130 splice variants to obtain verification of functionality in a heterologous transgenic setting. For At1g74130, a functional relationship between its splice variants and a known yeast mitochondrial rhomboid substrate, Mgm1, was initially discovered using transgenic yeast ^24^. As shown previously in Powles *et al*. ^24^, each At1g74130 splice variant impacted the Mgm1 ratio (the amount of uncleaved (97 kDa) to cleaved (84 kDa)). The At1g74130 M and S splice variants individually reduced the ratio by about a third (from ratios of 0.67–0.7 to 0.45 for M and 0.39 for S) ^24^. Since the At1g74130 splice variants exist simultaneously in their natural *Arabidopsis* context, we further assessed different combinations of the same splice variants to see if such combined interactions influence the Mgm1 ratio, an indicator of splice variant activity. Further adjustments to the ratios by the various combinations of splice variants would uncover additional evidence of interaction and functionality. The results in Figure 7D ( Dataset 1 ^35^) indicate that various variant combinations possess the ability to influence the Mgm1 ratio, mainly more uncleaved Mgm1 (triggering higher ratios, from 0.81–1.09, instead of the ratios observed for cells expressing one variant at a time). The pACT2 control mitochondria displayed a similar Mgm1 ratio to that observed in Powles *et al*. ^24^, at 0.66 ±0.002 versus 0.70 ±0.002, respectively ( Figure 7D and Dataset 1 ^35^). Yeast cells expressing the pair At1g74130 (L) and (S) displayed a ratio of 1.05 ±0.02, cells expressing At1g74130 (L) and (M) exhibited a ratio of 0.81 ±0.004, and cells expressing At1g74130 (S) and (M) resulted in a ratio of 1.09 ±0.03 ( Figure 7D and Dataset 1 ^35^). The top set of ratios is provided and adapted from Powles *et al*. ^24^ to contrast the differences between single variant and double variant expression. These results demonstrate that the splice variants exhibit functionality in this setting.

We next assessed activity by looking at changes in sensitivity to the fungicide nystatin. Changes to sensitivity was assessed in two ways, growth/survival of yeast cells around nystatin-infused disks as a longer treatment strategy and nystatin treatments of cell cultures as a transient strategy. As shown in Figure 7A and B ( Dataset 1 ^35^), the expression of At1g74130 variants in yeast (especially (S) and (M)) increased nystatin sensitivity and cell death. The pACT2 control yeast strain and the strain expressing At1g74130 (L) displayed similar average areas of clearance (between 40–50 mm ^2^), whereas the strains expressing At1g74130 (S) and (M) frequently displayed larger clearance areas of approximately 60–70 mm ^2^ on average.

**Figure 8.**
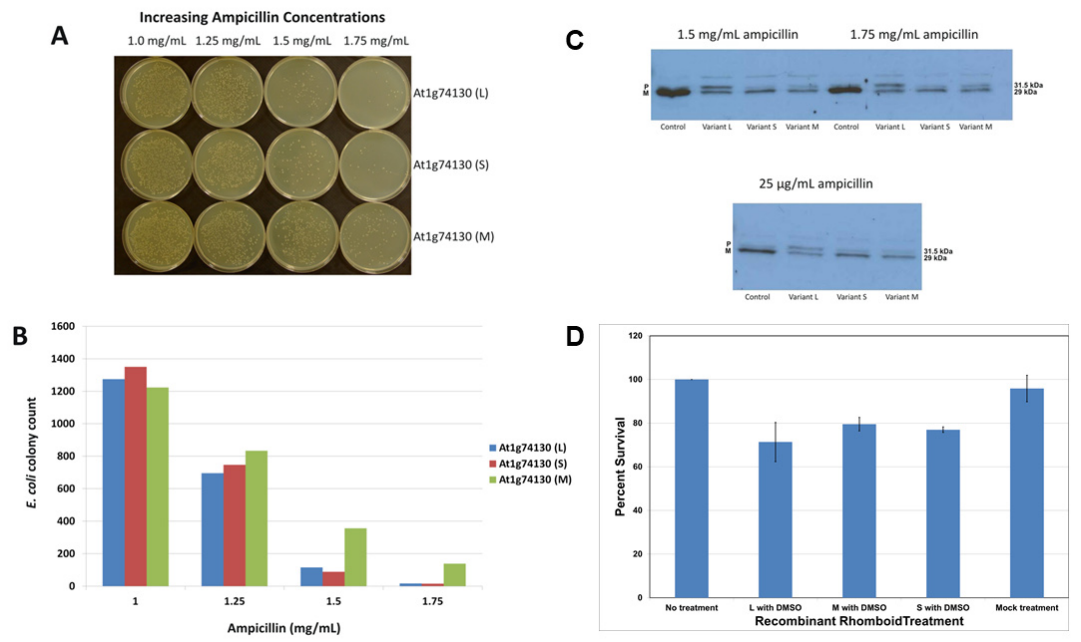
Activity assays for At1g74130 splice variant proteins using transgenic bacteria and as exogenous additives to “Superbug” bacteria. ( **A**) Bacterial cells expressing one of the three different splice variant proteins were grown on LB-ampicillin plates from 1.0 mg/mL to 1.75 mg/mL (labelled L, S, and M, as in the previous figures). ( **B**) Graphical representation of the survival levels observed for each of the plates shown in panel **A** (reflected as the numbers of colonies growing). The labelling is the same as in panel **A**. ( **C**) Expression of At1g74130 variant proteins in bacteria alters production and secretion of β-lactamase. Immunoblot images of β-lactamase protein samples are shown for different bacterial strains grown in different ampicillin concentrations. The lanes contain lysates from control cells containing pET20b only (labelled Control), and one of the At1g74130 variants, L, S, and M (labelled as Variant L, S, or M). The smaller-sized bands marked “M” represents the mature β-lactamase form (29 kDa) and the larger-sized bands marked “P” represents the precursor β-lactamase form (31.5 kDa). The full immunoblot image used is provided in Figure S10. ( **D**) Recombinant At1g74130 variant proteins were tested for activity (enhanced sensitivity to ampicillin in this case) as exogenous additives in the same manner as that shown for yeast in Figure 7. Cells (resulting colonies) surviving the different treatments are depicted as Percent Survival in the graph. The error bars represent standard deviations (n=3). The Percent Survival was calculated relative to the No Treatment control cell numbers. “No treatment” represents the bacterial culture used (diluted to the prescribed cell number tested as described in Methods). The variant protein being tested is labelled as in the panel **A**. DMSO indicates the use of DMSO (5% v/v) as the delivery agent. The Mock Treatment contains all components used except with no recombinant proteins added. All treatments involve exposure to 1.25 mg/mL ampicillin.

The disk assay results were reflected in the liquid culture assays. Yeasts expressing the same At1g74130 variants were treated transiently with nystatin and amphotericin B prior to plating. Compared to untreated cells, all strains experienced decreased survivability when treated with nystatin and/or amphotericin B ( Figure 7C and Dataset 1 ^35^). All cells displayed decreased survival when treated with amphotericin B. The pACT2 control was around 60.13 ±2.83% and the splice variant lines were around 74–76% (At1g74130 (L) line was at 75.91 ±0.83%, (S) line was at 75.49 ±5.99% and (M) line was at 74.26 ±2.04%). When treated with nystatin, all At1g74130 lines displayed decreased survivability relative to the control strain (pACT2 control line was at 30.82 ±4.85% compared to At1g74130 (L) line at 15.98 ±1.48%, (S) line at 15.07 ±0.77%, and (M) line at 19.52 ±6.25%). When treated with both amphotericin B and nystatin, the control line displayed 15.19 ±0.45% survival, whereas the splice variant lines were in the 6–12% range (At1g74130 (L) line at 6.62 ±1.20%, (S) line at 11.61 ±3.33%, and (M) line at 8.39 ±1.90%). Overall, with the exception of the amphotericin B alone treatment, cells expressing any of the three At1g74130 splice variants displayed lower survivability when treated transiently with fungicide. Both of the fungicide settings (panels A and B; Dataset 1 ^35^, and then panel C; Dataset 1 ^35^) indicate that the At1g74130 splice variants possess functionality. The phenomenon observed in transgenic yeast were also reflected in the transgenic bacteria setting, where ß-lactamase expression and secretion are high and considered to represent a “Superbug” (antibiotic resistance) model. Enhanced sensitivity to ampicillin was observed in bacteria expressing the At1g74130 (L) and (S) splice variants relative to (M) ( Figures 8A and B; Dataset 2 ^36^, respectively). Bacteria expressing At1g74130 (L) and (S) exhibited higher levels of sensitivity at lower ampicillin concentrations relative to (M) in this context. At the protein level, bacteria expressing At1g74130 splice variants displayed reduced synthesis, processing and secretion of β-lactamase ( Figure 8C). In cells with pET20b only, most of the ß-lactamase were present in the mature form (29 kDa) and at high total cell levels. In contrast, bacteria expressing At1g74130 splice variants exhibited shifts toward the precursor form (31.5 kDa), with (L) being the most impacted ( Figure 8C). Additionally, there were lower levels of β-lactamase overall in these same cells that may further contribute to the higher levels of sensitivity ( Figure 8C). These results indicate that the splice variants display functionality in this setting.

**Figure 9.**
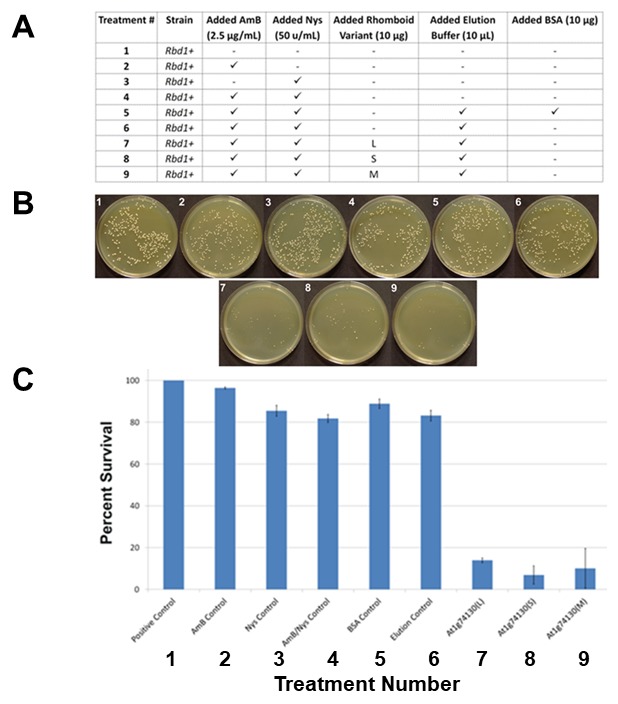
Activity assays using recombinant At1g74130 splice variant proteins as exogenous additives and yeast cells. ( **A**) The typical treatment matrix used in this study is shown. The check marks in the columns indicate the presence of a treatment component. The variant protein tested in Figure 7 is labelled only as L, S, and M in the matrix. The final treatment volume in all cases was 100 µL. ( **B**) The corresponding plate is shown for each of the treatments depicted in panel **A** (labelled according to treatment numbers 1 to 9). ( **C**) Cells (resulting colonies) surviving the different treatments are depicted as Percent Survival in the graph. The treatments are in the same order as presented in panels **A** and **B**. The error bars represent standard deviations (minimally n=3).

Evidence of functionality was also observed using an exogenous additive approach, where recombinant splice variant proteins were used to pre-treat cells before testing for changes to antimicrobial sensitivity (see Methods). This treatment scheme would be considered a transient strategy. For yeast cells, recombinant splice variants were delivered using a sub-lethal level of amphotericin B as the pre-treatment step before testing for sensitivity (survival) to nystatin. Even though amphotericin B is a fungicide (especially at higher levels), it was feasible to utilize amphotericin B at sub-lethal levels (1% (v/v)) for delivery purposes because this compound is capable of altering the permeability of fungal membranes and allow rhomboid proteins cellular access. The treatment matrix used for these yeast assays is shown in Figure 9A. The yeast strain tested here was the same parental host line used in the earlier assays. Sensitivity to nystatin was then tested at a level of 0.5% (v/v). Overall, treatment resulted in smaller colonies at the time of plate growth documentation (compare treatment 1 to treatments 2 to 9 in Figure 9B and C; Dataset 3 ^37^). All control or mock-type treatments display higher survival percentages compared to the three recombinant rhomboid pre-treatments ( Figures 9B and C; Dataset 3 ^37^). Relative to the Positive Control (considered 100% survival), the amphotericin B only treatment (AmB Control) displayed 96.46 ±0.43% survival, nystatin only (Nys Control) showed 85.46 ±2.60%, the amphotericin B-nystatin treatment (AmB/Nys Control) was at 81.81 ±1.86%, the amphotericin B-nystatin with BSA (BSA Control) was at 88.88 ±2.24% and amphotericin B-nystatin with protein elution buffer (Elution Control) was at 83.23 ±2.53%. Other components of the fungicides, such as the deoxycholate present in the amphotericin B solution, were also tested and found to have no impact on survivability at the levels used in these assays ( Figure S11). Finally, all three treatments with recombinant splice variants (which includes amphotericin B and nystatin) displayed decreased survivability (albeit at different levels between 6–14%) relative to control treatments (pre-treated with At1g74130 (L) displayed 13.93 ±1.16% survival, (S) at 6.92 ±4.39%, and (M) at 10.08 ±2.53%). Yeast cells pre-treated with recombinant At1g74130 variant proteins and then treated with both amphotericin B-nystatin exhibited the smallest colony sizes ( Figure 9B). Only pre-treatments with recombinant splice variants and amphotericin B resulted in higher sensitivity to nystatin. Protein pre-treatments without the delivery agent amphotericin B behaved like the controls ( Figure S11).

Changes to ampicillin sensitivity in bacteria were also observed using the transient approach. As a commonly used delivery component in many drug applications, DMSO was utilized here as the protein delivery agent in place of amphotericin B. Bacteria were pre-treated with DMSO and recombinant proteins before testing ampicillin sensitivity. Relative to the untreated (media only) and mock treatment (all components without recombinant proteins), pre-treatments with exogenously added At1g74130 splice variants decreased the number of colonies (a proxy for cells surviving the treatment) at the time of plate growth documentation ( Figure 8D and Dataset 2 ^36^). The mock treatment did not differ significantly (T-test, P = 0.36) from the no treatment control, indicating that the components used in the buffer did not contribute significantly to the enhanced level of ampicillin sensitivity. Relative to the mock treatment or no treatment control, the pre-treatment of bacteria with recombinant protein additives At1g74130 (L), (M), or (S), exhibited significant reductions in colony numbers (T-test: P = 0.022, P = 0.026, P = 0.029, respectively). The No Treatment control using 1.25 mg/ml ampicillin without DMSO, represents the reference point of 100% survival. The Mock Treatment without protein additives and with DMSO resulted in 95.85 ± 6.08% survival. The treatments (5% DMSO and 1.25 mg/ml ampicillin) and pre-treated with At1g74130 (L), (M), or (S) resulted in 71.36 ± 8.96%, 79.54 ± 3.10%, and 77.00 ± 1.27% survival, respectively.

The functionality assays used for At1g74130 were applied to splice variants from two other categories of rhomboid proteins. One splice variant pair was derived from *Arabidopsis* At1g25290 (named (L) and (S)) and another variant originated from human Ubac2. At1g25290 was from the “Active Rhomboid Proteases” category and Ubac2 is from the “Other Inactive Rhomboid Proteins” category. The overall results for variants from these two other categories indicate functionality as well in our assay settings (select assay results are reported here).

**Figure 10.**
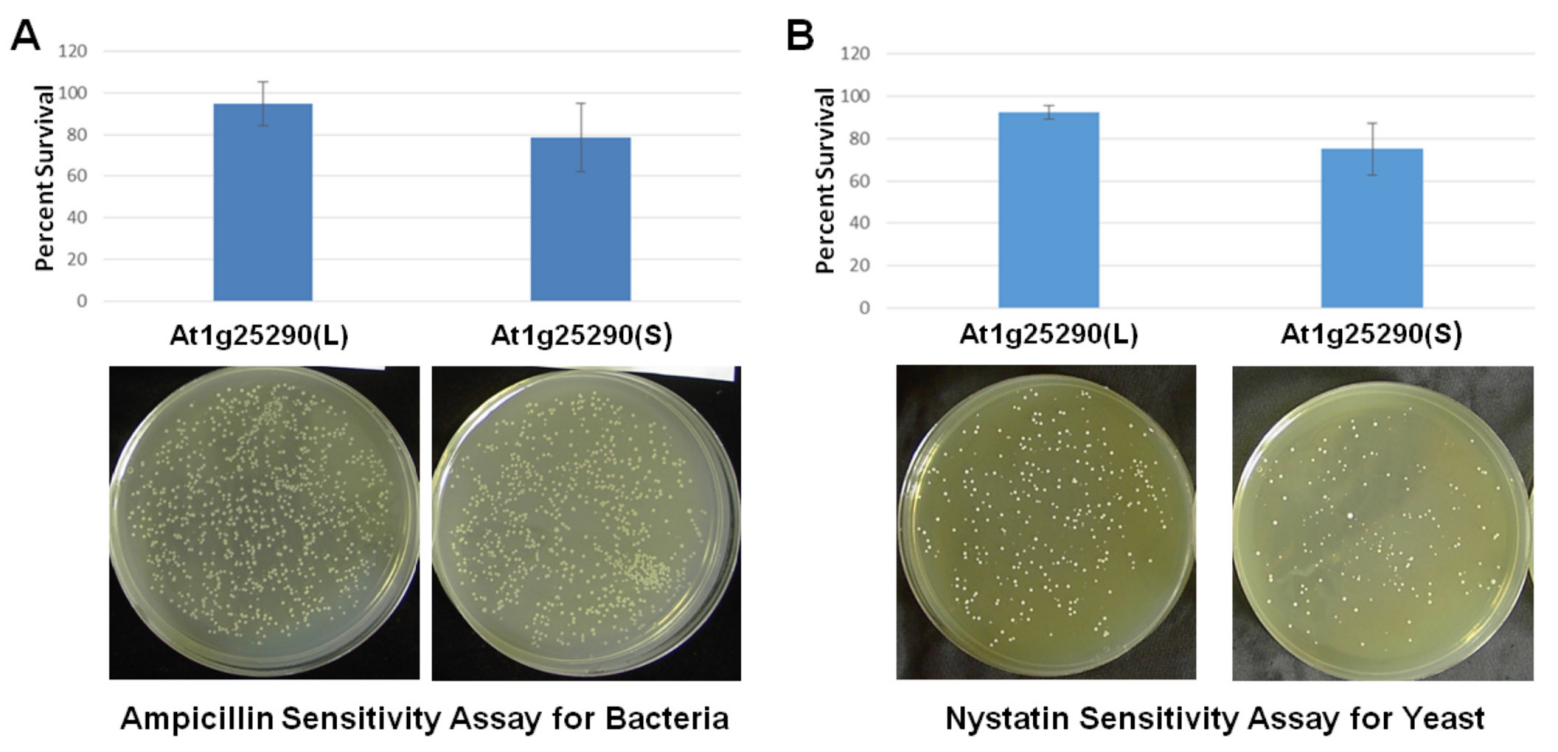
Activity assays using At1g25290 splice variant proteins as exogenous additives to bacteria and yeast cells. ( **A**) Recombinant At1g25290 splice variant proteins L and S were tested for activity (for enhanced sensitivity to ampicillin in this case) as exogenous additives and bacteria. The assays were conducted in the same manner as that shown in Figure 8 (from untreated to mock to added proteins). Cells (resulting colonies) surviving the different treatments were then assessed and represented as Percent Survival. The key results comparing the splice variants L and S are shown in this panel. The bar graphs are arranged to the corresponding representative results, the resulting agar plates. The error bars represent standard deviations (n=4, T-test, P=0.01). ( **B**) Recombinant At1g25290 splice variant proteins L and S were also tested for activity (for enhanced sensitivity to nystatin in this case) as exogenous additives and yeast. The assays were conducted in the same manner as that shown in Figure 9 (from untreated to mock to added proteins). The organization of panel **B** is the same as in panel **A**, except for yeast and nystatin. The error bars represent standard deviations (n=4, T-test, P=0.01).

For the At1g25290 splice variants (L) and (S), similar responses were observed in the exogenous additive-transient setting, albeit with differences in impact from that observed for the At1g74130 variants. Functionality was displayed in both bacterial ( Figure 10A and Dataset 4 ^38^) or yeast cell settings ( Figure 10B and Dataset 4 ^38^). The outcomes between the two At1g25290 splice variants were themselves different. The different sensitivity levels displayed by (S) relative to (L) suggest that the phenomenon observed is attributed to the added recombinant rhomboid variant (that their differences were derived from alternative splicing) and not to other components in the mixtures.

**Figure 11.**
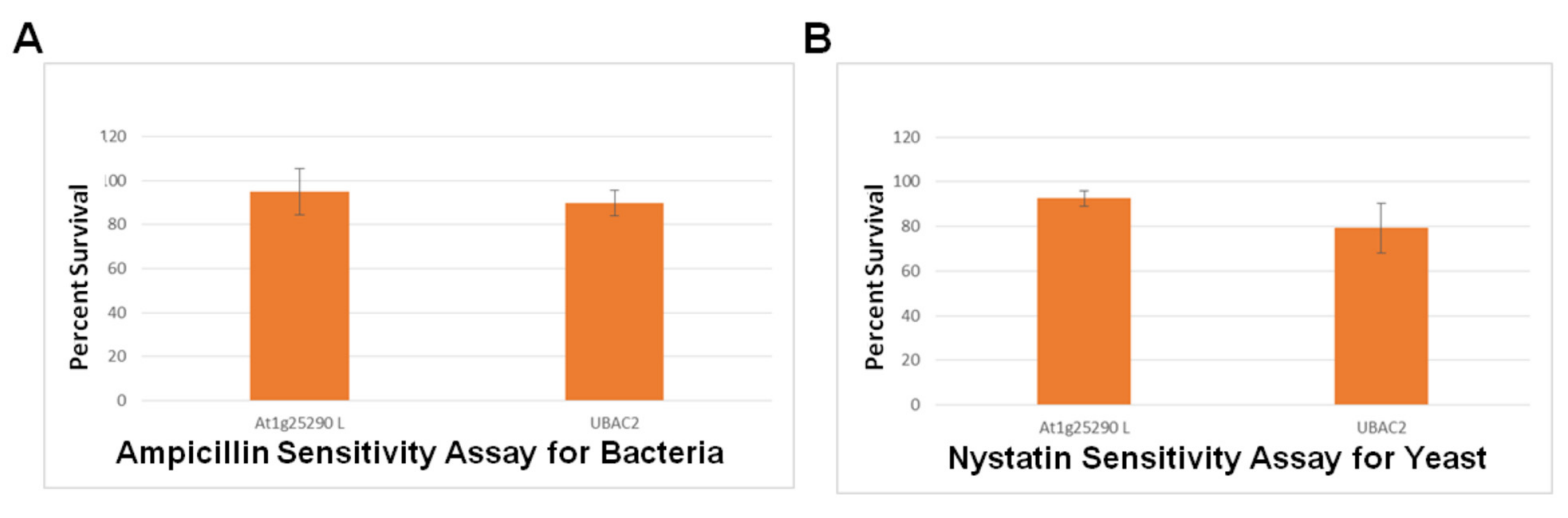
Activity assays using a select Ubac2 splice variant protein as exogenous additives to bacteria and yeast cells. ( **A**) Recombinant splice variant proteins At1g25290 (L) and one Ubac2 were tested for activity (for enhanced sensitivity to ampicillin in this panel) as exogenous additives and bacteria. The assays were conducted in the same manner as that shown in Figure 8 (from untreated to mock to added proteins). Cells (resulting colonies) surviving the different treatments were then assessed and represented as Percent Survival. The key results comparing the splice variants At1g25290 (L) and Ubac2 are shown in this panel. The error bars represent standard deviations (n=4, T-test, P=0.1). ( **B**) Recombinant At1g25290 splice variant protein L and Ubac2 were also tested for activity (for enhanced sensitivity to nystatin in this panel) as exogenous additives and yeast. The assays were conducted in the same manner as that shown in Figure 9 (from untreated to mock to added proteins). The organization of panel **B** of this figure is the same as in its panel **A**, except for yeast and nystatin. The error bars represent standard deviations (n=4, T-test, P=0.1).

The human Ubac2 splice variant tested is a fusion between a rhomboid protein sequence (considered a pseudoprotease) and ubiquitin-associating domains ^39, 40^. Like the above phenomenon, recombinant Ubac2 variant proteins showed functionality as an additive in bacterial and yeast assays, albeit at a more modest level of influence on antimicrobial sensitivity ( Figure 11; Dataset 5 ^41^).

In whole, not all variants will show functionality in the different test settings. The different assays does however provide evidence that splice variants from different rhomboid categories possess functionality, supporting the notion derived from the comparative analysis, that alternative splicing provides a mechanism for diversifying the numbers of working rhomboid proteins, and the roles of rhomboid proteins play in a particular system.

Raw data underlying the results in Figure 7 Figure 7B. Disk Assay Area Measurements of Growth (Sensitivity) in the Presence of Nystatin for Transgenic Yeast Lines Expressing At1g74130 Splice Variants; Figure 7C. Percent Survival (Cell/Colony Counts) of Various Transgenic Yeast Lines When Treated with Nystatin and Amphotericin B; Figure 7D. Densitometry Assessment of Immunoblots Developed for Assessing Mgm1 Levels and Ratios in Mitochondria Isolated from Transgenic Yeast Lines Expressing At1g74130 Splice Variants. Click here for additional data file. Copyright: © 2018 Powles J and Ko K 2018 Data associated with the article are available under the terms of the Creative Commons Zero "No rights reserved" data waiver (CC0 1.0 Public domain dedication).

Raw data underlying the results in Figure 8 Figure 8A–B. Cell/Colony Counts of Various Transgenic Bacterial Lines When Grown on Plates with Increasing Ampicillin Concentrations; Figure 8D. Assays to Determine Percent Survival (Cell/Colony Counts) of Bacterial Cells When Treated Exogenously with Recombinant At1g74130 Splice Variant Proteins. Click here for additional data file. Copyright: © 2018 Powles J and Ko K 2018 Data associated with the article are available under the terms of the Creative Commons Zero "No rights reserved" data waiver (CC0 1.0 Public domain dedication).

Raw data underlying the results in Figure 9 Figure 9C. Assays to Determine Percent Survival (Cell/Colony Counts) of Yeast Cells When Treated Exogenously with Recombinant At1g74130 Splice Variant Proteins. Click here for additional data file. Copyright: © 2018 Powles J and Ko K 2018 Data associated with the article are available under the terms of the Creative Commons Zero "No rights reserved" data waiver (CC0 1.0 Public domain dedication).

Raw data underlying the results in Figure 10 Figure 10A. Assays to Determine Percent Survival (Cell/Colony Counts) of Bacterial Cells When Treated Exogenously with Recombinant At1g25290 Splice Variant Proteins; Figure 10B. Assays to Determine Percent Survival (Cell/Colony Counts) of Yeast Cells When Treated Exogenously with Recombinant At1g25290 Splice Variant Proteins. Click here for additional data file. Copyright: © 2018 Powles J and Ko K 2018 Data associated with the article are available under the terms of the Creative Commons Zero "No rights reserved" data waiver (CC0 1.0 Public domain dedication).

Raw data underlying the results in Figure 11 Figure 11A. Assays to Determine Percent Survival (Cell/Colony Counts) of Bacterial Cells When Treated Exogenously with a Recombinant Ubac2 Splice Variant Protein; Figure 11B. Assays to Determine Percent Survival (Cell/Colony Counts) of Yeast Cells When Treated Exogenously with a Recombinant Ubac2 Splice Variant Protein. Click here for additional data file. Copyright: © 2018 Powles J and Ko K 2018 Data associated with the article are available under the terms of the Creative Commons Zero "No rights reserved" data waiver (CC0 1.0 Public domain dedication).

## Discussion and conclusions

Alternative splicing is used by many organisms to control and to diversify protein function. Historical examples include human tropomyosin, human kallikreins (secreted serine proteases), and fungal Ski7/Hbs proteins ^42– 44^. The same appears to be possibly happening with rhomboid genes, but despite witnessing alternative splicing as a mechanism for diversifying functionality in *Arabidopsis* and human breast cancer cells ^12, 18, 22– 24, 29, 45^, the number of demonstrated cases remains limited. We were thus interested in assessing how often this happens using the information available in the different genetic databases. Our comparative survey of current databases was focused specifically on functionality, as opposed to function, to capture slight changes (potential changes at this juncture) to functionality, such as those reported above ^23, 24^. We also wanted to determine the extent of alternative splicing within the different rhomboid gene systems of a particular eukaryotic organism as well as between different species. We thus limited our analysis only to splice variant entries in current RNA sequence databases of six eukaryotic organisms, ones used widely as experimental models. It was important to limit our analysis so that we can address the issue of extent and then assess how alternative splicing could be used to modify the functionality of distinct regions of the affected rhomboid proteins of our data set, especially regions with defined purposes. We then assessed the overall notion of the comparative findings by testing a selection of variant proteins from three different categories.

The overall evidence from the comparative analysis supports the hypothesis that alternative splicing is could be used to diversify rhomboid functionality in a number of cases. This is especially the situation in human, mouse, and *Arabidopsis*, organisms with relatively high numbers of rhomboid or rhomboid-like genes. Currently, there is a total of 95 entries for 13 human rhomboid genes that reflect alternative splice products, 53 for 13 mouse genes and 40 for 22 *Arabidopsis* genes. These splice variants were also not limited to a particular rhomboid category. The diversification appears to occur generally in distinct groupings across the entire rhomboid protein sequence. Although the comparative data suggest potentially impactful changes to functionality, this speculation remains limited to linear protein sequences and motifs. Therefore, we next tested the possible changes to functionality using currently available predictive tools for 3-D protein structures, despite the highly speculative nature of these tools. This analysis was focused strictly on how changes could theoretically impact such a rhomboid structure. Because these assessments are judged as being too speculative, the outcomes of these tests are provided only as Supplementary Material ( Supplementary File 1 and Figure S1– Figure S8). These outcomes may be useful for guiding future structural studies as well as validating the outcomes through extensive experimentation. The notion of diversification in functionality by alternative splicing mechanisms was tested experimentally using recombinant proteins of six different splice rhomboid variants from three different categories, Active Rhomboid Proteases, Inactive Rhomboid Proteins, and Other Inactive Rhomboid Proteins. These splice variants represent different structural changes from active sites, to truncations, to fusions.

Based on the compiled comparative data, some of the potential impacts were quite extensive and obvious. Some of the more obvious ones appear to arise from subtle changes to the protein sequence, such as the introduction or removal of a few residues. Many impacted important structural motifs, sometimes from afar or indirectly ( Figure 2– Figure 6 and Figure S1– Figure S8). Although the degree of amino acid sequence conservation of rhomboid and rhomboid-like proteins is relatively low between species and types, there are distinct conserved residues/motifs that serve the same important functions. Some of the functional motifs potentially impacted did include motifs of known functions like the L1 loop, the TMD5-L5 cap, and the catalytic dyad region. Such situations are likely to bear significant consequences with respect to functionality. For instance, the L1 loop has been the focus of several studies because it contains one of the conserved motifs outside of the catalytic cluster ^32^. Although the function of the L1 loop is not entirely understood, the importance of the loop on protease functionality was demonstrated by mutagenesis experiments ^9, 32^. Normally, the L1 loop is partially embedded in the membrane. Mutation of the conserved WR motif in the L1 loop of GlpG decreased proteolytic activity, suggesting a modulatory role for the loop ^32^. Additional evidence suggests a regulatory role for the L1 loop, and this role is linked to the formation of a rigid L1 loop structure ^32^. If the L1 loop serves as an anchor to the lipid bilayer, alterations to this structure could modulate the protein’s enzymatic activity with substrates. In 2007, Baker and coworkers ^34^ found an enhancement of proteolytic activity with their set of mutagenized L1 loop experiments, which further demonstrates the link between L1 loop and functionality. Such outcomes are certainly possible and were observed in the 3-D models predicted for the different splice variants tested here ( Figure S7).

Similar outcomes were also observed for splice variants involving changes to the L5 cap and TMD5 region ( Figure S8). In addition to experiments aimed at the L1 loop, Baker and coworkers ^34^ also carried mutagenesis-based experiments on the TMD5 region and observed enhancement of cleavage activity with some of the structural changes in TMD5. It was hypothesized that destabilization of the TMD5 helix allowed enhanced substrate entry ^34^. TMD5’s destabilization is believed to alter its configuration by changing its angle/tilt and proximity to neighboring helices. This in turn alters the efficiency of gating by this region. Alterations to the efficiency of gating then in turn affects proteolytic activity ^34^. This is because, normally, when the TMD5 is positioned in the 'open' conformation, the TMD5 helix pulls the L5 loop outward ^33^. The movement of the L5 cap structure allows substrate entry into the catalytic cavity. The open conformation of the L5 cap is also believed to permit the entry of water into the catalytic cavity ^33^. Therefore, alterations to TMD5 and the L5 cap could potentially change features that affect substrate entry. The predicted outcomes in the examples shown in Figure S8 could possibly manifest in a similar manner with equally consequential effects. The structural outcomes revealed in our theoretical assessment of splice variants are thus likely to represent a mechanism for diversifying rhomboid functionality, since these alternative splice variants likely exist in the organisms studied.

Based on a combination of the previous findings for the two *Arabidopsis* plastid rhomboids ^23, 24^, the human RHBDD2 ^22^, and the trends revealed in this study, the overall evidence suggests that alternative splicing is a functionally significant mechanism for diversifying rhomboid functionality. This means that splice variants of rhomboids and rhomboid-like proteins likely exist simultaneously in the cell or sub-cellular compartment. Like the two *Arabidopsis* plastid proteins, the alternative splice variants are likely co-expressed, modulated relative to each other to respond to the cell’s needs, and interacting in some manner. The possibility of interactions between rhomboid units themselves has been reported by Wu *et al*. ^33^ for the bacterial rhomboid protease GlpP. Such interactions with different rhomboid variants/forms and populations could therefore manifest in a number of ways that affect rhomboid functionality. The theoretical approach used in this study, and the predicted outcomes that may arise are thus not without merit, and should be considered as guidance for further experimentation. The possibilities, such as the ones discussed in the above examples, are observed experimentally in other studies and in functionality assays conducted for our select splice rhomboid variants. There are a number of other experimentally tested examples where truncations have been observed to impact functionality. In addition to rhomboid proteins, examples of other types of proteins include those recently reported by Stoddart *et al*. ^46^ for an integral membrane pore, and by Quemeneur *et al*. ^47^ where shape influences protein mobility within membranes. Whatever the situation may be for rhomboids, it is clear that it is necessary to characterize splice variants for each rhomboid and to determine how splicing influences rhomboid functionality. This would be important for elucidating how the different rhomboids work as a network.

## Data availability

The data referenced by this article are under copyright with the following copyright statement: Copyright: © 2018 Powles J and Ko K

Data associated with the article are available under the terms of the Creative Commons Zero "No rights reserved" data waiver (CC0 1.0 Public domain dedication). 

**Dataset 1: Raw data underlying the results in Figure 7.** Figure 7B. Disk Assay Area Measurements of Growth (Sensitivity) in the Presence of Nystatin for Transgenic Yeast Lines Expressing At1g74130 Splice Variants; Figure 7C. Percent Survival (Cell/Colony Counts) of Various Transgenic Yeast Lines When Treated with Nystatin and Amphotericin B; Figure 7D. Densitometry Assessment of Immunoblots Developed for Assessing Mgm1 Levels and Ratios in Mitochondria Isolated from Transgenic Yeast Lines Expressing At1g74130 Splice Variants. 10.5256/f1000research.13383.d204676 ^35^

**Dataset 2: Raw data underlying the results in Figure 8.** Figure 8A–B. Cell/Colony Counts of Various Transgenic Bacterial Lines When Grown on Plates with Increasing Ampicillin Concentrations; Figure 8D. Assays to Determine Percent Survival (Cell/Colony Counts) of Bacterial Cells When Treated Exogenously with Recombinant At1g74130 Splice Variant Proteins. 10.5256/f1000research.13383.d204677 ^36^

**Dataset 3: Raw data underlying the results in Figure 9.** Figure 9C. Assays to Determine Percent Survival (Cell/Colony Counts) of Yeast Cells When Treated Exogenously with Recombinant At1g74130 Splice Variant Proteins. 10.5256/f1000research.13383.d204678 ^37^

**Dataset 4: Raw data underlying the results in Figure 10.** Figure 10A. Assays to Determine Percent Survival (Cell/Colony Counts) of Bacterial Cells When Treated Exogenously with Recombinant At1g25290 Splice Variant Proteins; Figure 10B. Assays to Determine Percent Survival (Cell/Colony Counts) of Yeast Cells When Treated Exogenously with Recombinant At1g25290 Splice Variant Proteins. 10.5256/f1000research.13383.d204680 ^38^

**Dataset 5: Raw data underlying the results in Figure 11.** Figure 11A. Assays to Determine Percent Survival (Cell/Colony Counts) of Bacterial Cells When Treated Exogenously with a Recombinant Ubac2 Splice Variant Protein; Figure 11B. Assays to Determine Percent Survival (Cell/Colony Counts) of Yeast Cells When Treated Exogenously with a Recombinant Ubac2 Splice Variant Protein. 10.5256/f1000research.13383.d204681 ^41^

## Supplementary Material

Supplementary File 1: Supplementary notes for Figure S1 to Figure S8; References cited in the supplementary notes for Figure S1 to Figure S8; Supplementary figure legends for Figure S1 to Figure S11.

Supplementary Tables S1–10

Figure S1. Examples of alternative splicing and their potential impacts on the structure of the amino terminal region.

Figure S2. Examples of alternative splicing and their potential impacts on the structure of the carboxyl terminal region.

Figure S3. The potential impact of alternative splicing on the structure of the *Arabidopsis* rhomboid At3g17611.

Figure S4. The potential impact of alternative splicing on the structure of the *Arabidopsis* rhomboid At3g53780.

Figure S5. Examples of alternative splicing and their potential impacts on the L1 loop structure.

Figure S6. Examples of alternative splicing and their potential impacts on the L5 Cap-TMD5 structure.

Figure S7. The potential impact of alternative splicing on the structure of the *Arabidopsis* plastid rhomboid At1g25290.

Figure S8. Examples of alternative splicing and their potential impacts on the structure of the catalytic region.

Figure S9. Immunoblot images used for analyzing the impact of different rhomboid combinations on the Mgm1 ratios presented in Figure 7D.

Figure S10. Immunoblot images used for analyzing the impact of rhomboid variants on ß-lactamase production and secretion presented in Figure 9C.

Figure S11. Assay testing the combination of "Inactive" plastid rhomboid protein variants and deoxycholate in yeast without amphotericin B.
