# Supplementary material for: Alternative splice variants of rhomboid proteins: Comparative analysis of database entries for select model organisms and validation of functional potential
Source: F1000Res. 2018 May 31;7:139. Originally published 2018 Feb 1. [Version 2] doi: 10.12688/f1000research.13383.2 (PMC7065720; doi:10.12688/f1000research.13383.2)
Supplement: Supplementary file 6 [file f1000research-7-16431-s0006.tgz › 8cf8604d-cfa3-4cd1-ad91-89b7c86a00e2.docx]

**Alternative splice variants encoding rhomboid proteins: *In silico* analysis of database entries for select model organisms and validation of functional potential**

Joshua Powles, Kenton Ko

Department of Biology, Queen’s University, Kingston, Ontario, Canada K7L 3N6

**LIST OF SUPPLEMENTARY MATERIAL**

Table S1. List of alternative splice sequence entries sorted by model organisms.

Table S2. List of names and alternate names used for the various rhomboid genes and proteins.

Table S3. Summary of alternative splice variants impacting the 5’ UTR and translation.

Table S4. Summary of alternative splice variants impacting the amino terminus.

Table S5. Summary of alternative splice variants impacting the L1 loop region.

Table S6. Summary of alternative splice variants impacting regions of the catalytic dyad.

Table S7. Summary of alternative splice variants impacting the L5 cap region.

Table S8. Summary of alternative splice variants impacting the carboxyl terminus.

Table S9. Summary of alternative splice variants impacting the other regions and motifs.

Table S10. Summary of alternative splice variants impacting the 3’ UTR.

Supplementary notes for Figures S1 to S8.

References cited in the supplementary notes for Figures S1 to S8

Supplementary figure legends for Figures S1 to S8.

Figure S1. Examples of alternative splicing and their potential impacts on the structure of the amino terminal region.

Figure S2. Examples of alternative splicing and their potential impacts on the structure of the carboxyl terminal region.

Figure S3. The potential impact of alternative splicing on the structure of the *Arabidopsis* rhomboid At3g17611.

Figure S4. The potential impact of alternative splicing on the structure of the *Arabidopsis* rhomboid At3g53780.

Figure S5. Examples of alternative splicing and their potential impacts on the L1 loop structure.

Figure S6. Examples of alternative splicing and their potential impacts on the L5 Cap-TMD5 structure.

Figure S7. The potential impact of alternative splicing on the structure of the *Arabidopsis* plastid rhomboid At1g25290.

Figure S8. Examples of alternative splicing and their potential impacts on the structure of the catalytic region.

Figure S9. Immunoblot images used for analyzing the impact of different rhomboid combinations on the Mgm1 ratios presented in Figure 7D.

Figure S10. Immunoblot images used for analyzing the impact of rhomboid variants on ß-lactamase production and secretion presented in Figure 8C.

Figure S11. Assay testing the combination of "Inactive" plastid rhomboid protein variants and deoxycholate in yeast without amphotericin B.

**SUPPLEMENTARY NOTES FOR FIGURES S1 TO S8**

For this part of the study, we were interested in assessing theoretical changes that cover the range of splice variants, from subtle to extensive alterations of the rhomboid protein sequence. Subtle alterations typically involve the removal or addition of a few amino acids through events such as donor or acceptor site shifts. Extensive alterations may arise from events such as the use of alternative splice sites (predicted or cryptic), intron retention, or exon skipping. The results of this highly speculative analysis are provided in the Supplementary Material (Figures S1 to S8). Generally, our observation was that the splice variants reflect a mechanism for diversifying rhomboid functionality, subtly or extensively, in a number of cases studied here.

The first example to highlight involves changes with the potential to impact the L1 loop functionality (Figure S5). Based on current understanding of the GlpG rhomboid structure, the L1 loop is partially embedded in the membrane and mutations to its conserved WR motif decreases proteolytic activity (Wang and Ha, 2007). The L1 loop also appears to display a regulatory role, a role linked to the formation of its rigid structure (Wang and Ha, 2007). The L1 loop structures predicted here for the different splice variants display changes that could impact on the functionality of this loop. We observed in our highly speculative 3-D structure predictions where the shortening of the L1 loop sequence or re-positioning of the loop resulted in configurations that would likely reduce its capability to interact with the membrane and/or other neighboring domains/motifs. A collection of such predicted structures are shown in Figures S3 and S5 (*Arabidopsis* rhomboid splice variants without L1 loops).

Outcomes similar to the L1 loop were also observed for the L5 cap and TMD5 region (Figure S6). Normally, when the TMD5 is positioned in the 'open' conformation, the TMD5 helix pulls the L5 loop outward, allowing entry of water and a substrate into the catalytic cavity (Wu et al., 2006; Baker et al., 2007). This function is believed to be governed by TMD5’s angle/tilt and proximity to neighboring helices (Wu et al., 2006; Baker et al., 2007). Alterations to TMD5 and the L5 cap could thus potentially change structural features that affect substrate entry. This speculation was observed in the splice variants affecting TMD5-L5 cap (Figure S6). Changes such as an extra helix, flipped helix, or an extension of the L5 cap structure, were observed in the splice variants. These changes are structurally substantial and are likely to diversify rhomboid functionality.

Relatively simpler changes could also impact the functionality of the rhomboid protein. One observed example involves the addition or deletion of a few amino acids (Figure S7). In At1g25290, the addition or deletion of the RVL motif resulted in substantial changes to the loop structure between TMD3 and TMD4 (Figure S7). Part of the change in At1g25290 affects the top segment of TMD3, resulting in a longer TMD3 and a shorten loop. This shortened loop could possibly be more rigid in nature. Other examples in this category involve truncations at the carboxyl terminus that impact the regions involved with the catalytic dyad. Examples are shown for At1g74130 in Figure S8. These carboxyl truncations will likely affect how the other parts of the catalytic dyad regions work together.

Changes such as the ones predicted for the L1 loop are complex, involving many aspects of the protein, and are difficult to assess further in this study. Various aspects of these highly speculative impacts can only be uncovered and confirmed through detailed experimentation and assessments. In relatively simpler examples involving a RVL motif or catalytic dyad regions, the predicted impacts are more straightforward to visualize and appear to provide directly guidance for further experimentation.

**REFERENCES CITED IN THE SUPPLEMENTARY NOTES FOR FIGURES S1 TO S8**

Wang Y, Ha Y (2007) Open-cap conformation of intramembrane protease GlpG. Proc Nat Acad Sci USA 104: 1098-2102.

Wu Z, Yan N, Feng L, Oberstein A, Ya H, Baker RP, Gu L, Jeffrey PD, Urban S, Shi Y (2006) Structural analysis of a rhomboid family intramembrane protease reveals a gating mechanism for substrate entry. Nat Struct Mol Biol 13: 1084-1091.

Baker RP, Young K, Feng L, Shi Y, Urban S (2007) Enzymatic analysis of a rhomboid intramembrane protease implicates transmembrane helix 5 as the lateral substrate gate. Proc Natl Acad Sci USA 104: 8257-8262.

**SUPPLEMENTARY FIGURE LEGENDS**

**Figure S1.** **Examples of alternative splicing and their potential impacts on the structure of the amino terminal region.** (**A**) The theoretical impact of alternative splicing on the 3-D structure of a rhomboid protein was tested and depicted for different splice variants involving the amino terminal region. The areas of interest are highlighted by red arrows. (**B**) Amino acid sequence alignments of the proteins tested in A are shown. The amino terminal region involved is delineated by a red box. Other regions and motifs of the rhomboid protein are annotated and underlined. Key residues of the catalytic site are noted by colorized letters that match the colorized regions of the generated 3-D models.

**Figure S2.** **Examples of alternative splicing and their potential impacts on the structure of the carboxyl terminal region.** (**A**) The theoretical impact of alternative splicing on the 3-D structure of a rhomboid protein was tested and depicted for different splice variants involving the carboxyl terminal region. The areas of interest are highlighted by red arrows. (**B**) Amino acid sequence alignments of the proteins tested in A are shown. The carboxyl terminal region involved is delineated by a red box. Other regions and motifs of the rhomboid protein are annotated and underlined. Key residues of the catalytic site are noted by colorized letters that match the colorized regions of the generated 3-D models.

**Figure S3.** **The potential impact of alternative splicing on the structure of the *Arabidopsis* rhomboid At3g17611**. (**A**) The theoretical impact of alternative splicing on the 3-D structure of At3g17611 was tested and depicted for different splice variants. (**B**) Amino acid sequence alignments of the proteins tested in A are shown. Functional regions and motifs of the rhomboid protein are annotated and underlined. Key residues of the catalytic site are noted by colorized letters that match the colorized regions of the generated 3-D models.

**Figure S4.** **The potential impact of alternative splicing on the structure of the *Arabidopsis* rhomboid At3g53780**. (**A**) The theoretical impact of alternative splicing on the 3-D structure of At3g53780 was tested and depicted for two splice variants. (**B**) Amino acid sequence alignments of the proteins tested in A are shown. Functional regions and motifs of the rhomboid protein are annotated and underlined. Key residues of the catalytic site are noted by colorized letters that match the colorized regions of the generated 3-D models.

**Figure S5.** **Examples of alternative splicing and their potential impacts on the L1 loop structure.** (**A**) The theoretical impact of alternative splicing on the 3-D structure of a rhomboid protein was tested and depicted for different splice variants involving the L1 loop region. The areas of interest are highlighted by red arrows. (**B**) Amino acid sequence alignments of the proteins tested in A are shown. The L1 loop involved is delineated by a red box. Other regions and motifs of the rhomboid protein are annotated and underlined. Key residues of the catalytic site are noted by colorized letters that match the colorized regions of the generated 3-D models.

**Figure S6.** **Examples of alternative splicing and their potential impacts on the L5 Cap-TMD5 structure.** (**A**) The theoretical impact of alternative splicing on the 3-D structure of a rhomboid protein was tested and depicted for different splice variants involving the L5 Cap-TMD5 region. The areas of interest are highlighted by red arrows. (**B**) Amino acid sequence alignments of the proteins tested in A are shown. The L5 Cap-TMD5 region involved is delineated by a red box. Other regions and motifs of the rhomboid protein are annotated and underlined. Key residues of the catalytic site are noted by colorized letters that match the colorized regions of the generated 3-D models.

**Figure S7.** **The potential impact of alternative splicing on the structure of the *Arabidopsis* plastid rhomboid At1g25290.** (**A**) The theoretical impact of alternative splicing on the 3-D structure of At1g25290 was tested and depicted for splice variants involving the RVL motif (30). The areas of interest are highlighted by red arrows. (**B**) Amino acid sequence alignments of the proteins tested in A are shown. The RVL sequence involved is delineated by a red box. Other regions and motifs of the rhomboid protein are annotated and underlined. Key residues of the catalytic site are noted by colorized letters that match the colorized regions of the generated 3-D models.

**Figure S8**. **Examples of alternative splicing and their potential impacts on the structure of the catalytic region.** (**A**) The theoretical impact on the 3-D structure of a rhomboid protein was tested and depicted for different splice variants involving the catalytic region. The areas of interest are highlighted by red arrows. (**B**) Amino acid sequence alignments of the proteins tested in A are shown. The catalytic region involved is delineated by a red box. Other regions and motifs of the rhomboid protein are annotated and underlined. Key residues of the catalytic site are noted by colorized letters that match the colorized regions of the generated 3-D models.

**Figure S9. The immunoblot images used in Figure 7D are shown here.** Only relevant parts from one of the immunoblot images were used for the presentation in Figure 7D. These protein blots were probed with rabbit polyclonal antibodies against Mgm1. The splice variant combinations (or control) present in the yeast strain being analyzed are labelled accordingly in an abbreviated manner to Figure 7D.

**Figure S10. The immunoblot images used in Figure 8C are shown here.** Only relevant parts of the immunoblot images were used for the presentation in Figure 8C. These protein blots were probed with rabbit polyclonal antibodies against bacterial ß-lactamase (Antibodies-Online Cat# ABIN234380, RRID:AB_10770000).

**Figure S11. Assay testing the combination of "Inactive" plastid rhomboid protein variants and deoxycholate in yeast without amphotericin B**. (A) Plates containing yeast cells subjected to externally applied deoxycholate buffer (87.5 μg/mL sodium deoxycholate) and At1g74130 variant combinations (10 μg in 100 μL elution buffer with no amphotericin B). (B) Survival rates of yeast cells subjected to the various combinations depicted in panel A. The "Control" (no deoxycholate) columns/bars represent extracted buffer alone. The "no protein extract" columns/bars represent extract buffer (no added At1g74130 proteins) and deoxycholate buffer (mimicking the amphotericin B buffer). The deoxycholate buffer used here to mimic the possible amphotericin B buffer comprises of 50 mM sodium phosphate and 87.5 μg/mL sodium deoxycholate. This figure depicts one control assessment experiment prior to the test assays presented.
