# Supplementary figures and images for: Alternative splice variants of rhomboid proteins: Comparative analysis of database entries for select model organisms and validation of functional potential
Source: F1000Res. 2018 May 31;7:139. Originally published 2018 Feb 1. [Version 2] doi: 10.12688/f1000research.13383.2 (PMC7065720; doi:10.12688/f1000research.13383.2)

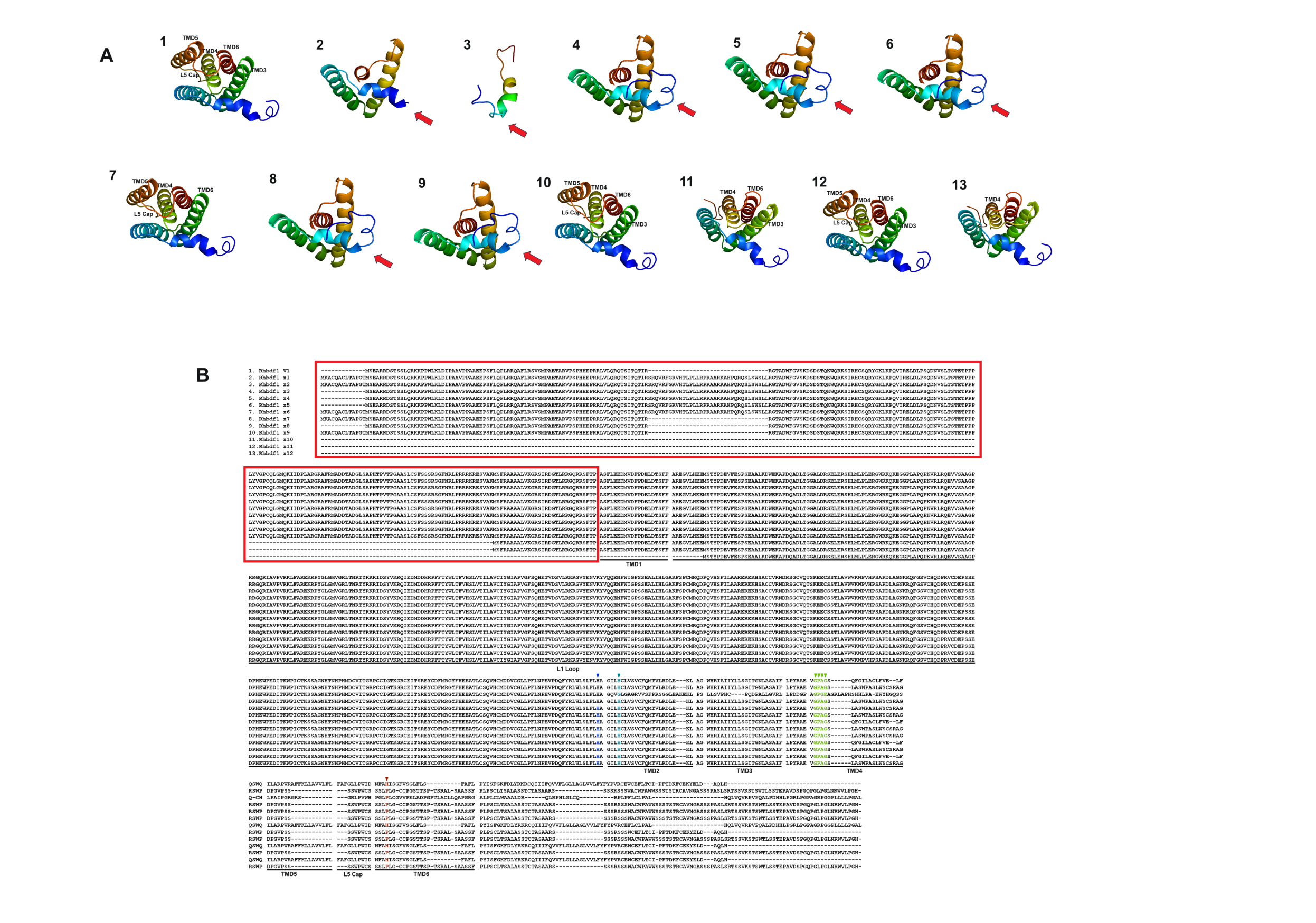

Supplement: Supplementary file 8 [file f1000research-7-16431-s0018.tgz › 0ff96a45-b5db-4c37-bcf3-75aef480320c.tif]

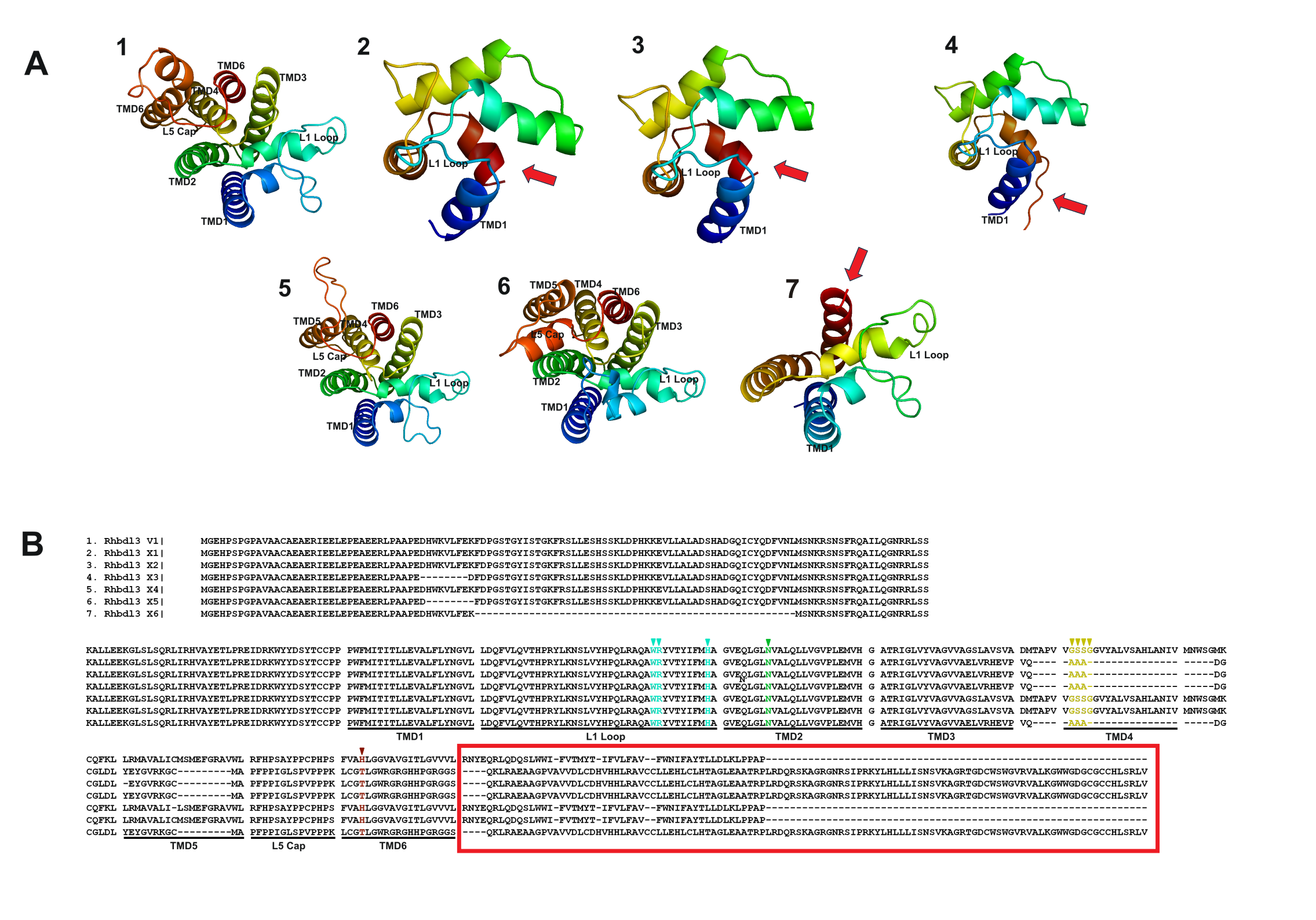

Supplement: Supplementary file 9 [file f1000research-7-16431-s0019.tgz › 91fd97ab-6af7-4d8b-bf9c-4d35e2f1416b.tif]

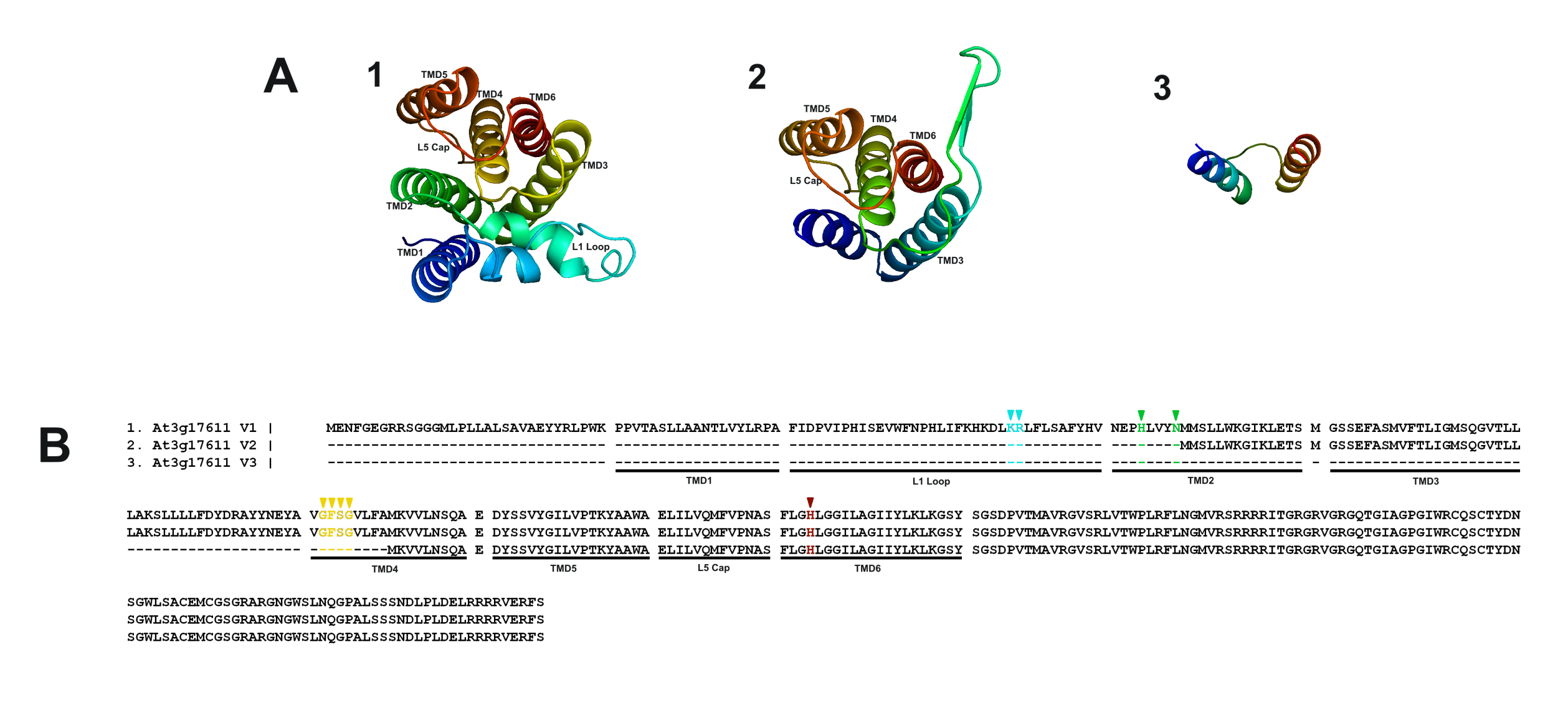

Supplement: Supplementary file 10 [file f1000research-7-16431-s0020.tgz › 8f8a8490-3870-4086-8068-2b3f75724818.tif]

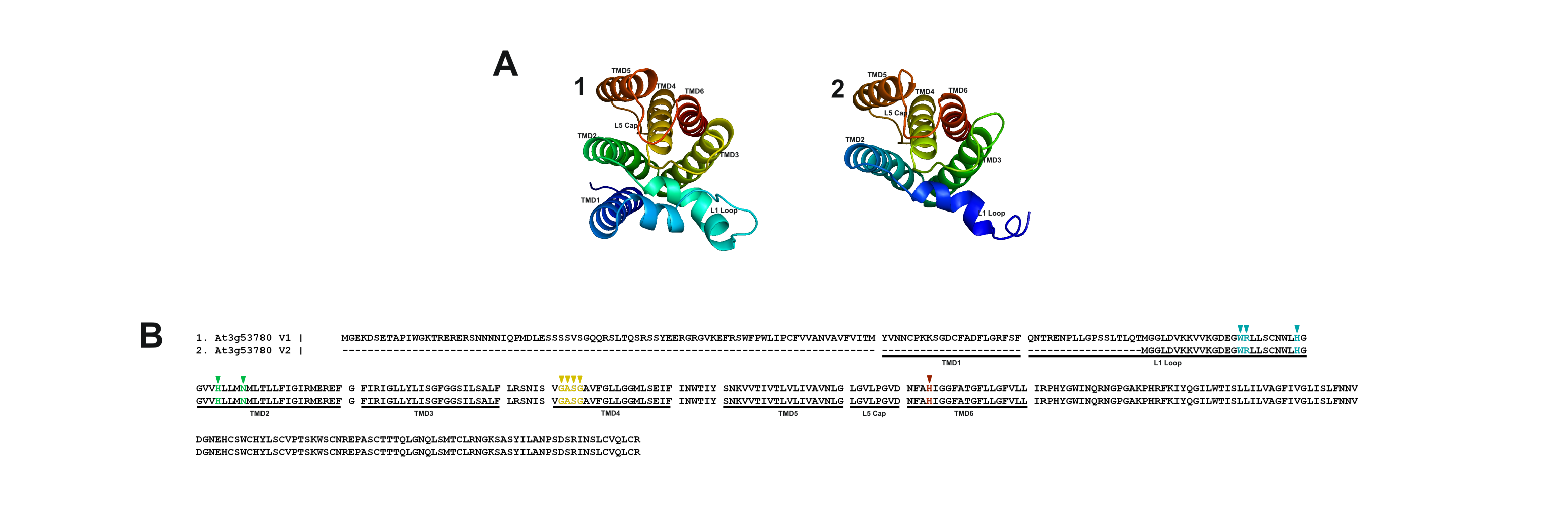

Supplement: Supplementary file 11 [file f1000research-7-16431-s0021.tgz › 697610ca-842d-4a5d-93aa-d6ed034c25f2.tif]

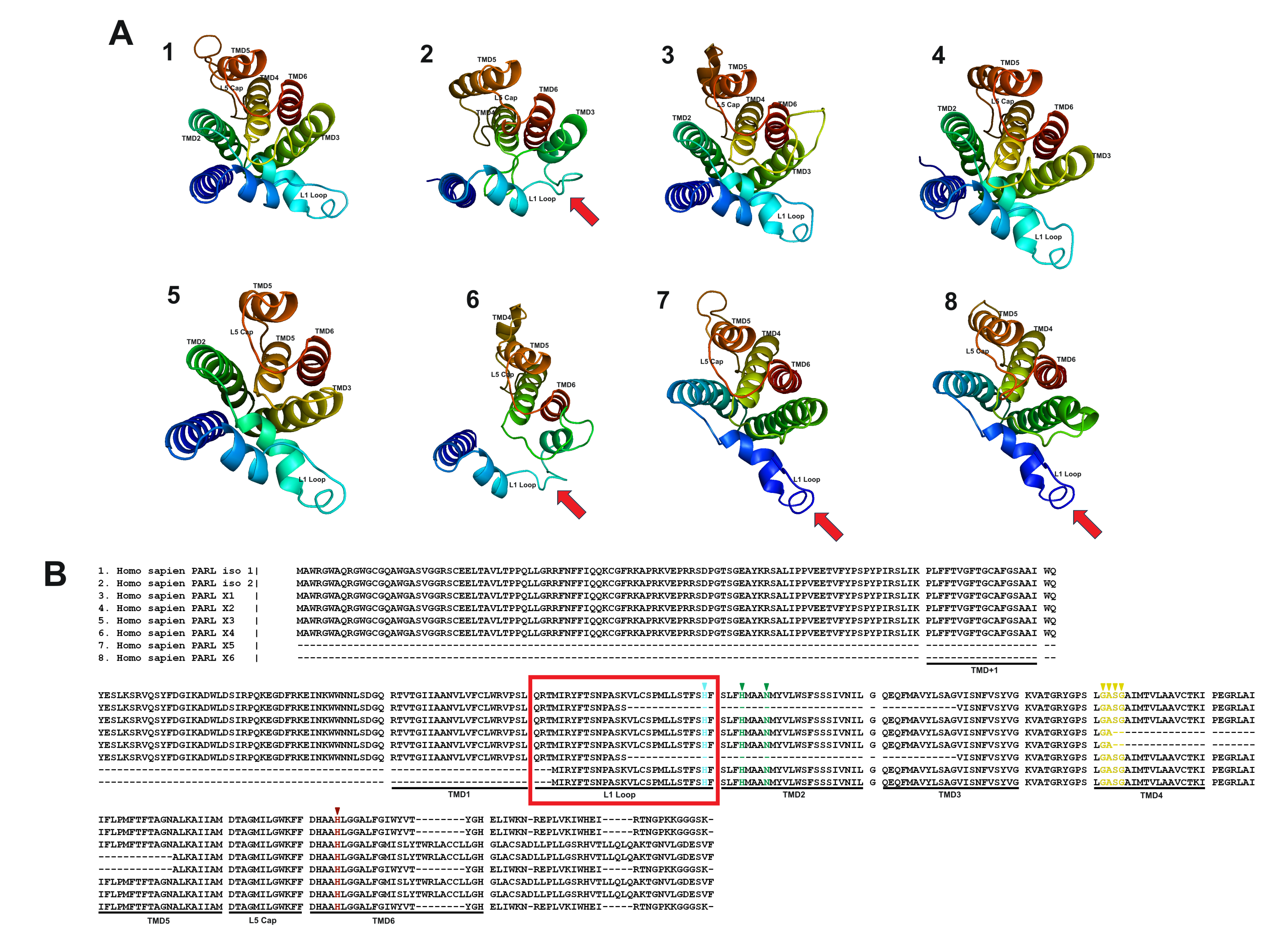

Supplement: Supplementary file 12 [file f1000research-7-16431-s0022.tgz › fe13871f-5e64-4aae-8408-87f3d2c83e84.tif]

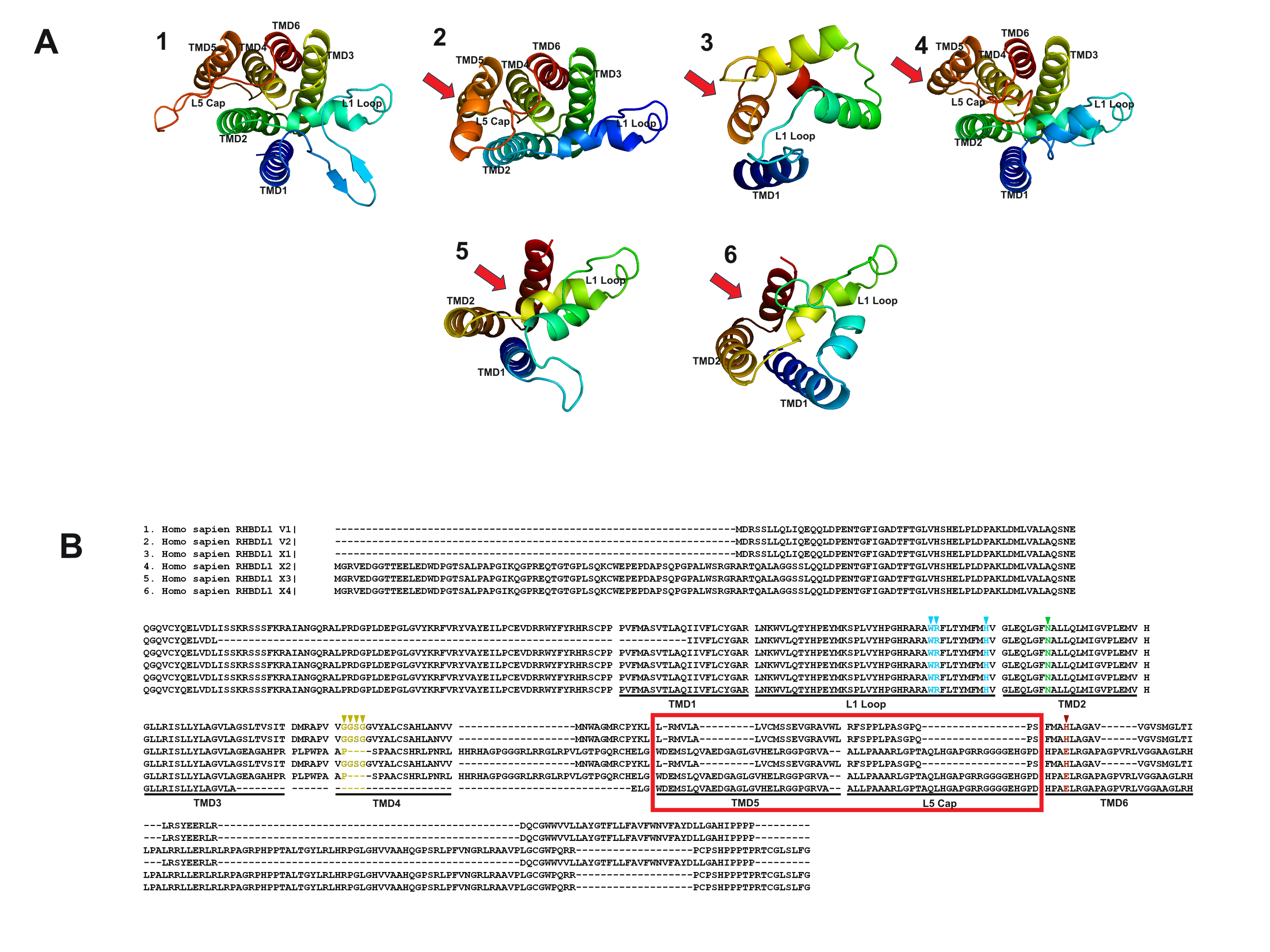

Supplement: Supplementary file 13 [file f1000research-7-16431-s0023.tgz › 52f647a3-bd1a-4267-b322-51ab9e4084e3.tif]

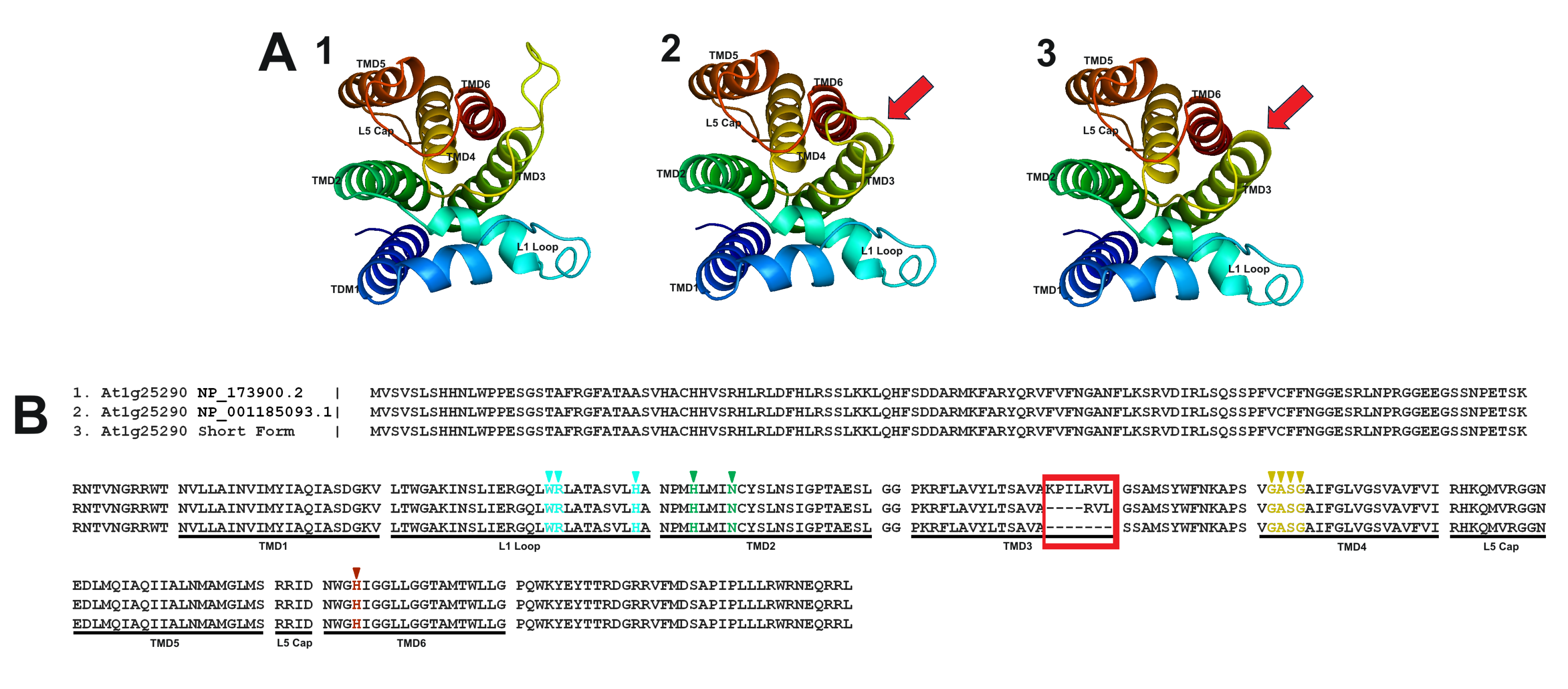

Supplement: Supplementary file 14 [file f1000research-7-16431-s0024.tgz › ecd4cd1f-372e-4bfc-8bbb-e11aa6da2b56.tif]

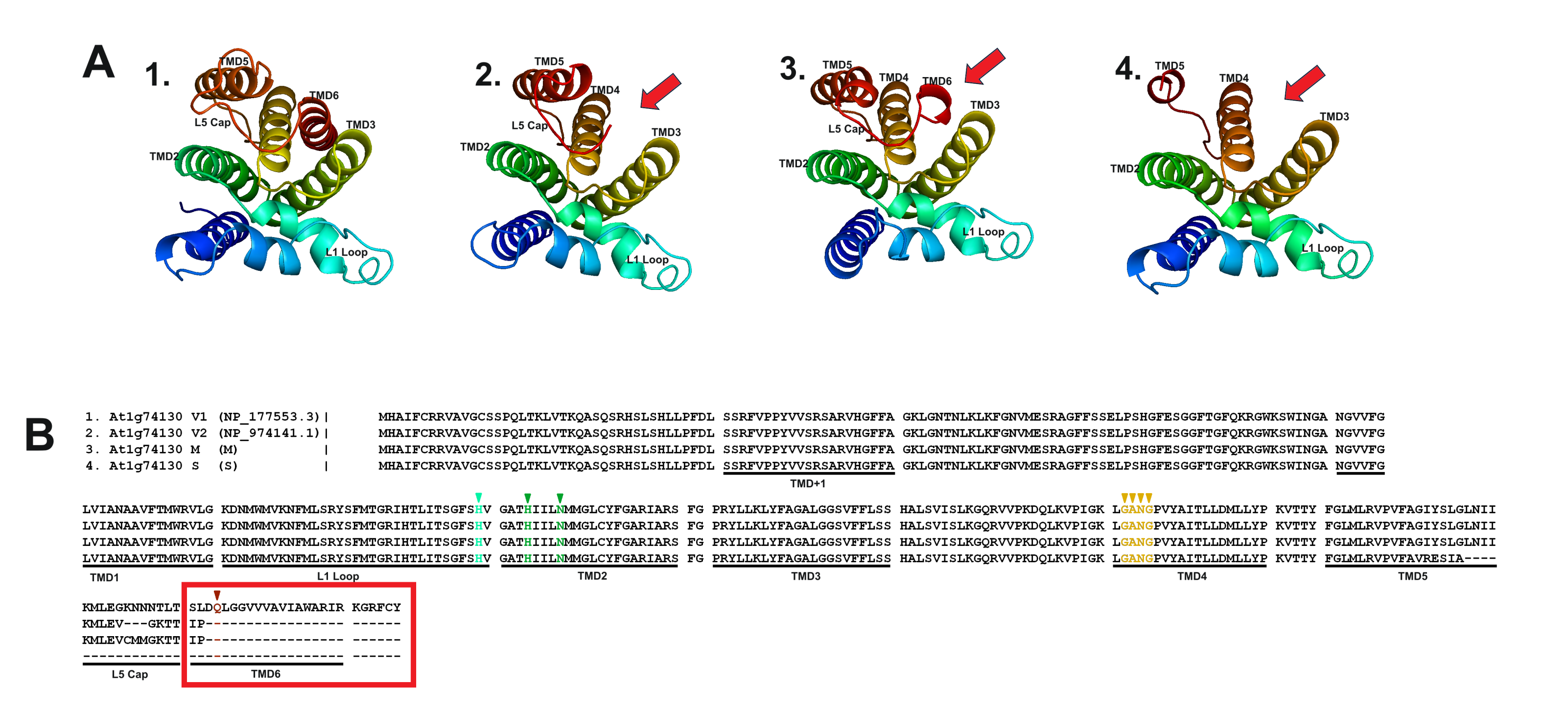

Supplement: Supplementary file 15 [file f1000research-7-16431-s0025.tgz › b6a3bf3a-233a-456f-b6e6-8072d4074cbd.tif]

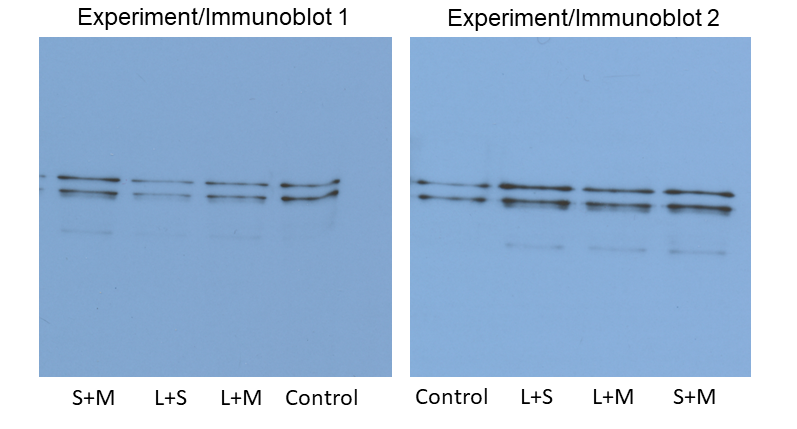

Supplement: Supplementary file 16 [file f1000research-7-16431-s0026.tgz › 4bbb527d-43ed-4f3a-942c-52302fac1250.tif]

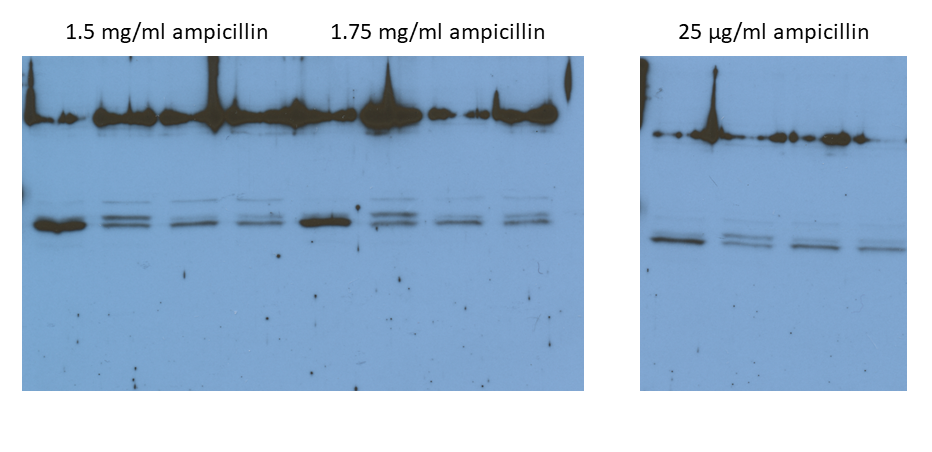

Supplement: Supplementary file 17 [file f1000research-7-16431-s0027.tgz › a6ded9c8-19da-4b63-b6cd-9ee9ef8fe2f0.tif]

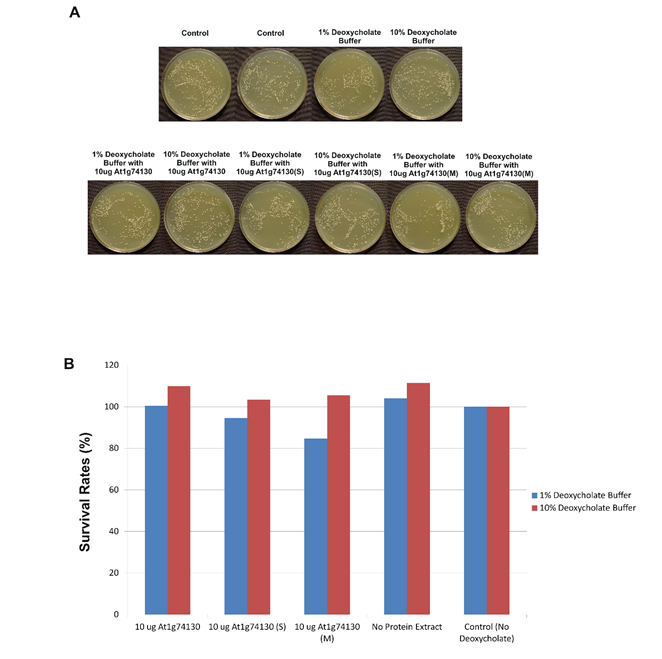

Supplement: Supplementary file 18 [file f1000research-7-16431-s0028.tgz › 5749e048-7239-4190-9452-716acb6fe95b.tif]
