# Supplementary material for: Alternative splice variants of rhomboid proteins: Comparative analysis of database entries for select model organisms and validation of functional potential
Source: F1000Res. 2018 May 31;7:139. Originally published 2018 Feb 1. [Version 2] doi: 10.12688/f1000research.13383.2 (PMC7065720; doi:10.12688/f1000research.13383.2)
Supplement: Supplementary file 7 [file f1000research-7-16431-s0007.tgz › cce5c9e8-3f8e-46f4-8069-4710eb69d9d2.zip!/Table S1.docx]

**Table S1. List of alternative splice sequence entries sorted by model organisms.**

The listed entries were analyzed and compiled after comparison to RNA sequence data as described in the methods. Note that some of the listed accession numbers are for the predicted sequences despite the comparative analysis.

| **Human** | | **Mouse** | | ***Arabidopsis*** | | ***Drosophila*** | | ***C. elegans*** | | ***S. cerevisiae*** | |
| --- | --- | --- | --- | --- | --- | --- | --- | --- | --- | --- | --- |
| PARL | NM_018622.6 | Parl | NM_001005767.4 | RBL1 At2g29050 | NM_128462.3 | rho (rhomboid) | NM_079159.3 | ROM-1 | NM_065628.2 | PCP1 | NM_001181230.1 |
|  | NM_001037639.2 |  | XM_006522312.1 |  | NM_001084504.1 |  | NM_001274326.1 | ROM-2 | NM_066719.2 | RBD2 | NM_001184060.1 |
|  | NM_001324436.1 | Rhbdf1 | NM_010117.2 | RBL2 At1g63120 | NM_104990.3 | ru (roughoid) | NM_080051.2 | ROM-3 | NM_070612.2 |  |  |
|  | NM_001324437.1 |  | XM_006514492.1 | RBL3 At5g07250 | NM_120807.3 | rho-4 | NM_167290.2 | ROM-4 | NM_001083250.4 |  |  |
|  | NM_001324438.1 |  | XM_006514493.1 |  | NM_001125710.1 | rho-5 | NM_205957.3 |  | NM_001047548.3 |  |  |
|  | XM_017006800.1 |  | XM_006514494.1 | RBL4 At3g53780 | NM_115238.2 | rho-6 | NM_176024.2 |  | NM_001047549.2 |  |  |
|  | XM_005247582.4 |  | XM_006514495.1 |  | NM_180367.1 | rho-7 | NM_078980.3 | ROM-5 | NM_058724.5 |  |  |
|  | XM_017006802.1 |  | XM_006514496.1 | RBL5 At1g52580 | NM_104136.2 |  |  |  |  |  |  |
|  | XM_017006801.1 |  | XM_006514497.1 | RBL6 At1g12750 | NM_101145.2 |  |  |  |  |  |  |
|  | XM_017006803.1 |  | XM_006514498.1 |  | NM_001084059.2 |  |  |  |  |  |  |
| RHBDF1 | NM_022450.3 |  | XM_006514499.1 |  | NM_001198046.1 |  |  |  |  |  |  |
|  | XM_005255494.1 |  | XM_006514500.1 | RBL7 At4g23070 | NM_118436.1 |  |  |  |  |  |  |
|  | XM_017023556.1 |  | XM_006514501.1 | RBL8 At1g25290 | NM_102339.3 |  |  |  |  |  |  |
|  | XM_006720921.1 |  | XM_006514502.1 |  | NM_001198164.1 |  |  |  |  |  |  |
|  | XM_017023557.1 |  | XM_006514503.1 |  | RVL alternate spliced |  |  |  |  |  |  |
|  | XM_005255498.1 | Rhbdf2 | NM_172572.3 | RBL9 At5g25752 | NM_147916.2 |  |  |  |  |  |  |
|  | XM_017023558.1 |  | NM_001167680.1 | RBL10 At3g17611 | NM_180275.2 |  |  |  |  |  |  |
| RHBDF2 | NM_001005498.3 |  | XM_006533108.1 |  | NM_202600.1 |  |  |  |  |  |  |
|  | NM_024599.5 | Rhbdl1 | NM_144816.1 |  | NM_001084701.1 |  |  |  |  |  |  |
|  | XM_011525250.2 |  | XM_006524021.1 | RBL11 At3g58460 | NM_115708.3 |  |  |  |  |  |  |
|  | XM_011525251.2 |  | XM_006524022.1 |  | NM_001203197.1 |  |  |  |  |  |  |
|  | XM_005257670.1 | Rhbdl2 | NM_183163.2 | RBL12 At3g59520 | NM_115814.2 |  |  |  |  |  |  |
|  | XM_017025079.1 |  | XM_006503025.1 | PARL At1g18600 | NM_101718.4 |  |  |  |  |  |  |
|  | XM_005257669.3 |  | XM_006503026.1 | At1g74130 | NM_106073.5 |  |  |  |  |  |  |
|  | XM_011525249.2 | Rhbdl3 | NM_139228.3 |  | NM_202413.1 |  |  |  |  |  |  |
| RHBDL1 | NM_001278720.1 |  | XM_006533326.1 |  | At1g74130(S) – 6/7 |  |  |  |  |  |  |
|  | NM_001278721.1 |  | XM_006533327.1 |  | At1g74130(M) – 7/8 |  |  |  |  |  |  |
|  | NM_001318733.1 |  | XM_006533328.1 | At1g74140 | NM_106074.2 |  |  |  |  |  |  |
|  | XM_017023849.1 |  | XM_006533329.1 |  | NM_001124127.1 |  |  |  |  |  |  |
|  | XM_017023850.1 |  | XM_006533330.1 |  | NM_001084350.2 |  |  |  |  |  |  |
| RHBDL2 | NM_001304746.1 |  | XM_006533331.1 |  | NM_001036203.1 |  |  |  |  |  |  |
|  | NM_017821.4 | Rhbdd1 | NM_029777.3 |  | NM_001084351.2 |  |  |  |  |  |  |
| RHBDL3 | NM_001330181.1 |  | NM_001122685.1 | At3g07950 | NM_111674.2 |  |  |  |  |  |  |
|  | NM_138328.2 |  | XM_006496574.1 | At5g25640 | NM_122475.1 |  |  |  |  |  |  |
|  | XM_017024272.1 | Rhbdd2 | NM_146002.2 | At5g38510 | NM_123212.2 |  |  |  |  |  |  |
|  | XM_017024275.1 |  | XM_006504416.1 |  | NM_001085212.1 |  |  |  |  |  |  |
|  | XM_017024273.1 |  | XM_006504417.1 | At1g77860 | NM_106435.2 |  |  |  |  |  |  |
|  | XM_017024276.1 | Rhbdd3 | NM_177370.3 | DER1 At4g29330 | NM_119078.4 |  |  |  |  |  |  |
|  | XM_011524443.2 |  | XM_006514713.1 | DER2.1 At4g21810 | NM_118301.6 |  |  |  |  |  |  |
|  | XM_017024279.1 |  | XM_006514714.1 | DER2.2 At4g04860 | NM_11672.3 |  |  |  |  |  |  |
|  | XM_006721734.3 |  | XM_006514715.1 |  |  |  |  |  |  |  |  |
|  | XM_017024274.1 |  | XM_006514716.1 |  |  |  |  |  |  |  |  |
|  | XM_017024274.1 |  | XM_006514717.1 |  |  |  |  |  |  |  |  |
|  | XM_017024277.1 |  |  |  |  |  |  |  |  |  |  |
|  | XM_017024280.1 |  |  |  |  |  |  |  |  |  |  |
| RHBDD1 | NM_001167608.1 |  |  |  |  |  |  |  |  |  |  |
|  | NM_032276.3 |  |  |  |  |  |  |  |  |  |  |
|  | XM_017005089.1 |  |  |  |  |  |  |  |  |  |  |
|  | XM_017005091.1 |  |  |  |  |  |  |  |  |  |  |
|  | XM_017005094.1 |  |  |  |  |  |  |  |  |  |  |
|  | XM_017005085.1 |  |  |  |  |  |  |  |  |  |  |
|  | XM_017005087.1 |  |  |  |  |  |  |  |  |  |  |
|  | XM_017005084.1 |  |  |  |  |  |  |  |  |  |  |
|  | XM_017005090.1 |  |  |  |  |  |  |  |  |  |  |
|  | XM_017005086.1 |  |  |  |  |  |  |  |  |  |  |
|  | XM_017005092.1 |  |  |  |  |  |  |  |  |  |  |
|  | XM_017005083.1 |  |  |  |  |  |  |  |  |  |  |
|  | XM_017005088.1 |  |  |  |  |  |  |  |  |  |  |
|  | XM_017005093.1 |  |  |  |  |  |  |  |  |  |  |
|  | XM_017005095.1 |  |  |  |  |  |  |  |  |  |  |
| RHBDD2 | NM_001040456.2 |  |  |  |  |  |  |  |  |  |  |
|  | NM_001040457.2 |  |  |  |  |  |  |  |  |  |  |
|  | NM_001346186.1 |  |  |  |  |  |  |  |  |  |  |
|  | NM_001346187.1 |  |  |  |  |  |  |  |  |  |  |
|  | NM_001346188.1 |  |  |  |  |  |  |  |  |  |  |
|  | NM_001346189.1 |  |  |  |  |  |  |  |  |  |  |
| RHBDD3 | NM_001329536.1 |  |  |  |  |  |  |  |  |  |  |
|  | NM_012265.2 |  |  |  |  |  |  |  |  |  |  |
|  | XM_017028750.1 |  |  |  |  |  |  |  |  |  |  |
|  | XM_006724224.3 |  |  |  |  |  |  |  |  |  |  |
|  | XM_017028749.1 |  |  |  |  |  |  |  |  |  |  |
| DERL-1 | NM_001134671.2 |  |  |  |  |  |  |  |  |  |  |
|  | NM_001330601.1 |  |  |  |  |  |  |  |  |  |  |
|  | NM_024295.5 |  |  |  |  |  |  |  |  |  |  |
|  | XM_006716657.1 |  |  |  |  |  |  |  |  |  |  |
| DERL-2 | NM_001304777.1 |  |  |  |  |  |  |  |  |  |  |
|  | NM_001304779.1 |  |  |  |  |  |  |  |  |  |  |
|  | NM_016041.4 |  |  |  |  |  |  |  |  |  |  |
| DERL-3 | NM_001002862.2 |  |  |  |  |  |  |  |  |  |  |
|  | NM_001135751.1 |  |  |  |  |  |  |  |  |  |  |
|  | NM_198440.3 |  |  |  |  |  |  |  |  |  |  |
|  | XM_011530506.2 |  |  |  |  |  |  |  |  |  |  |
|  | XM_017029082.1 |  |  |  |  |  |  |  |  |  |  |
|  | XM_017029080.1 |  |  |  |  |  |  |  |  |  |  |
|  | XM_017029079.1 |  |  |  |  |  |  |  |  |  |  |
|  | XM_017029078.1 |  |  |  |  |  |  |  |  |  |  |
|  | XM_011530505.2 |  |  |  |  |  |  |  |  |  |  |
|  | XM_017029081.1 |  |  |  |  |  |  |  |  |  |  |
| UBAC2 | NM_001144072.1 |  |  |  |  |  |  |  |  |  |  |
|  | NM_177967.3 |  |  |  |  |  |  |  |  |  |  |
|  | XM_011521082.2 |  |  |  |  |  |  |  |  |  |  |
|  | XM_006719948.2 |  |  |  |  |  |  |  |  |  |  |
|  | XM_011521083.2 |  |  |  |  |  |  |  |  |  |  |
|  | XM_011521084.2 |  |  |  |  |  |  |  |  |  |  |
|  | XM_017020553.1 |  |  |  |  |  |  |  |  |  |  |
