# Supplementary material for: Alternative splice variants of rhomboid proteins: Comparative analysis of database entries for select model organisms and validation of functional potential
Source: F1000Res. 2018 May 31;7:139. Originally published 2018 Feb 1. [Version 2] doi: 10.12688/f1000research.13383.2 (PMC7065720; doi:10.12688/f1000research.13383.2)
Supplement: Supplementary file 7 [file f1000research-7-16431-s0007.tgz › cce5c9e8-3f8e-46f4-8069-4710eb69d9d2.zip!/Table S10.docx]

**Table 10: Summary of alternative splice variants impacting the 3’ UTR.**

| **3' UTR** | |  |  |
| --- | --- | --- | --- |
| **Rhomboid Name (accession #'s) [total forms]** | **Species** | **Rhomboid Type** | **Effect of Splicing** |
| Isoform 5 (NM_001324438.1 /NP_001311367.1 ) [10 forms] | Human | PARL | 3' UTR extended with unique nucleotides |
| X1 PARL (XM_017006800.1 / XP_016862289.1) [10 forms] | Human | PARL | 3' UTR contained unique nucleotides |
| X2 PARL (XM_005247582.4 / XP_005247639.1) [10 forms] | Human | PARL | 3' UTR contained unique nucleotides |
| X3 PARL (XM_017006802.1 / XP_016862291.1) [10 forms] | Human | PARL | 3' UTR contained unique nucleotides |
| X4 PARL (XM_017006801.1 / XP_016862290.1) [10 forms] | Human | PARL | 3' UTR contained unique nucleotides |
| X5 PARL (XM_017006803.1 / XP_016862292.1) [10 forms] | Human | PARL | 3' UTR contained unique nucleotides |
|  |  |  |  |
| X6 RHBDF1 iRhom1 (XM_017023558.1/XP_016879047.1) [7 forms] | Human | iRhom (evolved from PARL) | Early termination and unique 3’ UTR |
|  |  |  |  |
| X1 RHBDL1 (XM_017023849.1/XP_016879338.1) [5 forms] | Human | Secretase-type (6+1) | Much shorter 3' UTR |
| X2 RHBDL1 (XM_017023850.1/XP_016879339.1) [5 forms] | Human | Secretase-type (6+1) | A frameshift resulted in much longer 3' UTR |
|  |  |  |  |
| X1 RHBDL3 (XM_017024272.1/XP_016879761.1) [14 forms] | Human | Secretase-type (6+1) | Shorter 3' UTR but same overall sequence |
| X2 RHBDL3 (XM_017024275.1/XP_016879764.1) [14 forms] | Human | Secretase-type (6+1) | Shorter 3' UTR but same overall sequence |
| X3 RHBDL3 (XM_017024273.1/XP_016879762.1) [14 forms] | Human | Secretase-type (6+1) | Shorter 3' UTR but same overall sequence |
| X4 RHBDL3 (XM_017024276.1/XP_016879765.1) [14 forms] | Human | Secretase-type (6+1) | Shorter 3' UTR but same overall sequence |
| X9 RHBDL3 (XM_017024274.1/XP_016879763.1) [14 forms] | Human | Secretase-type (6+1) | Shorter 3' UTR but same overall sequence |
| X10 RHBDL3 (XM_017024278.1/XP_016879767.1) [14 forms] | Human | Secretase-type (6+1) | Shorter 3' UTR but same overall sequence |
| X11 RHBDL3 (XM_017024277.1/XP_016879766.1) [14 forms] | Human | Secretase-type (6+1) | Shorter 3' UTR but same overall sequence |
| X12 RHBDL3 (XM_017024280.1/XP_016879769.1) [14 forms] | Human | Secretase-type (6+1) | Shorter 3' UTR but same overall sequence |
|  |  |  |  |
| X3 RHBDD1 (XM_017005094.1/XP_016860583.1) [15 forms] | Human | Secretase-type (basic) | Early termination resulted in a unique 3’ UTR |
| X12 RHBDD1 (XM_017005093.1/XP_016860582.1) [15 forms] | Human | Secretase-type (basic) | Early termination resulted in a unique 3’ UTR |
| X13 RHBDD1 (XM_017005095.1/XP_016860584.1) [15 forms] | Human | Secretase-type (basic) | Early termination resulted in a unique 3’ UTR |
|  |  |  |  |
| X1 DERL1 (XM_011517299.1/XP_011515601.1) [5 forms] | Human | Rhomboid pseudoprotease | Shorter sequence |
| X2 DERL1 (XM_006716657.1/XP_006716720.1) [5 forms] | Human | Rhomboid pseudoprotease | Shorter sequence |
|  |  |  |  |
| Isoform 2 DERL3 (NM_001002862.2/NP_001002862.1) [10 forms] | Human | Rhomboid pseudoprotease | Longer sequence |
| Isoform 3 DERL3 (NM_198440.3/NP_940842.2) [10 forms] | Human | Rhomboid pseudoprotease | Longer sequence |
| X1 DERL3 (XM_011530506.2/XP_011528808.1) [10 forms] | Human | Rhomboid pseudoprotease | Longer sequence |
| X3 DERL3 (XM_017029080.1/XP_016884569.1) [10 forms] | Human | Rhomboid pseudoprotease | Shorter and unique sequence |
| X4 DERL3 (XM_017029079.1/XP_016884568.1) [10 forms] | Human | Rhomboid pseudoprotease | Shorter and unique sequence |
| X5 DERL3 (XM_017029078.1/XP_016884567.1) [10 forms] | Human | Rhomboid pseudoprotease | Shorter and unique sequence |
| X6 DERL3 (XM_011530505.2/XP_011528807.1) [10 forms] | Human | Rhomboid pseudoprotease | Shorter and unique sequence |
| X7 DERL3 (XM_017029081.1/XP_016884570.1) [10 forms] | Human | Rhomboid pseudoprotease | Shorter and unique sequence |
|  |  |  |  |
| Isoform c DERL2 (NM_001304779.1/NP_001291708.1) [3 forms] | Human | Rhomboid pseudoprotease | Alternate termination resulted in longer 3’ UTR |
|  |  |  |  |
| X1 Rhbdd2(XM_006504416.1/XP_006504479.1) [3 forms] | Mouse | inactive distant relation | Significantly shorter - about 1/4 the length |
| X2 Rhbdd2(XM_006504417.1/XP_006504480.1) [3 forms] | Mouse | inactive distant relation | Significantly shorter - about 1/4 the length |
|  |  |  |  |
| X1 Rhbdf1 (XM_006514492.1/XP_006514555.1) [13 forms] | Mouse | iRhom | Coding sequence extended, 3'UTR wasreduced |
| X2 Rhbdf1 (XM_006514493.1/XP_006514556.1) [13 forms] | Mouse | iRhom | 3'UTR was larger, extending into previously coding nucleotides |
| X3 Rhbdf1 (XM_006514494.1/XP_006514557.1) [13 forms] | Mouse | iRhom | Coding sequence extended, 3'UTR was reduced |
| X4 Rhbdf1 (XM_006514495.1/XP_006514558.1) [13 forms] | Mouse | iRhom | Coding sequence extended, 3'UTR was reduced |
| X5 Rhbdf1 (XM_006514496.1/XP_006514559.1) [13 forms] | Mouse | iRhom | Coding sequence extended, 3'UTR was reduced |
| X7 Rhbdf1 (XM_006514498.1/XP_006514561.1) [13 forms] | Mouse | iRhom | Coding sequence extended, 3'UTR was reduced |
| X8 Rhbdf1 (XM_006514499.1/XP_006514562.1) [13 forms] | Mouse | iRhom | Coding sequence extended, 3'UTR was reduced |
| X10 Rhbdf1 (XM_006514501.1/XP_006514564.1) [13 forms] | Mouse | iRhom | Coding sequence extended, 3'UTR was reduced |
| X12 Rhbdf1 (XM_006514503.1/XP_006514566.1) [13 forms] | Mouse | iRhom | Coding sequence extended, 3'UTR was reduced |
|  |  |  |  |
| X1 Rhbdf2 (XM_006533108.1/XP_006533171.1) [3 forms] | Mouse | iRhom | 3'UTR was longer, extending into previously coding nucleotides |
|  |  |  |  |
| X1 Rhbdl2 (XM_006503025.1/XP_006503088.1) [3 forms] | Mouse | Secretase-type (6+1) | Extended 3'UTR |
| X2 Rhbdl2 (XM_006503026.1/XP_006503089.1) [3 forms] | Mouse | Secretase-type (6+1) | Extended 3'UTR |
|  |  |  |  |
| X1 Rhbdl3 (XM_006533326.1/XP_006533389.1) [7 forms] | Mouse | Secretase-type (6+1) | Coding region slightly extended, slightly shorter 3' UTR |
| X2 Rhbdl3 (XM_006533327.1/XP_006533390.1) [7 forms] | Mouse | Secretase-type (6+1) | Coding region slightly extended, slightly shorter 3' UTR |
| X3 Rhbdl3 (XM_006533328.1/XP_006533391.1) [7 forms] | Mouse | Secretase-type (6+1) | Coding region slightly extended, slightly shorter 3' UTR |
| X6 Rhbdl3 (XM_006533331.1/XP_006533394.1) [7 forms] | Mouse | Secretase-type (6+1) | Coding region slightly extended, slightly shorter 3' UTR |
|  |  |  |  |
| At1g74130 (NM_202412.1/NP_974141.1) [4 forms] | Arabidopsis | inactive homologue | Coding region was now 3'UTR and now longer |
|  |  |  |  |
| At2g29050 (NM_001084504.1/NP_001084504.1) [2 forms] | Arabidopsis | Secretase-type (6+1) | No 3' UTR on the sequence |
|  |  |  |  |
| At3g17611 (NM_001084701.1/NP_001078170.1) [3 forms) | Arabidopsis | Secretase (basic) | 3'UTR terminated earlier resulting in a slightly shorter region |
|  |  |  |  |
| At3g58460 (NM_001203197.1/NP_001190126.1) [2 forms] | Arabidopsis | Secretase (basic) | 3'UTR terminated earlier resulting in a slightly shorter region |
|  |  |  |  |
| rho (NM_079159.3/NP_523883.2) | Drosophila | Secretase | 3'UTR shortened to about 3/4 the length |
