# Supplementary material for: Alternative splice variants of rhomboid proteins: Comparative analysis of database entries for select model organisms and validation of functional potential
Source: F1000Res. 2018 May 31;7:139. Originally published 2018 Feb 1. [Version 2] doi: 10.12688/f1000research.13383.2 (PMC7065720; doi:10.12688/f1000research.13383.2)
Supplement: Supplementary file 7 [file f1000research-7-16431-s0007.tgz › cce5c9e8-3f8e-46f4-8069-4710eb69d9d2.zip!/Table S2.docx]

**Table S2: List of preferred and alternate names used for the various rhomboid genes and proteins.**

Details concerning the preferred rhomboid names and alternate names used are provided in the table. The table also includes Gene ID numbers and sample reference(s) for the naming of each rhomboid. The table is further sectioned into model organisms. The list of references cited follows the table.

| **Species** | **Preferred Name** | **Gene ID** | **Alternative Name(s)** | **Sample Reference(s)** |
| --- | --- | --- | --- | --- |
| **Human** | | | | |
|  | PARL | 55486 |  | Pellegrini *et al*., 2001; Curran *et al*., 2010; Meissner *et al*., 2011 |
|  | RHBDF1 | 64285 | Rhomboid 5 homolog 1; iRhom1 | Nakagawa *et al*., 2005 |
|  | RHBDF2 | 79651 | Rhomboid 5 homolog 2; iRhom2; veinlet-like 6 | Lemberg and Freeman, 2009; Wojnarowicz *et al*., 2012; Saarinen *et al*., 2012; Blaydon *et al*., 2012 |
|  | RHBDL1 | 9028 | Rhomboid 1; veinlet-like 1; RHBDL; RRP | Pascall and Brown, 1998; Urban *et al*., 2001; Etheridge *et al*., 2013 |
|  | RHBDL2 | 54933 | Rhomboid 2; veinlet-like 2; RRP2 | Adraian *et* al., 2011; Lei and Li, 2009; Urban *et al*., 2001 |
|  | RHBDL3 | 162494 | Rhomboid 3; veinlet-like 3; RHBDL4; VRHO; ventrhoid; RRP3 | Jaszai and Brand, 2002; Lohi *et al*., 2004 |
|  | RHBDD1 | 84236 | Rhomboid domain containing 1; RHBDL4 | Koonin *et al*., 2003; Liu *et al*., 2013; Wan *et al*., 2012; Wang *et al*., 2008 |
|  | RHBDD2 | 57414 | Rhomboid domain containing 2; veinlet-like 7; RHBDL7 | Abba *et al*., 2009; Canzoneri *et al*., 2014; Lacunza E *et al*., 2012; Lemberg and Freeman, 2009 |
|  | RHBDD3 | 25807 | Rhomboid domain containing 3 ; PTAG | Bahar *et* al., 2007; Lemberg and Freeman, 2009 |
|  | DERL1 | 79139 | Derlin-1; DER-1; DER1 | Chen *et* al., 2013; Katiyar *et al*., 2005; Nishitoh *et al*., 2008; Sun *et al*., 2008; Wang *et al*., 2008; Ye *et al*., 2004 |
|  | DERL2 | 51009 | Derlin-2 | Oda Y *et al*., 2006 |
|  | DERL3 | 91319 | Derlin-3 | Oda Y *et al*., 2006 |
|  | UBAC2 | 337867 | UBA domain containing 2 | Christianson *et al*., 2012; Sawalha *et al*., 2011 |
| **Mouse** | | | | |
|  | Parl | 381038 |  | Cipolat *et al*., 2006; Pagliarini *et al*., 2008; Ji *et al*., 2010 |
|  | Rhbdf1 | 13650 | Rhomboid family 1; iRhom1 | Christova *et al*., 2013; Maretzky *et al*., 2013 |
|  | Rhbdf2 | 217344 | Rhomboid 5 homolog 2; iRhom2 | Christova *et al*., 2013; Maretzky *et al*., 2013; Siggs *et al*., 2012 |
|  | Rhbdl1 | 117025 | Rhomboid; veinlet-like 1 | Lohi *et al*., 2004 |
|  | Rhbdl2 | 230726 | Rhomboid 2; veinlet-like 2; RRP2 | Urban and Freeman, 2003 |
|  | Rhbdl3 | 246104 | Rhomboid 3; veinlet-like 3; Rhbdl4; Rhomr1; Vrho | Jaszai and Brand, 2002 |
|  | Rhbdd1 | 76867 | Rhomboid domain containing 1; RHBDL4; RRP4 | Lemberg and Freeman, 2009; Wang *et al*., 2009 |
|  | Rhbdd2 | 215160 | Rhomboid domain containing 2; RHBDL7; Usmg1 | Lemberg and Freeman, 2009; Ahmedi *et al*., 2013 |
|  | Rhbdd3 | 279766 | Rhomboid domain containing 3 | Liu *et al*., 2013 |
|  | Derl1 | 67819 | Derlin-1 | Eura *et al*., 2012 |
|  | Derl2 | 116891 | Derlin-2 | Dougan *et al*., 2011 |
|  | Derl3 | 70377 | Derlin-3 | Eura *et al*., 2012 |
|  | Ubac2 | 68889 | Ubiquitin associated domain containing 2 |  |
| ***Arabidopsis*** | | | | |
|  | RBL1 | 817453 | At2g29050 | Kanaoka *et al*., 2005; Knopf *et al*., 2012 |
|  | RBL2 | 842616 | At1g63120 | Kanaoka *et al*., 2005; Knopf *et al*., 2012 |
|  | RBL3 | 830616 | At5g07250 | Kanaoka *et al*., 2005; Knopf *et al*., 2012; Koonin *et al*., 2003 |
|  | RBL4 | 824545 | At3g53780 | Kanaoka *et al*., 2005; Knopf *et al*., 2012 |
|  | RBL5 | 841690 | At1g52580 | Kanaoka *et al*., 2005; Knopf *et al*., 2012 |
|  | RBL6 | 837831 | At1g12750 | Kanaoka *et al*., 2005; Knopf *et al*., 2012 |
|  | RBL7 | 828406 | At4g23070 | Kanaoka *et al*., 2005 |
|  | RBL8 | 839113 | At1g25290; RBL10 | Garcia-Lorenzo *et* al., 2006;; Knopf *et al*., 2012 Koonin *et al*., 2003; Sedivy-Haley *et al*., 2012 |
|  | RBL9 | 832642 | At5g25752; RBL11 | Garcia-Lorenzo *et* al., 2006; Koonin *et al*., 2003; Knopf *et al*., 2012 |
|  | RBL10 | 821028 | At3g17611; RBL14 | Garcia-Lorenzo *et* al., 2006; Koonin *et al*., 2003 |
|  | RBL11 | 825015 | At3g58460; RBL15 | Garcia-Lorenzo *et* al., 2006; Koonin *et al*., 2003 |
|  | RBL12 | 825121 | At3g59520; RBL13 | Garcia-Lorenzo *et* al., 2006; Koonin *et al*., 2003; Kmiec-Wisniewska *et al*., 2008 |
|  | At1g18600 | 838441 | PARL; RBL12 | Garcia-Lorenzo *et* al., 2006; Knopf *et al*., 2012; Koonin *et al*., 2003 |
|  | At1g74130 | 843753 |  | Karakasis *et al*., 2007; Powles *et al*., 2013; Sedivy-Haley *et al*., 2011; Tripathi and Sowdhamini, 2006 |
|  | At1g74140 | 843754 |  | Tripathi and Sowdhamini, 2006 |
|  | At3g07950 | 819986 |  | Tripathi and Sowdhamini, 2006 |
|  | At5g25640 | 832640 |  |  |
|  | At5g38510 | 833839 | RBL9 | Garcia-Lorenzo *et* al., 2006; Tripathi and Sowdhamini, 2006 |
|  | At1g77860 | 844122 | KOMPEITO; KOM; RBL8 | Garcia-Lorenzo *et* al., 2006; Koonin *et al*., 2003 |
|  | DER1 | 829054 | At4g29330 | Lee *et al*., 2013 |
|  | DER2.1 | 828269 | At4g21810 | Lee *et al*., 2013 |
|  | DER2.2 | 825823 | At4g04860 | Lee *et al*., 2013 |
| ***Drosophila*** | | | | |
|  | Rho | 38168 | Rhomboid-1; veinlet; RHO1 | Sturtevand and Roark, 1993; Urban *et al*., 2001; Wasserman *et al*., 2000 |
|  | Stet | 38169 | Rho-2 | Urban *et al*., 2002 |
|  | Ru | 44856 | Roughoid; rho-3; ru-PA | Wasserman *et al*., 2000 |
|  | rho-4 | 32109 |  | Urban *et al*., 2002 |
|  | rho-5 | 2768944 | iRhom | Urban *et al*., 2002; Zettl *et al*., 2011 |
|  | rho-6 | 34640 |  | Urban *et al*., 2002 |
|  | rho-7 | 36281 | PARL | Urban *et al*., 2002 |
|  | DER-1 | 33369 |  | Lilley and Ploegh, 2004 |
|  | DER-2 | 42005 |  | Lilley and Ploegh, 2004 |
| ***C. elegans*** | | | | |
|  | ROM-1 | 184989 |  | Dutt *et al*., 2004 |
|  | ROM-2 | 183564 |  | Dutt *et al*., 2004 |
|  | ROM-3 | 191000 |  | Dutt *et al*., 2004 |
|  | ROM-4 | 3565076 |  | Dutt *et al*., 2004 |
|  | ROM-5 | 190260 |  | Dutt *et al*., 2004 |
| ***S. cerevisiae*** | | | | |
|  | RBD1 | 852993 | Pcp1; YGR101W | McQuibban *et al*., 2003 |
|  | RBD2 | 855830 | YPL246C | McQuibban *et al*., 2003 |
|  | DER1 | 852500 | Der1p; YBR201W | Knop *et al*., 1996; Zattas *et al*., 2013 |

**List of references cited in Table S2**

Adrain C, StrisovskyK, Zettl M, Hu L, Lemberg MK, Freeman M (2011) Mammalian EGF receptor activation by the rhomboid protease RHBDL2. EMBO Rep 12: 421-427.

Ahmedi NB, Gribanova Y, Njoku CC, Naidu A, Young A, Mendoza E, Yamashita CK, Ozgul RK, Johnsons JE, Fox DA, Farber DB (2013) Dynamics of the rhomboid-like protein RHBDD2 expression in mouse retina and involvement of its human ortholog in retinitis pigmentosa. J Biol Chem 288: 9742-9754.

Bahar A, Whitby P, Holley S, Hoban PR, Elder JB, Deakin M, Hall C, Clayton RN, Williams GT, Farrell WE (2007) Primary colorectal tumors fail to express the pro-apoptotic mediator PTAG and its re-expression augments drug-induced apoptosis. Genes Chromosomes Cancer 46: 202-212.

Blaydon DC, Etheridge SL, Risk JM, Hennies HC, Gay LJ, Carroll R, Plagnol V, McRonald FE, Stevens HP, Spurr NK, Bishop DT, Ellis A, Jankowshi J, Field JK, Leigh IM, South AP, Kelsell DP (2012) RHBDF2 mutations are associated with tylosis, a familial esophageal cancer syndrome. Am J Hum Genet 90: 340-346.

Canzoneri R, Lacunza E, Isla Larrain M, Croce MV, Abba MC (2014) Rhomboid family gene expression profiling in breast normal tissue and tumor samples. Tumour Biol 35(2): 1451-1458.

Chen SF, Wu CH, Lee YM, Tam K, Tsai YC, Liou JY, Shyue SK (2013) Caveolin-1 interacts with Derlin-1 and promotes ubiquitination and degradation of cyclooxygenase-2 via collaboration with p97 complex. J Biol Chem 288: 33462-33469.

Christianson JC, Olzmann JA, Shaler TA, Sowa ME, Bennett EJ, Richter CM, Tyler RE, Greenblatt EJ, Harper JW, Kopito RR (2012) Defining human ERAD networks through an integrative mapping strategy. Nature Cell Biol 14: 93-105.

Christova Y, Adrain C, Bambrough P, Ibrahim A, Freeman M (2013) Mammalian iRhoms have distinct physiological functions including an essential role in TACE regulation. EMBO Rep 14: 884-890.

Cipolat S, Rudka T, Hartmann D, Costa V, Serneels L, Craessaerts K, Metzger K, Frezza C, Annaert W, D'Adamio L, Derks C, Dejaegere T, Pellegrini L, D'Hooge R, Scorrano L, De Strooper B (2006) Mitochondrial rhomboid PARL regulates cytochrome c release during apoptosis via OPA1-dependent cristae remodeling. Cell 126: 163-175.

Curran JE, Jowett JB, Abraham LJ, Diepeveen LA, Elliot KS, Dyer TD, Kerr Bayles LJ, Johnson MP, Comuzzie AG, Moses EK, Walder KR, Collier GR, Blangero J, Kissebah AH (2010) Genetic variation in PARL influences mitochondrial content. Hum Genet 127: 183-190.

Dougan SK, Hu CC, Paguet ME, Greenblatt MB, Kim J, Lilley BN, Watson N, Ploegh HL (2011) Derlin-2-deficient mice reveal an essential role for protein dislocation in chondrocytes. Mol Cell Biol 31: 1145-1159.

Dutt A, Canevascini S, Froehli-Hoier E, Hajnal A (2004) EGF signal propagation during *C. elegans* vulval development mediated by ROM-1 rhomboid. PLoS One 2: e334.

Etheridge SL, Brooke MA, Kelsell DP, Blaydon DC (2013) Rhomboid proteins: a role in keratinocyte proliferation and cancer. Cell Tissue Res 351: 301-307.

Eura Y, Yanamoto H, Arai Y, Okuda T, Miyata T, Kokame K (2012) Derlin-1 deficiency is embryonic lethal, Derlin-3 deficiency appears normal, and Herp deficiency is intolerant to glucose load and ischemia in mice. PLoS One 7: e34298.

Garcia-Lorenzo M, Sjodin A, Jansson S, Funk C (2006) Protease gene families in *Populus* and *Arabidopsis*. BMC Plant Biol 6: 30.

Greenblatt EJ, Olzmann JA, Kopito RR (2011) Derlin-1 is a rhomboid pseudoprotease required for the dislocation of mutant α-1 antitrypsin from the endoplasmic reticulum. Nature Struct Mol Biol 18: 1147-1152.

Jaszai J, Brand M (2002) Cloning and expression of Ventrhoid, a novel vertebrate homologue of the *Drosophila* EGF pathway gene rhomboid. Mech Dev 113: 73-77.

Jin SM, Lazarou M, Wang C, Kane LA, Narendra DP, Youle RJ (2010) Mitochondrial membrane potential regulates PINK1 import and proteolytic destabilization by PARL. J Cell Biol 191: 933-942.

Kanaoka, M.M., S. Urban, M. Freeman and K. Okada K (2005) An *Arabidopsis* rhomboid homolog is an intramembrane protease in plants. FEBS Lett 579: 5723-5728.

Katiyar S, Joshi S, Lennarz WJ (2005) The retrotranslocation protein Derlin-1 binds peptide: *N*- glycanase to the endoplasmic reticulum. Mol Biol Cell 16: 4584-4594.

Knop M, Finger A, Braun T, Hellmuth K, Wolf DH (1996) Der1, a novel protein specifically required for endoplasmic reticulum degradation in yeast. EMBO J 15: 753-763.

Knopf RR, Feder A, Mayer K, Lin A, Rozenberg M, Schaller A, Adam Z (2012) Rhomboid proteins in the chloroplast envelope affect the level of allene oxide synthase in *Arabidopsis thaliana*. Plant J 72: 559-571.

Koonin EV, Makarova KS, Rogozin IB, Davidovic L, Letellier MC, Pellegrini L (2003) The rhomboids: a nearly ubiquitous family of intramembrane serine proteases that probably evolved by multiple ancient horizontal gene transfers. Genome Biol 4: R19.

Lacunza E, Canzoneri R, Rabassa ME, Zwenger A, Segal-Eras A, Croce MV, Abba MC (2012) RHBDD2: a 5-fluorouracil responsive gene over-expressed in the advanced stages of colorectal cancer. Tumour Biol 33: 2393-2399.

Lee H, Sparkes I, Gattolin S, Dzimitrowicz N, Roberts LM, Hawes C, Frigerio L (2013) An *Arabidopsis* reticulon and the atlastin homologue *RHD3-like 2* act together in shaping the tubular endoplasmic reticulum. New Phytol 197: 481-489.

Lei X, Li YM (2009) The processing of human rhomboid intramembrane serine protease RHBDL2 is required for its proteolytic activity. J Mol Biol 394: 815-825.

Lilley BN, Ploegh HL (2004) A membrane protein required for dislocation of mis-folded proteins from the ER. Nature 429: 834-840.

Liu J, Liu S, Xia M, Xu S, Wang C, Bao Y, Jiang M, Wu Y, Xu T, Cao X (2013) Rhomboid domain-containing protein 3 is a negative regulator of TLR3-triggered natural killer cell activation. Proc Natl Acad Sci USA 110: 7814-7819.

Liu XN, Tang ZH, Zhang Y, Pan QC, Chen XH, Yu YS, Zang GQ (2013) Lentivirus-mediated silencing of rhomboid domain containing 1 suppresses tumor growth and induces apoptosis in hepatoma HepG2 cells. Asian Pac J Cancer Prev. 14: 5-9.

Lohi O, Urban S, Freeman M (2004) Diverse substrate recognition mechanisms for rhomboids; thrombomodulin is cleaved by mammalian rhomboids. Curr Biol 14: 236-241.

Maretzky T, Mcllwain DR, Issuree PD, Li X, Malapeira J, Amin S, Lang PA, Mak TW, Blobel CP (2013) iRhom2 controls the substrate selectivity of stimulated ADAM17-dependent ectodomain shedding. Proc Natl Acad Sci USA 110: 11433-11438.

Meissner C, Lorenz H, Weihofen A, Selkoe DJ, Lemberg MK (2011) The mitochondrial intramembrane protease PARL cleaves human Pink1 to regulate Pink1 trafficking. J Neurochem 117: 856-867.

Nishitoh H, Kadowaki H, Nagai A, Maruyama T, Yokota T, Fukutomi H, Noguchi T, Matsuzawa A, Takeda K, Ichijo H (2008) ALS-linked mutant SOD1 induces ER stress- and ASK1-dependent motor neuron death by targeting Derlin-1. Genes and Dev 22: 1451-1464.

Oda Y, Okada T, Yoshida H, Kaufman RJ, Nagata K, Mori K (2006) Derlin-2 and Derlin-3 are regulated by the mammalian unfolded protein response and are required for ER-associated degradation. J Cell Biol 172: 383-393.

Pagliarini DJ, Calvo SE, Chang B, Sheth SA, Vafai SB, Ong SE, Walford GA, Sugiana C, Boneh A, Chen WK, Hill DE, Vidal M, Evans JG, Thorburn DR, Carr SA, Mootha VK (2008) A mitochondrial protein compendium elucidates complex I disease biology. Cell 134: 112-123.

Pascall JC, Brown KD (1998) Characterization of a mammalian cDNA encoding a protein with high sequence similarity to the *Drosophila* regulatory protein rhomboid. FEBS Lett 429: 337-340.

Pellegrini L, Passer BJ, Canelles M, Lefterov I, Ganjei JK, Fowlkes BJ, Koonin EV, D'Adamio L (2001) PAMP and PARL, two novel putative metalloproteases interacting with the COOH-terminus of Presenilin-1 and -2. J Alzheimers Dis 3: 181-190.

Saarinen S, Vahteristo P, Lehtonen R, Aittomäki K, Launonen V, Kiviluoto T, Aaltonen LA (2012) Analysis of a Finnish family confirms RHBDF2 mutations as the underlying factor in tylosis with esophageal cancer. Familial Cancer 11: 525-528

Sawalha AH, Hughes T, Nadig A, Yilmaz V, Aksu K, Keser G, Cefle A, Yazici A, Ergen A, Alarcon-Riquelme ME, SSalvarani D, Casali B, Direskeneli H, Saruhan-Direskeneli G (2011) A putative functional variant within the *UBAC2* gene is associated with increased risk of Behçet's disease. Arthritis and Rheumatism 63: 3607-3612.

Siggs OM, Xiao N, Wang Y, Shi H, Tomisato W, Li X, Xia Y, Beutler B (2012) iRhom2 is required for the secretion of mouse TNFα. Blood 119: 5769-5771.

Sturtevant MA, Roark M, Bier E (1993) The *Drosophila* rhomboid gene mediates the localized formation of wing veins and interacts genetically with components of the EGF-R signaling pathway. Genes and Dev 7: 961-973.

Sun F, Zhang R, Gong X, Geng X, Drain PF, Frizzell RA (2008) Derlin-1 Promotes the efficient degradation of the Cystic Fibrosis transmembrane conductance regulator (CFTR) and CFTR folding mutants. J Biol Chem 281: 36856-36863.

Tripathi LP, Sowdhamini R (2006) Cross genome comparisons of serine proteases in *Arabidopsis* and rice. BMC Genomics 7: 200.

Urban S, Lee JR, Freeman M (2002) A family of rhomboid intramembrane proteases activates all *Drosophila* membrane-tether EGF ligands. EMBO J 21: 4277-4286.

Wan C, Fu J, Wang Y, Miao S, Song W, Wang L (2012) Exosome-related multi-pass transmembrane protein TSAP6 is a target of rhomboid protease RHBDD1-induced proteolysis. PLoS One 7: e37452.

Wang B, Heath-Engel H, Zhang D, Nguyen N, Thomas DY, Hanrahan JW, Shore GC (2008) BAP31 interacts with Sec61 translocons and promotes retrotranslocation of CFTRDeltaF508 via the derlin-1 complex. Cell 133: 1080-1092.

Wang Y, Guan X, Fok KL, Li S, Zhang X, Miao S, Zong S, Koide SS, Chan HC, Wang L (2008) A novel member of the Rhomboid family, RHBDD1, regulates BIK-mediated apoptosis. Cell Mol Life Sci 65: 3822-3829.

Wang Y, Song W, Li S, Guan X, Miao S, Zong S, Koide SS, Wang L (2009) GC-1 mRHBDD1 knockdown spermatogonia cells lose their spermatogenic capacity in mouse seminiferous tubules. BMC Cell Biol 10: 25.

Wassernab JD, Urban S, Freeman M (2000) A family of rhomboid-like genes: *Drosophila* rhomboid-1 and roughoid/rhomboid-3 cooperate to activate EGF receptor signalling. Genes and Dev 14: 1651-1663.

Wojnarowicz PM, Provencher DM, Mes-Masson A, Tonin PN (2012) Chromosome 17q25 genes, RHBDF2 and CYGB, in ovarian cancer. Int J Oncol 40: 1865-1880.

Ye Y, Shibata Y, Yun C, Ron D, Rapoport TA (2004) A membrane protein complex mediates retro-translocation from the ER lumen into the cytosol. Nature 429: 841-847.

Zattas D, Adle DJ, Rubenstein EM, Hochstrasser M (2013) N-terminal acetylation of the yeast Derlin Der1 is essential for Hrd1 ubiquitin-ligase activity toward luminal ER substrates. Mol Biol Cell 24: 890-900.

Zettl M, Adrain C, Strisovsky K, Lastun V, Freeman M (2011) Rhomboid family pseudoproteases use the ER quality control machinery to regulate intercellular signalling. Cell 145: 79-91.
