# Supplementary material for: Alternative splice variants of rhomboid proteins: Comparative analysis of database entries for select model organisms and validation of functional potential
Source: F1000Res. 2018 May 31;7:139. Originally published 2018 Feb 1. [Version 2] doi: 10.12688/f1000research.13383.2 (PMC7065720; doi:10.12688/f1000research.13383.2)
Supplement: Supplementary file 7 [file f1000research-7-16431-s0007.tgz › cce5c9e8-3f8e-46f4-8069-4710eb69d9d2.zip!/Table S3.docx]

**Table S3: Summary of alternative splice variants impacting the 5’ UTR and translation.**

Details are provided for splice variants that impact the indicated region of the rhomboid transcript and/or protein. For each entry, the columns provide information concerning its accession number, model species, rhomboid type, and a summary of the effect of the alternative splicing. The same format is used for all of the following tables (S4 to S10).

| **Translational (5' UTR)** | |  |  |
| --- | --- | --- | --- |
| **Rhomboid Name (accession #) [total forms]** | **Species** | **Rhomboid Type** | **Effects of Splicing on the UTR sequence and/or How the Alternate Start Site Was Created** |
| X5 PARL (XM_017006803.1 / XP_016862292.1) [10 forms] | Human | PARL | Removal of the 5' UTR that resulted in an alternate start site downstream |
|  |  |  |  |
| X1 RHBDF1 iRhom1 (XM_005255494.1 / XP_005255551.1) [7 forms] | Human | iRhom (evolved from PARL) | Sequence added upstream to the 5’ UTR by replacing the beginning of the default 5’ UTR |
| X2 RHBDF1 iRhom1 (XM_017023556.1 / XP_016879045.1) [7 forms] | Human | iRhom (evolved from PARL) | Sequence added upstream to the 5’ UTR by replacing the beginning of the default 5’ UTR |
| X3 RHBDF1 iRhom1 (XM_006720921.1 / XP_006720984.1) [7 forms] | Human | iRhom (evolved from PARL) | Most of the 5’ UTR was removed |
| X5 RHBDF1 iRhom1 (XM_005255498.2 / XP_005255555.1) [7 forms] | Human | iRhom (evolved from PARL) | 5’ UTR was removed that resulted in an alternate start site downstream |
| X6 RHBDF1 iRhom1 (XM_017023558.1 / XP_016879047.1) [7 forms] | Human | iRhom (evolved from PARL) | Most of the 5’ UTR was removed |
|  |  |  |  |
| Isoform2 RHBDF2 iRhom2 (NM_001005498.3 / XP_001005498.2) [8 forms] | Human | iRhom (evolved from PARL) | Sequence added downstream the isoform 1 5’UTR |
| X1 RHBDF2 iRhom2 (XM_0011525250.2 / XP_011523552.1) [8 forms] | Human | iRhom (evolved from PARL) | Sequence added to the beginning of the 5' UTR |
| X2 RHBDF2 iRhom2 (XM_011525251.2 / XP_011523553.1) [8 forms] | Human | iRhom (evolved from PARL) | Sequence removed from the beginning of the 5' UTR |
| X3 RHBDF2 iRhom2 (XM_005257670.1 / XP_005257727.1) [8 forms] | Human | iRhom (evolved from PARL) | Sequence removed from the beginning of the 5' UTR |
| X4 RHBDF2 iRhom2 (XM_017025079.1 / XP_016880568.1) [8 forms] | Human | iRhom (evolved from PARL) | Removal of the default 5’UTR that resulted in an extension downstream from isoform 1 5’ UTR |
| X5 RHBDF2 iRhom2 (XM_005257669.3 / XP_005257726.1) [8 forms] | Human | iRhom (evolved from PARL) | Removal of the default 5’UTR that resulted in an extension downstream from isoform 1 5’UTR |
| X6 RHBDF2 iRhom2 (XM_011525249.2 / XP_011523551.1) [8 forms] | Human | iRhom (evolved from PARL) | Unique and longer 5’ UTR created relative to the isoform 1 5’UTR |
|  |  |  |  |
| Isoform 3 RHBDL1 (XM_005255665.1 / XP_005255722.1) [5 forms] | Human | Secretase-type (6+1) | Slightly shortened 5'UTR by removing some base pairs at the beginning of the 5’ UTR, resulting in an alternate start site |
| X1 RHBDL1 (XM_017023849.1 / XP_016879338.1) [5 forms] | Human | Secretase-type (6+1) | Created a different sequence for the 5' UTR and beginning coding region, resulting in an alternate start site |
| X2 RHBDL1 (XM_017023850.1 / XP_016879339.1) [5 forms] | Human | Secretase-type (6+1) | Created a different sequence for the 5' UTR and beginning coding region, resulting in an alternate start site |
|  |  |  |  |
| Isoform 2 RHBDL2 (XM_017821.4 / NP_060291.2) [2 forms] | Human | Secretase-type (6+1) | Created a unique and extended 5’UTR sequence, resulting in an alternate start site |
|  |  |  |  |
| X7 RHBDL3 (XM_017024279.1 / XP_016879768.1) [13 forms] | Human | Secretase-type (6+1) | Created a unique 5”UTR sequence longer than the isoform 1 5’UTR that resulted in an alternate start site |
| X8 RHBDL3 (XM_006721734.3 / XP_006721797.1) [13 forms] | Human | Secretase-type (6+1) | Created a unique 5”UTR sequence longer than the isoform 1 5’UTR that resulted in an alternate start site |
| X9 RHBDL3 (XM_017024274.1 / XP_016879763.1) [13 forms] | Human | Secretase-type (6+1) | Created a unique 5”UTR sequence longer than the isoform 1 5’UTR that resulted in an alternate start site |
| X10 RHBDL3 (XM_017024278.1 / XP_016879767.1) [13 forms] | Human | Secretase-type (6+1) | Created a unique 5”UTR sequence longer than the isoform 1 5’UTR that resulted in an alternate start site |
| X11 RHBDL3 (XM_017024277.1 / XP_016879766.1) [13 forms] | Human | Secretase-type (6+1) | Created a unique 5”UTR sequence longer than the isoform 1 5’UTR that resulted in an alternate start site |
| X12 RHBDL3 (XM_017024280.1 / XP_016879769.1) [13 forms] | Human | Secretase-type (6+1) | Incorporated part of the ORF from isoform 1 into the 5’UTR that resulted in an alternate start site. |
|  |  |  |  |
| Isoform2 RHBDD1 (NM_001167608.1 / NP_001161080.1) [15 forms] | Human | Secretase-type (basic) | Created a unique 5’UTR sequence |
| X1 RHBDD1 (XM_017005089.1 / XP_016860578.1) [15 forms] | Human | Secretase-type (basic) | Created a unique 5’UTR sequence |
| X2 RHBDD1 (XM_017005091.1 / XP_016860580.1) [15 forms] | Human | Secretase-type (basic) | Created a unique 5’UTR sequence |
| X3 RHBDD1 (XM_017005094.1 / XP_016860583.1) [15 forms] | Human | Secretase-type (basic) | Created a unique 5’UTR sequence |
| X4 RHBDD1 (XM_017005085.1 / XP_016860574.1) [15 forms] | Human | Secretase-type (basic) | Created a unique 5’UTR sequence |
| X5 RHBDD1 (XM_017005087.1 / XP_016860576.1) [15 forms] | Human | Secretase-type (basic) | Created a unique 5’UTR sequence |
| X6 RHBDD1 (XM_017005084.1 / XP_016860573.1) [15 forms] | Human | Secretase-type (basic) | Created a unique 5’UTR sequence |
| X7 RHBDD1 (XM_017005090.1 / XP_016860579.1) [15 forms] | Human | Secretase-type (basic) | Created a unique 5’UTR sequence |
| X8 RHBDD1 (XM_017005086.1 / XP_016860575.1) [15 forms] | Human | Secretase-type (basic) | Created a unique 5’UTR sequence |
| X9 RHBDD1 (XM_017005092.1 / XP_016860581.1) [15 forms] | Human | Secretase-type (basic) | Created a unique 5’UTR sequence |
| X10 RHBDD1 (XM_017005083.1 / XP_016860572.1) [15 forms] | Human | Secretase-type (basic) | Created a unique 5’UTR sequence |
| X11 RHBDD1 (XM_017005088.1 / XP_016860577.1) [15 forms] | Human | Secretase-type (basic) | Created a unique 5’UTR sequence |
| X12 RHBDD1 (XM_017005093.1 / XP_016860582.1) [15 forms] | Human | Secretase-type (basic) | Created a unique 5’UTR sequence |
| X13 RHBDD1 (XM_017005095.1 / XP_016860584.1) [15 forms] | Human | Secretase-type (basic) | Created a unique 5’UTR sequence |
|  |  |  |  |
| IsoformB RHBDD2 (NM_001040457.2 / NP_001035547.1) [6 Forms] | Human | distant relation | Created an extension of the 5’UTR with a coding region sequence and an extra segment. As a result. An alternate start site was created. |
| X1 RHBDD2 (XM_005250511.4 / XP_005250568.1) [6 Forms] | Human | distant relation | Created an extension of the 5' UTR with a coding region sequence plus an extra segment |
|  |  |  |  |
| X1 RHBDD3 (XM_017028750.1 / XP_016884239.1) [5 forms] | Human | distant relation | Extended 5’ UTR with a unique sequence that resulted in an alternate start site |
| X2 RHBDD3 (XM_006724224.3 / XP_006724287.1) [5 forms] | Human | distant relation | Extended and unique sequence |
| X3 RHBDD3 (XM_017028749.1 / XP_016884238.1) [5 forms] | Human | distant relation | Extended 5’ UTR with a unique sequence that resulted in an alternate start site |
| X4 RHBDD3 (XM_011530107.2 / XP_011528409.1) [5 forms] | Human | distant relation | Extended 5’ UTR with a unique sequence |
|  |  |  |  |
| Isoform c DERL1 (NM_001330601.1/NP_001317530.1) [4 forms] | Human | Rhomboid pseudoprotease | Extended 5’ UTR with a unique sequence that resulted in an alternate start site |
| X2 DERL1 (XM_006716657.1/XP_006716720.1) [4 forms] | Human | Rhomboid pseudoprotease | Extended 5’ UTR with a unique sequence that resulted in an alternate start site |
|  |  |  |  |
| X1 DERL3 (XM_011530506.2/XP_011528808.1) [10 forms] | Human | Rhomboid pseudoprotease | Extended 5’ UTR with a unique sequence |
| X2 DERL3 (XM_017029082.1/XP_016884571.1) [10 forms] | Human | Rhomboid pseudoprotease | Extended 5’ UTR with a unique sequence that resulted in an alternate start site |
| X3 DERL3 (XM_017029080.1/XP_016884569.1) [10 forms] | Human | Rhomboid pseudoprotease | Extended 5’ UTR with a unique sequence |
| X4 DERL3 (XM_017029079.1/XP_016884568.1) [10 forms] | Human | Rhomboid pseudoprotease | Extended 5’ UTR with a unique sequence |
| X5 DERL3 (XM_017029078.1/XP_016884567.1) [10 forms] | Human | Rhomboid pseudoprotease | Extended 5’ UTR with a unique sequence |
| X6 DERL3 (XM_011530505.2/XP_011528807.1) [10 forms] | Human | Rhomboid pseudoprotease | Extended 5’ UTR with a unique sequence |
| X7 DERL3 (XM_017029081.1/XP_016884570.1) [10 forms] | Human | Rhomboid pseudoprotease | Extended 5’ UTR with a unique sequence |
|  |  |  |  |
| Isoform 2 UBAC2 (NM_177967.3/NP_808882.1) [7 forms] | Human | Rhomboid pseudoprotease | Created a different 5’UTR sequence and an alternate start site |
| X1 UBAC2 (XM_011521082.2/XP_011519384.1) [7 forms] | Human | Rhomboid pseudoprotease | Created a shorter 5’ UTR sequence with an alternate start site |
| X2 UBAC2 (XM_006719948.3/XP_006720011.1) [7 forms] | Human | Rhomboid pseudoprotease | Created a different 5’UTR sequence and an alternate start site |
| X3 UBAC2 (XM_011521083.2/XP_011519385.1) [7 forms] | Human | Rhomboid pseudoprotease | Created a different 5’UTR sequence and an alternate start site |
| X4 UBAC2 (XM_011521084.2/XP_011519386.1) [7 forms] | Human | Rhomboid pseudoprotease | Created a different shorter 5’UTR sequence and an alternate start site |
|  |  |  |  |
| X1 PARL (XM_006522312.1/XP_006522375.1) [2 forms] | Mouse | PARL | Created a slightly longer 5' UTR upstream that resulted in part of the original 5’ UTR becoming coding region and hence an alternate start site |
|  |  |  |  |
| X1 Rhbdf1 iRhom1 (XM_006514492.1/XP_006514555.1) [13 forms] | Mouse | iRhom (evolved from PARL) | Created a significantly longer 5' UTR from previously spliced out nucleotides, plus a short deletion in the middle and hence creating an alternate start site |
| X2 Rhbdf1 iRhom1 (XM_006514493.1/XP_006514556.1) [13 forms] | Mouse | iRhom (evolved from PARL) | Created a significantly longer 5' UTR from previously spliced out nucleotides, plus a short deletion in the middle and hence creating an alternate start site |
| X3 Rhbdf1 iRhom1 (XM_006514494.1/XP_006514557.1) [13 forms] | Mouse | iRhom (evolved from PARL) | Created a short extension to the beginning of the 5' UTR |
| X4 Rhbdf1 iRhom1 (XM_006514495.1/XP_006514558.1) [13 forms] | Mouse | iRhom (evolved from PARL) | Created a short extension to the beginning of the 5' UTR, plus another insertion in the middle of the 5’UTR |
| X5 Rhbdf1 iRhom1 (XM_006514496.1/XP_006514559.1) [13 forms] | Mouse | iRhom (evolved from PARL) | Created a short extension to the beginning of the 5' UTR using previously spliced out nucleotides |
| X6 Rhbdf1 iRhom1 (XM_006514497.1/XP_006514560.1) [13 forms] | Mouse | iRhom (evolved from PARL) | Created a short extension to the beginning of the 5' UTR using previously spliced out nucleotides, plus a short deletion in the middle and hence resulting in an alternate start site |
| X7 Rhbdf1 iRhom1 (XM_006514498.1/XP_006514561.1) [13 forms] | Mouse | iRhom (evolved from PARL) | Created a short extension to the beginning of the 5' UTR using previously spliced out nucleotides, plus a short deletion in the middle and hence resulting in an alternate start site |
| X8 Rhbdf1 iRhom1 (XM_006514499.1/XP_006514562.1) [13 forms] | Mouse | iRhom (evolved from PARL) | Created a short extension to the beginning of the 5' UTR |
| X9 Rhbdf1 iRhom1 (XM_006514500.1/XP_006514563.1) [13 forms] | Mouse | iRhom (evolved from PARL) | Created a short extension to the beginning of the 5' UTR using previously spliced out nucleotides, plus a short deletion in the middle and hence resulting in an alternate start site |
| X10 Rhbdf1 iRhom1 (XM_006514501.1/XP_006514564.1) [13 forms] | Mouse | iRhom (evolved from PARL) | Created a short extension to the beginning of the 5' plus inclusion of coding region into the UTR and hence resulting in an alternate start site |
| X11 Rhbdf1 iRhom1 (XM_006514502.1/XP_006514565.1) [13 forms] | Mouse | iRhom (evolved from PARL) | Created a short extension to the beginning of the 5' plus inclusion of coding region into the UTR and hence resulting in an alternate start site |
| X12 Rhbdf1 iRhom1 (XM_006514503.1/XP_006514566.1) [13 forms] | Mouse | iRhom (evolved from PARL) | Created an out of frame sequence and subsequently converting previous coding region into a 5' UTR with an alternate start site |
|  |  |  |  |
| Isoform2 Rhbdf2 (NM_001167680.1/NP_001161152.1) [3 forms] | Mouse | iRhom (evolved from PARL) | Created an upstream extension to the 5' UTR |
| X1 Rhbdf2 (XM_006533108.1/XP_006533171.1) [3 forms] | Mouse | iRhom (evolved from PARL) | Created a short upstream extension to the 5' UTR |
|  |  |  |  |
| X1 Rhbdl1 (XM_006524021.1/XP_006524084.1) [3 forms] | Mouse | Secretase-type (6+1) | Created an extension to the beginning of the 5' UTR using previously spliced out nucleotides, resulting in an alternate start site |
| X2 Rhbdl2 (XM_006524022.1/XP_006524085.1) [3 forms] | Mouse | Secretase-type (6+1) | Created an extension to the beginning of the 5' UTR using previously spliced out nucleotides, resulting in an alternate start site |
|  |  |  |  |
| X1 Rhbdl2 (XM_006503025.1/XP_006503088.1) [3 forms] | Mouse | Secretase-type (6+1) | Created an equal length, but different 5' UTR generated from the downstream nucleotide sequence that was previously spliced out |
| X2 Rhbdl2 (XM_006503026.1/XP_006503089.1) [3 forms] | Mouse | Secretase-type (6+1) | Created a 5' UTR from what used to be a coding region with an insertion of nucleotides from what used to be spliced out |
|  |  |  |  |
| X1 Rhbdl3 (XM_006533326.1/XP_006533389.1) [7 forms] | Mouse | Secretase-type (6+1) | Created an upstream extension to the 5’ UTR |
| X2 Rhbdl3 (XM_006533327.1/XP_006533390.1) [7 forms] | Mouse | Secretase-type (6+1) | Created an upstream extension to the 5’ UTR |
| X3 Rhbdl3 (XM_006533328.1/XP_006533391.1) [7 forms] | Mouse | Secretase-type (6+1) | Created an upstream extension to the 5’ UTR |
| X4 Rhbdl3 (XM_006533329.1/XP_006533392.1) [7 forms] | Mouse | Secretase-type (6+1) | Created an upstream extension to the 5’ UTR |
| X5 Rhbdl3 (XM_006533330.1/XP_006533393.1) [7 forms] | Mouse | Secretase-type (6+1) | Created an upstream extension to the 5’ UTR |
| X6 Rhbdl3 (XM_006533331.1/XP_006533394.1) [7 forms] | Mouse | Secretase-type (6+1) | Created an upstream extension to the 5’ UTR |
|  |  |  |  |
| Isoform2 Rhbdd1 (NM_001122685.1/NP_001116157.1) [3 forms] | Mouse | Secretase-type (basic) | Created a longer UTR using previously spliced out nucleotides in the default form |
|  |  |  |  |
| X1 Rhbdd2 (XM_006504416.1/XP_006504479.1) [3 forms] | Mouse | distant relation | Created a longer 5' UTR with the first two coding exons, plus an insertion from normally spliced out nucleotides and hence resulting in an alternate start site |
| X2 Rhbdd2 (XM_006504417.1/XP_006504480.1) [3 forms] | Mouse | distant relation | Created a new 5’UTR that contained the beginning sequence of the 5' UTR, a short sequence from previously spliced out nucleotide, and the second exon. This combination of sequences in the 5’UTR resulted in an alternate start site. |
|  |  |  |  |
| X1 Rhbdd3 (XM_006514713.1/XP_006514776.1) [6 forms] | Mouse | inactive distant relation | Created a short extension to the 5' UTR |
| X2 Rhbdd3 (XM_006514714.1/XP_006514777.1) [6 forms] | Mouse | inactive distant relation | Created a shorter 5' UTR (primarily at the beginning sequence) |
| X3 Rhbdd3 (XM_006514715.1/XP_006514778.1) [6 forms] | Mouse | inactive distant relation | Created a deletion of the beginning 5’ UTR sequence plus insertion of a short sequence from normally spliced out nucleotides |
| X4 Rhbdd3 (XM_006514716.1/XP_006514779.1) [6 forms] | Mouse | inactive distant relation | Created a deletion of the beginning 5’ UTR sequence plus insertion of a short sequence from normally spliced out nucleotides |
|  |  |  |  |
| RBL6 At1g12750 (NM_001084059.2/NP_001077528.1) [3 forms] | Arabidopsis | Secretase type (6+1) | Created a longer 5' UTR by extending the middle |
| RBL6 At1g12750 (NM_001198046.1/NP_001184975.1) [3 forms] | Arabidopsis | Secretase type (6+1) | Created a longer 5' UTR by extending the middle |
|  |  |  |  |
| At1g74130 (NM_202413.1/NP_974141.1) [4 forms] | Arabidopsis | Inactive homologue | Created a shorter 5' UTR of less than half the original length |
|  |  |  |  |
| At1g74140 (NM_001084351.2/NP_001077820.1) [5 forms] | Arabidopsis | Inactive homologue | Created a very short 5' UTR |
|  |  |  |  |
| RBL 14 At3g17611 (NM_202600.1/NP_974329.1) [3 forms] | Arabidopsis | Secretase (basic) | Created a longer 5' UTR by including coding nucleotides and resulting in an alternate start site |
| RBL 14 At3g17611 (NM_001084701.1/NP_001078170.1) [3 forms] | Arabidopsis | Secretase (basic) | Created an extension of the 5’ UTR by including exon1 and half of exon2 and resulting in an alternate start site |
|  |  |  |  |
| RBL4 At3g53780 (NM_115238.2/NP566989.1) [2 forms] | Arabidopsis | Secretase type (6+1) | Created an extension of the 5’ UTR by including Exon1 and half of exon2 |
|  |  |  |  |
| RBL15 At3g58460 (NM_001203197.1/NP_001190126.1) [2 forms] | Arabidopsis | Secretase (basic) | Created a shorter 5' UTR using nucleotides upstream from the original UTR and resulting in an alternate start site |
|  |  |  |  |
| At5g38510 (NM_001085212.1/NP_001078681.1) [2 forms] | Arabidopsis | Inactive homologue | Created a shorter 5' UTR using downstream nucleotides normally spliced out |
