# Supplementary material for: Alternative splice variants of rhomboid proteins: Comparative analysis of database entries for select model organisms and validation of functional potential
Source: F1000Res. 2018 May 31;7:139. Originally published 2018 Feb 1. [Version 2] doi: 10.12688/f1000research.13383.2 (PMC7065720; doi:10.12688/f1000research.13383.2)
Supplement: Supplementary file 7 [file f1000research-7-16431-s0007.tgz › cce5c9e8-3f8e-46f4-8069-4710eb69d9d2.zip!/Table S4.docx]

**Table S4: Summary of alternative splice variants impacting the amino terminal sequences.**

| **Amino End** | |  |  |
| --- | --- | --- | --- |
| **Rhomboid Name (accession #) [total forms]** | **Species** | **Rhomboid Type** | **Effect of Splicing** |
| X5 PARL (XM_017006803.1/XP_005247643.1) [10 forms] | Human | PARL | Whole amino end is missing, leading to an alternate starting methionine |
|  |  |  |  |
| X2 RHBDF1 iRhom1 (XM_017023556.1/XP_016879045.1) [7 forms] | Human | iRhom (evolved from PARL) | Additional residues added after amino acid number 38 |
| X3 RHBDF1 iRhom1 (XM_006720921.1/XP_006720984.1) [7 forms] | Human | iRhom (evolved from PARL) | Additional residues added after amino acid number 38 |
| X4 RHBDF1 iRhom1 (XM_017023557.1/XP_016879046.1) [7 forms] | Human | iRhom (evolved from PARL) | Additional residues added after amino acid number 38 |
| X5 RHBDF1 iRhom1 (XM_005255498.2/XP_005255555.1) [7 forms] | Human | iRhom (evolved from PARL) | Amino terminus not present, leading to an alternate starting methionine |
| X6 RHBDF1 iRhom1 (XM_017023558.1/XP_016879047.1) [7 forms] | Human | iRhom (evolved from PARL) | Additional residues added after amino acid number 38 |
|  |  |  |  |
| Isoform2 RHBDF2 iRhom2 (NM_001005498.3/XP_001005498.2) [8 forms] | Human | iRhom (evolved from PARL) | 29 residues removed after amino acid number 49 |
| X1 RHBDF2 iRhom2 (XM_011525250.2/XP_011523552.1) [8 forms] | Human | iRhom (evolved from PARL) | 29 residues removed after amino acid number 49 |
| X2 RHBDF2 iRhom2 (XM_011525251.2/XP_011523553.1) [8 forms] | Human | iRhom (evolved from PARL) | 29 residues removed after amino acid number 49 |
| X3 RHBDF2 iRhom2 (XM_005257670.1/XP_005257727.1) [8 forms] | Human | iRhom (evolved from PARL) | 29 residues removed after amino acid number 49 |
| X4 RHBDF2 iRhom2 (XM_017025079.1/XP_016880568.1) [8 forms] | Human | iRhom (evolved from PARL) | 29 residues removed after amino acid number 49 |
| X5 RHBDF2 iRhom2 (XM_005257669.3/XP_005257726.1) [8 forms] | Human | iRhom (evolved from PARL) | 29 residues removed after amino acid number 49 |
| X6 RHBDF2 iRhom2 (XM_011525249.2/XP_011523551.1) [8 forms] | Human | iRhom (evolved from PARL) | 29 residues removed after amino acid number 49 |
|  |  |  |  |
| Isoform3 RHBDL1 (NM_001318733.1/NP_001305662.1) [5 forms] | Human | Secretase-type (6+1) | Alternate starting methionine that resulted in the introduction of 65 additional residues |
| X1 RHBDL1 (XM_017023849.1/XP_016879338.1) [5 forms] | Human | Secretase-type (6+1) | Alternate starting methionine that resulted in the introduction of 65 additional residues |
| X2 RHBDL1 (XM_017023850.1/XP_016879339.1) [5 forms] | Human | Secretase-type (6+1) | Alternate starting methionine that resulted in the introduction of 65 additional residues |
|  |  |  |  |
| Isoform 2 RHBDL2 (NM_017821.4/NP_060291.2 [2 forms] | Human | Secretase-type (6+1) | Alternate starting methionine that resulted in the removal of 80 residues |
|  |  |  |  |
| Isoform 2 RHBDL3 (NM_001330181.1/NP_001317110.1) [13 forms] | Human | Secretase-type (6+1) | Removal of 8 residues after amino acid number 37 |
| X2 RHBDL3 (XM_017024275.1/XP_016879764.1) [13 forms] | Human | Secretase-type (6+1) | Removal of 53 amino acids after the first 45 residues |
| X3 RHBDL3 (XM_017024273.1/XP_016879762.1) [13 forms] | Human | Secretase-type (6+1) | Removal of 8 residues after amino acid number 37 |
| X4 RHBDL3 (XM_017024276.1/XP_016879765.1) [13 forms] | Human | Secretase-type (6+1) | Removal of 61 amino acids after the first 37 residues |
| X6 RHBDL3 (XM_011524443.2/XP_011522745.1) [13 forms] | Human | Secretase-type (6+1) | Removal of 61 amino acids after the first 37 residues |
| X7 RHBDL3 (XM_017024279.1/XP_016879768.1) [13 forms] | Human | Secretase-type (6+1) | Alternate starting methionine that resulted in the removal of the first 98 residues |
| X8 RHBDL3 (XM_006721734.3/XP_006721797.1) [13 forms] | Human | Secretase-type (6+1) | Alternate starting methionine that resulted in the removal of first 42 residues |
| X9 RHBDL3 (XM_017024274.1/XP_016879763.1) [13 forms] | Human | Secretase-type (6+1) | Alternate starting methionine that resulted in the removal of first 42 residues |
| X10 RHBDL3 (XM_017024278.1/XP_016879767.1) [13 forms] | Human | Secretase-type (6+1) | Alternate starting methionine that resulted in the removal of first 98 residues |
| X11 RHBDL3 (XM_017024277.1/XP_016879766.1) [13 forms] | Human | Secretase-type (6+1) | Alternate starting methionine that resulted in the removal of first 98 residues |
| X12 RHBDL3 (XM_017024280.1/XP_016879769.1) [13 forms] | Human | Secretase-type (6+1) | Alternate starting methionine downstream resulting in the loss of the default amino end |
|  |  |  |  |
| Isoform b RHBDD2 (NM_001040457.2/NP_001035547.1) [6 Forms] | Human | distant relation | Alternate starting methionine that resulted in the removal of first 141 residues |
| Isoform b RHBDD2 (NM_001346186.1/NP_001333115.1) [6 Forms] | Human | distant relation | Alternate starting methionine that resulted in the removal of first 141 residues |
| Isoform b RHBDD2 (NM_001346187.1/NP_001333116.1) [6 Forms] | Human | distant relation | Alternate starting methionine that resulted in the removal of first 141 residues |
| Isoform c RHBDD2 (NM_001346188.1/NP_001333117.1) [6 Forms] | Human | distant relation | Removal of amino terminal sequence after the first 60 residues |
| Isoform c RHBDD2 (NM_001346189.1/NP_001333118.1) [6 Forms] | Human | distant relation | Removal of amino terminal sequence after the first 60 residues |
|  |  |  |  |
| X1 RHBDD3 (XM_017028750.1/XP_016884239.1) [5 forms] | Human | inactive distant relation | Alternate starting methionine that resulted in the removal of the first 143 residues |
| X2 RHBDD3 (XM_006724224.3/XP_006724287.1) [5 forms] | Human | inactive distant relation | Changes and removal of amino acids after the first 49 residues |
| X3 RHBDD3 (XM_017028749.1/XP_016884238.1) [5 forms] | Human | inactive distant relation | Alternate starting methionine that resulted in a unique sequence |
|  |  |  |  |
| Isoform c DERL1 (NM_001330601.1/NP_001317530.1) [4 forms] | Human | Rhomboid pseudoprotease | Alternate starting methionine that resulted in the removal of the first 100 residues |
| X2 DERL1 (XM_006716657.1/XP_006716720.1) [4 forms] | Human | Rhomboid pseudoprotease | Alternate starting methionine that resulted in the removal of the first 100 residues |
|  |  |  |  |
| Isoform b DERL2 (NM_001304777.1/NP_001291706.1) [3 forms] | Human | Rhomboid pseudoprotease | Sequence changes after residue number 30 |
| Isoform c DERL2 (NM_001304779.1/NP_0012991708.1) [3 forms] | Human | Rhomboid pseudoprotease | Sequence changes after residue number 53 |
|  |  |  |  |
| X2 DERL3 (XM_017029082.1/XP_016884571.1) [10 forms] | Human | Rhomboid pseudoprotease | Alternate starting methionine that resulted in the removal of the first 74 residues |
| X3 DERL3 (XM_017029080.1/XP_016884569.1) [10 forms] | Human | Rhomboid pseudoprotease | Deletion of 20 residues between the first residues 30 and 50 |
| X4 DERL3 (XM_017029079.1/XP_016884568.1) [10 forms] | Human | Rhomboid pseudoprotease | Changes in 2 amino acids between residues 30 and 33 |
| X6 DERL3 (XM_011530505.2/XP_011528807.1) [10 forms] | Human | Rhomboid pseudoprotease | Changes in 2 amino acids between residues 30 and 33 |
|  |  |  |  |
| Isoform 2 UBAC2 (NM_177967.3/NP_808882.1) [7 forms] | Human | Rhomboid pseudoprotease | Alternate starting methionine that introduced a unique sequence before residue number 130 |
| X1 UBAC2 (XM_011521082.2/XP_011519384.1) [7 forms] | Human | Rhomboid pseudoprotease | Alternate starting methionine that introduced a unique sequence before residue number 130 |
| X2 UBAC2 (XM_006719948.3/XP_006720011.1) [7 forms] | Human | Rhomboid pseudoprotease | Alternate starting methionine that resulted in the removal of the first 113 residues |
| X3 UBAC2 (XM_011521083.2/XP_011519385.1) [7 forms] | Human | Rhomboid pseudoprotease | Alternate starting methionine that introduced a unique sequence before residue number 130 |
| X4 UBAC2 (XM_011521084.2/XP_011519386.1) [7 forms] | Human | Rhomboid pseudoprotease | Alternate starting methionine that resulted in the removal of the first 113 residues |
| X5 UBAC2 (XM_017020553.1/XP_016876042.1) [7 forms] | Human | Rhomboid pseudoprotease | Alternate starting methionine that resulted in the removal of the first 150 residues |
|  |  |  |  |
| X1 PARL (XM_006522312.1/XP_006522375.1) [2 forms] | Mouse | PARL | The first 164 residues are replaced by a unique 43 residue sequence |
|  |  |  |  |
| X1 Rhbdf1 iRhom1 (XM_006514492.1/XP_006514555.1) [13 forms] | Mouse | iRhom (evolved from PARL) | Alternate starting methionine resulted in an extension to the amino terminus by 13 residues and an insertion of 35 residues after amino acid number 82 |
| X2 Rhbdf1 iRhom1 (XM_006514493.1/XP_006514556.1) [13 forms] | Mouse | iRhom (evolved from PARL) | Alternate starting methionine resulted in an extension to the amino terminus by 13 residues and an insertion of 35 residues after amino acid number 82 |
| X3 Rhbdf1 iRhom1 (XM_006514494.1/XP_006514557.1) [13 forms] | Mouse | iRhom (evolved from PARL) | Insertion of 35 residues after amino acid number 82 |
| X4 Rhbdf1 iRhom1 (XM_006514495.1/XP_006514558.1) [13 forms] | Mouse | iRhom (evolved from PARL) | Insertion of 35 residues after amino acid number 82 |
| X5 Rhbdf1 iRhom1 (XM_006514496.1/XP_006514559.1) [13 forms] | Mouse | iRhom (evolved from PARL) | Insertion of 35 residues after amino acid number 82 |
| X6 Rhbdf1 iRhom1 (XM_006514497.1/XP_006514560.1) [13 forms] | Mouse | iRhom (evolved from PARL) | Alternate starting methionine resulted in an extension to the amino terminus by 13 residues and an insertion of 35 residues after amino acid number 82 |
| X7 Rhbdf1 iRhom1 (XM_006514498.1/XP_006514561.1) [13 forms] | Mouse | iRhom (evolved from PARL) | Alternate starting methionine that resulted in a 13 residue extension of the amino terminus |
| X9 Rhbdf1 iRhom1 (XM_006514500.1/XP_006514563.1) [13 forms] | Mouse | iRhom (evolved from PARL) | Alternate starting methionine that resulted in a 13 residue extension of the amino terminus |
| X10 Rhbdf1 iRhom1 (XM_006514501.1/XP_006514564.1) [13 forms] | Mouse | iRhom (evolved from PARL) | Alternate starting methionine that resulted in the removal of 213 residues |
| X11 Rhbdf1 iRhom1 (XM_006514502.1/XP_006514565.1) [13 forms] | Mouse | iRhom (evolved from PARL) | Alternate starting methionine that resulted in the removal of 213 residues |
| X12 Rhbdf1 iRhom1 (XM_006514503.1/XP_006514566.1) [13 forms] | Mouse | iRhom (evolved from PARL) | Alternate starting methionine that resulted in the removal of the amino end |
|  |  |  |  |
| X1 Rhbdl1 (XM_006524021.1/XP_006524084.1) [3 forms] | Mouse | Secretase-type (6+1) | Alternate starting methionine that resulted in a 102 residue extension to the amino terminus |
| X2 Rhbdl1 (XM_006524022.1/XP_006524085.1) [3 forms] | Mouse | Secretase-type (6+1) | Alternate starting methionine that resulted in a 102 residue extension to the amino terminus and a removal of 54 residues after amino acid number 13 |
|  |  |  |  |
| X2 Rhbdl2 (XM_006503026.1/XP_006503089.1) [3 forms] | Mouse | Secretase-type (6+1) | A frameshift resulted in the shortening of the amino end to 9 residues |
|  |  |  |  |
| X3 Rhbdl3 (XM_006533328.1/XP_006533391.1) [7 forms] | Mouse | Secretase-type (6+1) | Removal of 8 residues after amino acid number 36 |
| X5 Rhbdl3 (XM_006533330.1/XP_006533393.1) [7 forms] | Mouse | Secretase-type (6+1) | Removal of 8 residues after amino acid number 36 |
| X6 Rhbdl3 (XM_006533331.1/XP_006533394.1) [7 forms] | Mouse | Secretase-type (6+1) | Removal of 53 residues removed after amino acid number 45, plus another removal of a segment from within amino end |
|  |  |  |  |
| X1 Rhbdd2 (XM_006504416.1/XP_006504479.1) [3 forms] | Mouse | distant relation | Alternate starting methionine that resulted in the removal of the whole amino end |
| X2 Rhbdd2 (XM_006504417.1/XP_006504480.1) [3 forms] | Mouse | distant relation | Alternate starting methionine that resulted in the removal of the whole amino end |
|  |  |  |  |
| Isoform 2 Derl2 (NM_001291146.1/NP_001278075.1) [4 forms] | Mouse | Rhomboid pseudoprotease | Alternate starting methionine that resulted in the removal of the whole amino end |
| Isoform 3 Derl2 (NM_001291147.1/NP_001278076.1) [4 forms] | Mouse | Rhomboid pseudoprotease | Alternate starting methionine that resulted in the removal of the whole amino end |
|  |  |  |  |
| X1 Derl3 (XM_006514079.1/XP_006514142.1) [2 forms] | Mouse | Rhomboid pseudoprotease | Addition of 85 extra residues upstream of the amino terminus |
|  |  |  |  |
| X2 Ubac2 (XM_006519493.1/XP_006519556.1) [3 forms] | Mouse | Rhomboid pseudoprotease | Alternate starting methionine that resulted in the removal of the amino end |
|  |  |  |  |
| RBL14 At3g17611 (NM_202600.1 / NP_974329.1) [3 forms] | Arabidopsis | Secretase (basic) | Alternate starting methionine that resulted in the removal of the amino end |
| RBL14 At3g17611 (NM_001084701.1 / NP_001078170.1) [3 forms] | Arabidopsis | Secretase (basic) | Alternate starting methionine that resulted in the removal of the amino end |
|  |  |  |  |
| RBL4 At3g53780 (NM_115238.2 / NP_566989.1) [2 forms] | Arabidopsis | Secretase type (6+1) | Alternate starting methionine that resulted in the removal of the amino end |
|  |  |  |  |
| RBL15 At3g58460 (NM_001203197.1/NP_001190126.1) [2 forms] | Arabidopsis | Secretase (basic) | A frameshift resulted in the addition of 23 residues at the amino terminus that changed the first 8 residues |
|  |  |  |  |
| Isoform B stet (NM_176272.2éNP_788450.1) [2 forms] | Drosophila | Secretase (basic) | Alternate starting methionine that resulted in the removal of the amino end |
|  |  |  |  |
| IsoformB ROM-4 (NM_001047549.2 / NP_001041014.1) [3 forms] | C elegans | Secretase (basic) | Alternate starting methionine that resulted in the removal of the amino end |
