# Supplementary material for: Alternative splice variants of rhomboid proteins: Comparative analysis of database entries for select model organisms and validation of functional potential
Source: F1000Res. 2018 May 31;7:139. Originally published 2018 Feb 1. [Version 2] doi: 10.12688/f1000research.13383.2 (PMC7065720; doi:10.12688/f1000research.13383.2)
Supplement: Supplementary file 7 [file f1000research-7-16431-s0007.tgz › cce5c9e8-3f8e-46f4-8069-4710eb69d9d2.zip!/Table S5.docx]

**Table S5: Summary of alternative splice variants impacting the L1 loop region.**

| **L1 Loop** | |  |  |
| --- | --- | --- | --- |
| **Rhomboid Name (accession #'s) [total forms]** | **Species** | **Rhomboid Type** | **Effect of Splicing** |
| Isoform2 PARL (NM_001037639.2 / NP_001032728.1) [10 forms] | Human | PARL | Removal of the last 14 residues, including the conserved H residue |
| Isoform4 PARL (NM_001324437.1 / NP_001322366.1) [10 forms] | Human | PARL | Removal of the last 14 residues, including the conserved H residue |
| X3 PARL (XM_017006802.1 / XP_016862291.1) [10 forms] | Human | PARL | Removal of the last 14 residues, including the conserved H residue |
| X4 PARL (XM_017006801.1 / XP_016862290.1) [10 forms] | Human | PARL | Removal of the last 14 residues, including the conserved H residue |
| X5 PARL (XM_017006803.1 / XP_016862292.1) [10 forms] | Human | PARL | Alternate methionine used that resulted in the removal of the last 14 residues, including the conserved H residue |
|  |  |  |  |
| X4 RHBDF1 iRhom1 (XM_017023557.1/XP_016879046.1) [7 forms] | Human | iRhom (evolved from PARL) | Missing residues |
| X5 RHBDF1 iRhom1 (XM_005255498.2/XP_005255555.1) [7 forms] | Human | iRhom (evolved from PARL) | Missing residues |
| X6 RHBDF1 iRhom1 (XM_017023558.1/XP_016879047.1) [7 forms] | Human | iRhom (evolved from PARL) | Missing residues |
|  |  |  |  |
| X4 RHBDF2 iRhom2 (XM_005257672.2/XP_005257729.1) [9 forms] | Human | iRhom (evolved from PARL) | Whole L1 Loop missing |
|  |  |  |  |
| X12 RHBDL3 (XM_017024280.1/XP_016879769.1) [14 forms] | Human | Secretase-type (6+1) | Most of the L1 loop deleted due to the used of an alternate methionine |
|  |  |  |  |
| Isoform b RHBDD2 (NM_001040457.2/NP_001035547.1) [6 Forms] | Human | distant relation | Whole L1 Loop is removed |
| Isoform b RHBDD2 (NM_001346186.1/NP_001333115.1) [6 Forms] | Human | distant relation | Whole L1 Loop is removed |
| Isoform b RHBDD2 (NM_001346187.1/NP_001333116.1) [6 Forms] | Human | distant relation | Whole L1 Loop is removed |
| Isoform c RHBDD2 (NM_001346188.1/NP_001333117.1) [6 Forms] | Human | distant relation | Whole L1 Loop is removed |
| Isoform d RHBDD2 (NM_001346189.1/NP_001333118.1) [6 Forms] | Human | distant relation | Whole L1 Loop is removed |
|  |  |  |  |
| X1 RHBDD3 (XM_017028750.1/XP_016884239.1) [5 forms] | Human | inactive distant relation | Whole L1 Loop is removed |
| X2 RHBDD3 (XM_006724224.3/XP_006724287.1) [5 forms] | Human | inactive distant relation | Whole L1 Loop is removed |
|  |  |  |  |
| Isoform c DERL1 (NM_001330601.1/NP_001317530.1) [4 forms] | Human | Rhomboid pseudoprotease | Alternate methionine used that resulted in the deletion of the whole L1 Loop |
| X2 DERL1 (XM_006716657.1/XP_006716720.1) [4 forms] | Human | Rhomboid pseudoprotease | Alternate methionine used that resulted in the deletion of the whole L1 Loop |
|  |  |  |  |
| Isoform c DERL2 (NM_001304779.1/NP_001291708.1) [3 forms] | Human | Rhomboid pseudoprotease | A frame shift that resulted in changes to the majority of the L1 Loop sequence |
|  |  |  |  |
| Isoform 2 UBAC2 (NM_177967.3/NP_808883.1) [7 forms] | Human | Rhomboid pseudoprotease | Alternate methionine used that resulted in a unique L1 Loop |
| X1 UBAC2 (XM_011521082.2/XP_011519384.1) [7 forms] | Human | Rhomboid pseudoprotease | Alternate methionine used that resulted in a unique L1 Loop |
| X2 UBAC2 (XM_006719948.3/XP_006720011.1) [7 forms] | Human | Rhomboid pseudoprotease | Alternate methionine used that resulted in a unique L1 Loop |
| X3 UBAC2 (XM_011521083.2/XP_011519385.1) [7 forms] | Human | Rhomboid pseudoprotease | Alternate methionine used that resulted in a unique L1 Loop |
| X4 UBAC2 (XM_011521084.2/XP_011519386.1) [7 forms] | Human | Rhomboid pseudoprotease | Alternate methionine used that resulted in a unique L1 Loop |
| X5 UBAC2 (XM_017020553.1/XP_016876042.1) [7 forms] | Human | Rhomboid pseudoprotease | Alternate methionine used that resulted in a unique L1 Loop |
|  |  |  |  |
| X12 Rhbdf1 iRhom1 (XM_006514503.1 / XP_006514566.1) [13 forms) | Mouse | iRhom (evolved from PARL) | Due to an alternate methionine used, the first 9 residues of L1 Loop are deleted |
|  |  |  |  |
| X1 Rhbdd2 (XM_006504416.1 / XP_006504479.1) [3 forms] | Mouse | distant relation | Whole L1 Loop is removed |
| X2 Rhbdd2 (XM_006504417.1 / XP_006504480.1) [3forms] | Mouse | distant relation | Whole L1 Loop is removed |
|  |  |  |  |
| Isoform 2 Derl2 (NM_001291146.1/NP_001278075.1) [4 forms] | Mouse | Rhomboid pseudoprotease | Alternate methionine used that resulted in deletion of the whole L1 loop |
| Isoform 3 Derl2 (NM_001291147.1/NP_001278076.1) [4 forms] | Mouse | Rhomboid pseudoprotease | Alternate methionine used that resulted in deletion of the whole L1 loop |
|  |  |  |  |
| X2 Ubac2 (XM_006519493.1/XP_006519556.1) [3 forms] | Mouse | Rhomboid pseudoprotease | Alternate start methionine used that resulted a deletion of the amino end of the L1 Loop |
|  |  |  |  |
| RBL14 At3g17611 (NM_202600.1 / NP_974329.1) [3 forms] | Arabidopsis | Secretase (basic) | Whole L1 Loop is removed |
| RBL14 At3g17611 (NM_001084701.1 / NP_001078170.1) [3 forms] | Arabidopsis | Secretase (basic) | Whole L1 Loop is removed |
|  |  |  |  |
| RBL4 At3g53780 (NM_115238.2 / NP_566989.1) [2 forms] | Arabidopsis | Secretase type (6+1) | Due to an alternate methionine used, the first 18 residues of the L1 Loop were deleted |
|  |  |  |  |
| IsoformB ROM-4 (NM_001047549.2 / NP_001041014.1) [3 forms] | C elegans | Secretase (basic) | Due to a frameshift, the L1 Loop is only 97 residues long, compared to 467 |
