# Supplementary material for: Alternative splice variants of rhomboid proteins: Comparative analysis of database entries for select model organisms and validation of functional potential
Source: F1000Res. 2018 May 31;7:139. Originally published 2018 Feb 1. [Version 2] doi: 10.12688/f1000research.13383.2 (PMC7065720; doi:10.12688/f1000research.13383.2)
Supplement: Supplementary file 7 [file f1000research-7-16431-s0007.tgz › cce5c9e8-3f8e-46f4-8069-4710eb69d9d2.zip!/Table S7.docx]

**Table S7: Summary of alternative splice variants impacting the L5 cap region.**

| **L5 Cap & TMD5** | |  |  |
| --- | --- | --- | --- |
| **Rhomboid Name (accession #'s) [total forms]** | **Species** | **Rhomboid Type** | **Effect of Splicing** |
| Isoform 5 (NM_001324438.1/NP_001311367.1) [10 forms] | Human | PARL | L5 Cap is deleted due to early termination |
|  |  |  |  |
| X6 RHBDF1 iRhom1 (XM_017023558.1/XP_016879047.1) [7 forms] | Human | iRhom (evolved from PARL) | L5 Cap is deleted due to early termination |
|  |  |  |  |
| X1 RHBDL1 (XM_017023849.1/XP_016879338.1) [5 forms] | Human | Secretase-type (6+1) | A frameshift changes the carboxyl terminal sequence |
| X2 RHBDL1 (XM_017023850.1/XP_016879339.1) [5 forms] | Human | Secretase-type (6+1) | A frameshift resulted in early termination |
|  |  |  |  |
| X1 RHBDL3 (XM_017024272.1/XP_016879761.1) [14 forms] | Human | Secretase-type (6+1) | A frameshift changes the carboxyl terminal sequence |
| X2 RHBDL3 (XM_017024275.1/XP_016879764.1) [14 forms] | Human | Secretase-type (6+1) | A frameshift changes the carboxyl terminal sequence |
| X3 RHBDL3 (XM_017024273.1/XP_016879762.1) [14 forms] | Human | Secretase-type (6+1) | A frameshift changes the carboxyl terminal sequence |
| X4 RHBDL3 (XM_017024276.1/XP_016879765.1) [14 forms] | Human | Secretase-type (6+1) | A frameshift changes the carboxyl terminal sequence |
| X9 RHBDL3 (XM_017024274.1/XP_016879763.1) [14 forms] | Human | Secretase-type (6+1) | A frameshift changes the carboxyl terminal sequence |
| X10 RHBDL3 (XM_017024278.1/XP_016879767.1) [14 forms] | Human | Secretase-type (6+1) | A frameshift changes the carboxyl terminal sequence |
| X11 RHBDL3 (XM_017024277.1/XP_016879766.1) [14 forms] | Human | Secretase-type (6+1) | A frameshift changes the carboxyl terminal sequence |
| X12 RHBDL3 (XM_017024280.1/XP_016879769.1) [14 forms] | Human | Secretase-type (6+1) | A frameshift changes the carboxyl terminal sequence |
|  |  |  |  |
| Isoform c DERL2 (NM_001304779.1/NP_001291708.1) [3 forms] | Human | Rhomboid pseudoprotease | Early termination resulted in the deletion of the L5 cap |
|  |  |  |  |
| X3 DERL3 (XM_017029080.1/XP_016884569.1) [10 forms] | Human | Rhomboid pseudoprotease | A frameshift resulted in changes to the sequence |
| X4 DERL3 (XM_017029079.1/XP_016884568.1) [10 forms] | Human | Rhomboid pseudoprotease | A frameshift resulted in changes to the sequence |
| X5 DERL3 (XM_017029078.1/XP_016884567.1) [10 forms] | Human | Rhomboid pseudoprotease | A frameshift resulted in changes to the sequence |
| X7 DERL3 (XM_017029081.1/XP_016884570.1) [10 forms] | Human | Rhomboid pseudoprotease | A frameshift resulted in changes to the sequence |
|  |  |  |  |
| X1 Rhbdf1 iRhom1 (XM_006514492.1/XP_006514555.1) [13 forms] | Mouse | iRhom (evolved from PARL) | 12 residues deleted from TMD5 and 3 residues deleted from the L5 cap. TMD5 residues that remain are unique |
| X2 Rhbdf1 iRhom1 (XM_006514493.1/XP_006514556.1) [13 forms] | Mouse | iRhom (evolved from PARL) | 9 residues deleted from TMD5 and 3 residues deleted from the L5 cap |
| X3 Rhbdf1 iRhom1 (XM_006514494.1/XP_006514557.1) [13 forms] | Mouse | iRhom (evolved from PARL) | 12 residues deleted from TMD5 and 3 residues deleted from the L5 cap. TMD5 residues that remain are unique |
| X4 Rhbdf1 iRhom1 (XM_006514495.1/XP_006514558.1) [13 forms] | Mouse | iRhom (evolved from PARL) | 12 residues deleted from TMD5 and 3 residues deleted from the L5 cap. TMD5 residues that remain are unique |
| X5 Rhbdf1 iRhom1 (XM_006514496.1/XP_006514559.1) [13 forms] | Mouse | iRhom (evolved from PARL) | 12 residues deleted from TMD5 and 3 residues deleted from the L5 cap. TMD5 residues that remain are unique |
| X7 Rhbdf1 iRhom1 (XM_006514498.1/XP_006514561.1) [13 forms] | Mouse | iRhom (evolved from PARL) | 12 residues deleted from TMD5 and 3 residues deleted from the L5 cap. TMD5 residues that remain are unique |
| X8 Rhbdf1 iRhom1 (XM_006514499.1/XP_006514562.1) [13 forms] | Mouse | iRhom (evolved from PARL) | 12 residues deleted from TMD5 and 3 residues deleted from the L5 cap. TMD5 residues that remain are unique |
| X10 Rhbdf1 iRhom1 (XM_006514501.1/XP_006514564.1) [13 forms] | Mouse | iRhom (evolved from PARL) | 12 residues deleted from TMD5 and 3 residues deleted from the L5 cap. TMD5 residues that remain are unique |
| X12 Rhbdf1 iRhom1 (XM_006514503.1/XP_006514566.1) [13 forms] | Mouse | iRhom (evolved from PARL) | 12 residues deleted from TMD5 and 3 residues deleted from the L5 cap. TMD5 residues that remain are unique |
|  |  |  |  |
| X1 Rhbdf2 iRhom2 (XM_006533108.1/XP_006533171.1) [3 forms] | Mouse | iRhom (evolved from PARL) | A frameshift within TMD2 that resulted in changes to the downstream residues |
|  |  |  |  |
| X1 Rhbdl3 (XM_006533326.1/XP_006533389.1) [7 forms] | Mouse | Secretase-type (6+1) | A frameshift within TMD2 that resulted in changes to the downstream residues |
| X2 Rhbdl3 (XM_006533327.1/XP_006533390.1) [7 forms] | Mouse | Secretase-type (6+1) | A frameshift within TMD2 that resulted in changes to the downstream residues |
| X3 Rhbdl3 (XM_006533328.1/XP_006533391.1) [7 forms] | Mouse | Secretase-type (6+1) | A frameshift within TMD2 that resulted in changes to the downstream residues |
| X6 Rhbdl3 (XM_006533331.1/XP_006533394.1) [7 forms] | Mouse | Secretase-type (6+1) | A frameshift within TMD2 that resulted in changes to the downstream residues |
|  |  |  |  |
| RBL1 At2g29050 (NM_001084504.1/NP_001077973.1) [2 forms] | Arabidopsis | Secretase-type (6+1) | The Linker between TMD4 and the gate contained an additional 28 residues |
|  |  |  |  |
| RBL3 At5g07250 (NM_001125710.1/NP_001119182.1) [2 forms] | Arabidopsis | Secretase-type (6+1) | Gate (and catalytic TMD 6 with H) was spliced out |
|  |  |  |  |
| IsoformB ROM-4 (NM_001047549.2 / NP_001041014.1) [3 forms] | C elegans | Secretase (basic) | Alternate start methionine and a frameshift within the L1 loop resulted in unrelated residues and a restoration of the downstream sequence |
