# Supplementary material for: Alternative splice variants of rhomboid proteins: Comparative analysis of database entries for select model organisms and validation of functional potential
Source: F1000Res. 2018 May 31;7:139. Originally published 2018 Feb 1. [Version 2] doi: 10.12688/f1000research.13383.2 (PMC7065720; doi:10.12688/f1000research.13383.2)
Supplement: Supplementary file 7 [file f1000research-7-16431-s0007.tgz › cce5c9e8-3f8e-46f4-8069-4710eb69d9d2.zip!/Table S8.docx]

**Table S8: Summary of alternative splice variants impacting the carboxyl terminus.**

| **Carboxyl End** | |  |  |
| --- | --- | --- | --- |
| **Rhomboid Name (accession #'s) [total forms]** | **Species** | **Rhomboid Type** | **Effect of Splicing** |
| Isoform 5 PARL (NM_001324438.1/NP_001311367.1) [10 forms] | Human | PARL | A frameshift that resulted in a unique carboxyl terminal sequence with the addition of 6 residues |
| X1 PARL (XM_017006800.1/XP_016862289.1) [10 forms] | Human | PARL | A frameshift that resulted in a unique carboxyl terminal sequence with the addition of 6 residues |
| X2 PARL (XM_005247582.4/XP_005247639.1) [10 forms] | Human | PARL | A frameshift that resulted in a unique carboxyl terminal sequence with the addition of 6 residues |
| X3 PARL (XM_017006802.1/XP_016862291.1) [10 forms] | Human | PARL | A frameshift that resulted in a unique carboxyl terminal sequence with the addition of 6 residues |
| X4 PARL (XM_005247585.2/XP_005247642.1) [10 forms] | Human | PARL | A frameshift that resulted in a unique carboxyl terminal sequence with the addition of 6 residues |
| X5 PARL (XM_005247586.2/XP_005247643.1) [10 forms] | Human | PARL | A frameshift that resulted in a unique carboxyl terminal sequence with the addition of 6 residues |
|  |  |  |  |
| X6 RHBDF1 iRhom1 (XM_017023558.1/XP_016879047.1) [7 forms] | Human | iRhom (evolved from PARL) | An early termination resulted in deletions to most of the carboxyl terminal sequence |
|  |  |  |  |
| X1 RHBDL1 (XM_017023849.1/XP_016879338.1) [5 forms] | Human | Secretase-type (6+1) | A frameshift that resulted in a unique sequence |
| X2 RHBDL1 (XM_017023850.1/XP_016879339.1) [5 forms] | Human | Secretase-type (6+1) | An early termination resulted in deletions to most of the carboxyl terminal sequence |
|  |  |  |  |
| X1 RHBDL3 (XM_017024272.1/XP_016879761.1) [14 forms] | Human | Secretase-type (6+1) | A frameshift that resulted in a unique sequence |
| X2 RHBDL3 (XM_017024275.1/XP_016879764.1) [14 forms] | Human | Secretase-type (6+1) | A frameshift that resulted in a unique sequence |
| X3 RHBDL3 (XM_017024273.1/XP_016879762.1) [14 forms] | Human | Secretase-type (6+1) | A frameshift that resulted in a unique sequence |
| X4 RHBDL3 (XM_017024276.1/XP_016879765.1) [14 forms] | Human | Secretase-type (6+1) | A frameshift that resulted in a unique sequence |
| X9 RHBDL3 (XM_017024274.1/XP_016879763.1) [14 forms] | Human | Secretase-type (6+1) | A frameshift that resulted in a unique sequence |
| X10 RHBDL3 (XM_017024278.1/XP_016879767.1) [14 forms] | Human | Secretase-type (6+1) | A frameshift that resulted in a unique sequence |
| X11 RHBDL3 (XM_017024277.1/XP_016879766.1) [14 forms] | Human | Secretase-type (6+1) | A frameshift that resulted in a unique sequence |
| X12 RHBDL3 (XM_017024280.1/XP_016879769.1) [14 forms] | Human | Secretase-type (6+1) | A frameshift that resulted in a unique sequence |
|  |  |  |  |
| X3 RHBDD1 (XM_017005094.1/XP_016860583.1) [15 forms] | Human | Secretase-type (basic) | A frameshift that resulted in changes to the sequence and early termination |
| X12 RHBDD1 (XM_017005093.1/XP_016860582.1) [15 forms] | Human | Secretase-type (basic) | A frameshift that resulted in changes to the sequence and early termination |
| X13 RHBDD1 (XM_017005095.1/XP_016860584.1) [15 forms] | Human | Secretase-type (basic) | A frameshift that resulted in changes to the sequence and early termination |
|  |  |  |  |
| Isoform c DERL2 (NM_001304779.1/NP_001291708.1) [3 forms] | Human | Rhomboid pseudoprotease | An early termination resulted in deletion of the carboxyl terminal sequence |
|  |  |  |  |
| Isoform 2 DERL3 (NM_001002862.2/NP_001002862.1) [10 forms] | Human | Rhomboid pseudoprotease | Unique sequence |
| Isoform 3 DERL3 (NM_198440.3/NP_940842.2) [10 forms] | Human | Rhomboid pseudoprotease | Unique sequence |
| X1 DERL3 (XM_011530506.2/XP_011528808.1) [10 forms] | Human | Rhomboid pseudoprotease | Unique sequence |
| X2 DERL3 (XM_017029082.1/XP_016884571.1) [10 forms] | Human | Rhomboid pseudoprotease | Unique sequence |
| X3 DERL3 (XM_017029080.1/XP_016884569.1) [10 forms] | Human | Rhomboid pseudoprotease | Unique sequence |
| X4 DERL3 (XM_017029079.1/XP_016884568.1) [10 forms] | Human | Rhomboid pseudoprotease | Unique sequence |
| X5 DERL3 (XM_017029078.1/XP_016884567.1) [10 forms] | Human | Rhomboid pseudoprotease | Unique sequence |
| X6 DERL3 (XM_011530505.2/XP_011528807.1) [10 forms] | Human | Rhomboid pseudoprotease | Unique sequence |
| X6 DERL3 (XM_017029081.1/XP_016884570.1) [10 forms] | Human | Rhomboid pseudoprotease | Unique sequence |
|  |  |  |  |
| X1 Rhbf1 iRhom1 (XM_006514492.1/XP_006514555.1) [13 forms] | Mouse | iRhom (evolved from PARL) | A frameshift after the catalytic GPAG changes the sequence of the carboxyl end |
| X2 Rhbf1 iRhom1 (XM_006514493.1/XP_006514556.1) [13 forms] | Mouse | iRhom (evolved from PARL) | Frameshift after L1 Loop changes downstream sequence, generates an early termination |
| X3 Rhbf1 iRhom1 (XM_006514494.1/XP_006514557.1) [13 forms] | Mouse | iRhom (evolved from PARL) | A frameshift after the catalytic GPAG changes the sequence of the carboxyl end |
| X4 Rhbf1 iRhom1 (XM_006514495.1/XP_006514558.1) [13 forms] | Mouse | iRhom (evolved from PARL) | A frameshift after the catalytic GPAG changes the sequence of the carboxyl end |
| X5 Rhbf1 iRhom1 (XM_006514496.1/XP_006514559.1) [13 forms] | Mouse | iRhom (evolved from PARL) | A frameshift after the catalytic GPAG changes the sequence of the carboxyl end |
| X7 Rhbf1 iRhom1 (XM_006514498.1/XP_006514561.1) [13 forms] | Mouse | iRhom (evolved from PARL) | A frameshift after the catalytic GPAG changes the downstream sequence, but restores itself in the carboxyl end where the last 25 residues are sustained |
| X8 Rhbf1 iRhom1 (XM_006514499.1/XP_006514562.1) [13 forms] | Mouse | iRhom (evolved from PARL) | A frameshift after the catalytic GPAG changes the sequence of the carboxyl end |
| X10 Rhbf1 iRhom1 (XM_006514501.1/XP_006514564.1) [13 forms] | Mouse | iRhom (evolved from PARL) | A frameshift after the catalytic GPAG changes the sequence of the carboxyl end |
| X12 Rhbf1 iRhom1 (XM_006514503.1/XP_006514566.1) [13 forms] | Mouse | iRhom (evolved from PARL) | A frameshift after the catalytic GPAG changes the sequence of the carboxyl end |
|  |  |  |  |
| X1 Rhbdf2 iRhom2 (XM_006533108.1/XP_006533171.1) [3 forms] | Mouse | iRhom (evolved from PARL) | A frameshift occurred after the L1 Loop, and an early termination after the catalytic GPAG |
|  |  |  |  |
| X1 Rhbdl3 (XM_006533326.1/XP_006533389.1) [7 forms] | Mouse | Secretase-type (6+1) | A frameshift within TMD3 changes downstream residues and an extended carboxyl terminus |
| X2 Rhbdl3 (XM_006533327.1/XP_006533390.1) [7 forms] | Mouse | Secretase-type (6+1) | A frameshift within TMD3 changes downstream residues and an extended carboxyl terminus |
| X3 Rhbdl3 (XM_006533328.1/XP_006533391.1) [7 forms] | Mouse | Secretase-type (6+1) | A frameshift within TMD3 changes downstream residues and an extended carboxyl terminus |
| X6 Rhbdl3 (XM_006533331.1/XP_006533394.1) [7 forms] | Mouse | Secretase-type (6+1) | A frameshift within TMD3 changes downstream residues and an extended carboxyl terminus |
|  |  |  |  |
| At1g74130 (NM_202413.1/NP_974141.1) [4 forms] | Arabidopsis | Inactive homologue | Alternative splicing within the linker between TMD5 and 6 resulted in an early termination by deleting 26 residues and the carboxyl terminus |
| At1g74130 (S 6/7) [4 forms] | Arabidopsis | Inactive homologue | Alternative splicing within the linker between TMD5 and 6 resulted in an early termination by deleting 41 residues and the carboxyl terminus |
| At1g74130 (M 7/8) [4 forms] | Arabidopsis | Inactive homologue | Alternative splicing within the linker between TMD5 and 6 resulted in an early termination by deleting 23 residues and the carboxyl terminus |
|  |  |  |  |
| RBL3 At5g07250 (NM_001125710.1/NP_001119182.1) [2 forms] | Arabidopsis | Secretase-type (6+1) | First 4 residues of the carboxyl end deleted but the downstream sequence was sustained as a result of the removal of peptides within the |
