# Supplementary material for: Alternative splice variants of rhomboid proteins: Comparative analysis of database entries for select model organisms and validation of functional potential
Source: F1000Res. 2018 May 31;7:139. Originally published 2018 Feb 1. [Version 2] doi: 10.12688/f1000research.13383.2 (PMC7065720; doi:10.12688/f1000research.13383.2)
Supplement: Supplementary file 7 [file f1000research-7-16431-s0007.tgz › cce5c9e8-3f8e-46f4-8069-4710eb69d9d2.zip!/Table S9.docx]

**Table S9: Summary of alternative splice variants impacting other regions and motifs.**

| **Other Motifs** | |  |  |
| --- | --- | --- | --- |
| **Rhomboid Name (accession #'s) [total forms]** | **Species** | **Rhomboid Type** | **Effect of Splicing** |
| Isoform 2 (NM_001037639.2 / NP_001032728.1) [10 forms] | Human | PARL | Removal of TMD3, including the conserved HxxxN. There is no loop 2 and the deletion of the first 12 residues of TMD4. |
| Isoform 4 (NM_001324437.1 / NP_001311366.1) [10 forms] | Human | PARL | Removal of TMD3, including the conserved HxxxN. There is no loop 2 and the deletion of the first 12 residues of TMD4. |
| X3 PARL (XM_017006802.1 / XP_016862291.1) [10 forms] | Human | PARL | Removal of TMD3, including the conserved HxxxN. There is no loop 2 and the deletion of the first 12 residues of TMD4. |
| X4 PARL (XM_017006801.1 / XP_016862290.1) [10 forms] | Human | PARL | Removal of TMD3, including the conserved HxxxN. There is no loop 2 and the deletion of the first 12 residues of TMD4. |
| X5 PARL (XM_017006803.1 / XP_016862292.1) [10 forms] | Human | PARL | An alternate starting methionine used that resulted in the deletion of the first two TMD's, plus the removal of TMD3, including the conserved HxxxN. There is no loop 2 and the deletion of the first 12 residues of TMD4. |
|  |  |  |  |
| X2 RHBDF1 iRhom1 (XM_017023556.1/XP_016879045.1) [7 forms] | Human | iRhom (evolved from PARL) | Additional sequence following the amino terminus |
| X3 RHBDF1 iRhom1 (XM_006720921.1/XP_006720984.1) [7 forms] | Human | iRhom (evolved from PARL) | Additional sequence following the amino terminus |
| X4 RHBDF1 iRhom1 (XM_017023557.1/XP_016879046.1) [7 forms] | Human | iRhom (evolved from PARL) | Additional sequence following the amino terminus |
| X5 RHBDF1 iRhom1 (XM_005255498.2/XP_005255555.1) [7 forms] | Human | iRhom (evolved from PARL) | Alternate starting methionine used that resulted in the deletion of TMD1 |
| X6 RHBDF1 iRhom1 (XM_017023558.1/XP_016879047.1) [7 forms] | Human | iRhom (evolved from PARL) | Additional sequence following the amino terminus |
|  |  |  |  |
| Isoform 2 RHBDF2 iRhom2 (NM_001005498.3/NP_001005498.2) [8 forms] | Human | iRhom (evolved from PARL) | Residues missing following the amino terminus |
| X1 RHBDF2 iRhom2 (XM_011525250.2/XP_011523552.1) [8 forms] | Human | iRhom (evolved from PARL) | Residues missing following the amino terminus |
| X2 RHBDF2 iRhom2 (XM_011525251.2/XP_011523553.1) [8 forms] | Human | iRhom (evolved from PARL) | Residues missing following the amino terminus |
| X3 RHBDF2 iRhom2 (XM_005257670.1/XP_005257727.1) [8 forms] | Human | iRhom (evolved from PARL) | Residues missing following the amino terminus |
| X4 RHBDF2 iRhom2 (XM_017025079.1/XP_016880568.1) [8 forms] | Human | iRhom (evolved from PARL) | Residues missing following the amino terminus |
| X6 RHBDF2 iRhom2 (XM_011525249.2/XP_011523551.1) [8 forms] | Human | iRhom (evolved from PARL) | Residues missing following the amino terminus |
|  |  |  |  |
| Isoform2 RHBDL1 (NM_001278721.1/NP_001265650.1) [5 forms] | Human | Secretase-type (6+1) | Residues removed within the sequence |
| Isoform3 RHBDL1 (NM_001318733.1/NP_001305662.1) [5 forms] | Human | Secretase-type (6+1) | Alternate methionine used, plus additional residues |
| X1 RHBDL1 (XM_017023849.1/XP_016879338.1) [5 forms] | Human | Secretase-type (6+1) | Alternate methionine used, plus additional residues. A frameshift also changed the downstream sequence. |
| X2 RHBDL1 (XM_017023850.1/XP_016879339.1) [5 forms] | Human | Secretase-type (6+1) | Alternate methionine used, plus additional residues. A frameshift also changed the downstream sequence. |
|  |  |  |  |
| Isoform 2 RHBDL3 (NM_001330181.1/NP_001317110.1) [13 forms] | Human | Secretase-type (6+1) | Unique sequence |
| X1 RHBDL3 (XM_017024272.1/XP_016879761.1) [13 forms] | Human | Secretase-type (6+1) | Unique sequence |
| X2 RHBDL3 (XM_017024275.1/XP_016879764.1) [13 forms] | Human | Secretase-type (6+1) | Unique sequence |
| X3 RHBDL3 (XM_017024273.1/XP_016879762.1) [13 forms] | Human | Secretase-type (6+1) | Unique sequence |
| X4 RHBDL3 (XM_017024276.1/XP_016879765.1) [13 forms] | Human | Secretase-type (6+1) | Unique sequence |
| X6 RHBDL3 (XM_011524443.2/XP_011522745.1) [13 forms] | Human | Secretase-type (6+1) | Unique sequence |
| X7 RHBDL3 (XM_017024279.1/XP_016879768.1) [13 forms] | Human | Secretase-type (6+1) | Unique sequence |
| X8 RHBDL3 (XM_006721734.3/XP_006721797.1) [13 forms] | Human | Secretase-type (6+1) | Unique sequence |
| X9 RHBDL3 (XM_017024274.1/XP_016879763.1) [13 forms] | Human | Secretase-type (6+1) | Unique sequence |
| X10 RHBDL3 (XM_017024278.1/XP_016879767.1) [13 forms] | Human | Secretase-type (6+1) | Unique sequence |
| X11 RHBDL3 (XM_017024277.1/XP_016879766.1) [13 forms] | Human | Secretase-type (6+1) | Unique sequence |
| X12 RHBDL3 (XM_017024280.1/XP_016879769.1) [13 forms] | Human | Secretase-type (6+1) | Unique sequence |
|  |  |  |  |
| Isoform b RHBDD2 (NM_001040457.2/NP_001035547.1) [6 forms] | Human | distant relation | Parts of the sequence is unique |
| Isoform b RHBDD2 (NM_001346186.1/NP_001333115.1) [6 forms] | Human | distant relation | Parts of the sequence is unique |
| Isoform b RHBDD2 (NM_001346187.1/NP_001333116.1) [6 forms] | Human | distant relation | Parts of the sequence is unique |
| Isoform c RHBDD2 (NM_001346188.1/NP_001333117.1) [6 forms] | Human | distant relation | Parts of the sequence is unique |
| Isoform d RHBDD2 (NM_001346189.1/NP_001333118.1) [6 forms] | Human | distant relation | Parts of the sequence is unique |
|  |  |  |  |
| X1 RHBDD3 (XM_017028750.1/XP_016884239.1) [5 forms] | Human | distant relation | Alternate methionine used that resulted in many changes to the sequence |
| X2 RHBDD3 (XM_006724224.3/XP_006724287.1) [5 forms] | Human | distant relation | Parts of the sequence is missing |
|  |  |  |  |
| Isoform c DERL1 (NM_001330601.1/NP_001317530.1) [4 forms] | Human | Rhomboid pseudoprotease | Alternate methionine used that resulted in deletions to most of the amino end |
| X2 DERL1 (XM_006716657.1/XP_006716720.1) [4 forms] | Human | Rhomboid pseudoprotease | Alternate methionine used that resulted in deletions to most of the amino end |
|  |  |  |  |
| Isoform c DERL2 (NM_001304779.1/NP_001291708.1) [3 forms] | Human | Rhomboid pseudoprotease | Early termination |
|  |  |  |  |
| X3 DERL3 (XM_017029080.1/XP_016884569.1) [10 forms] | Human | Rhomboid pseudoprotease | Unique residues |
| X4 DERL3 (XM_017029079.1/XP_016884568.1) [10 forms] | Human | Rhomboid pseudoprotease | Unique residues |
| X5 DERL3 (XM_017029078.1/XP_016884567.1) [10 forms] | Human | Rhomboid pseudoprotease | Unique residues |
|  |  |  |  |
| Isoform 2 UBAC2 (NM_177967.3/NP_808883.1) [7 forms] | Human | Rhomboid pseudoprotease | Unique residues |
| X1 UBAC2 (XM_011521082.2/XP_011519384.1) [7 forms] | Human | Rhomboid pseudoprotease | Unique residues |
| X2 UBAC2 (XM_006719948.3/XP_006720011.1) [7 forms] | Human | Rhomboid pseudoprotease | Unique residues |
| X3 UBAC2 (XM_011521083.2/XP_011519385.1) [7 forms] | Human | Rhomboid pseudoprotease | Unique residues |
| X4 UBAC2 (XM_011521084.2/XP_011519386.1) [7 forms] | Human | Rhomboid pseudoprotease | Unique residues |
| X5 UBAC2 (XM_017020553.1/XP_016876042.1) [7 forms] | Human | Rhomboid pseudoprotease | Unique residues |
|  |  |  |  |
| X2 Rhbdf1 iRhom1 (XM_006514493.1/XP_006514556.1) [13 forms] | Mouse | iRhom (evolved from PARL) | A frameshift of 1 amino acid within TMD2 changed the downstream sequence |
| X12 Rhbdf1 iRhom1 (XM_006514503.1/XP_006514566.1) [13 forms] | Mouse | iRhom (evolved from PARL) | Alternate methionine used that resulted in the deletion of TMD1 |
|  |  |  |  |
| X1 Rhbdf2 iRhom2 XM_006533108.1/XP_006533171.1() [3 forms] | Mouse | iRhom (evolved from PARL) | A frameshift within TMD2 changed the downstream sequence |
|  |  |  |  |
| X2 Rhbdl2 (XM_006503026.1/XP_006503089.1) [3 forms] | Mouse | Secretase-type (6+1) | First residue of TMD1 was deleted |
|  |  |  |  |
| X1 Rhbdl3 (XM_006533326.1/XP_006533389.1) [7 forms] | Mouse | Secretase-type (6+1) | A frameshift within TMD3 changed the catalytic dyad and the entire sequence |
| X2 Rhbdl3 (XM_006533327.1/XP_006533390.1) [7 forms] | Mouse | Secretase-type (6+1) | A frameshift within TMD3 changed the catalytic dyad and the entire sequence |
| X3 Rhbdl3 (XM_006533328.1/XP_006533391.1) [7 forms] | Mouse | Secretase-type (6+1) | A frameshift within TMD3 changed the catalytic dyad and the entire sequence |
| X6 Rhbdl3 (XM_006533331.1/XP_006533394.1) [7 forms] | Mouse | Secretase-type (6+1) | A frameshift within TMD3 changed the catalytic dyad and the entire sequence |
|  |  |  |  |
| X1 Rhbdd2 (XM_006504416.1/XP_006504479.1) [3 forms] | Mouse | inactive distant relation | Alternate methionine used resulted in the deletion of the first 142 residues |
| X2 Rhbdd2 (XM_006504417.1/XP_006504480.1) [3 forms] | Mouse | inactive distant relation | Alternate methionine used resulted in the deletion of the first 142 residues |
|  |  |  |  |
| X5 Rhbdd3 (XM_006514717.1/XP_006514780.1) [6 forms] | Mouse | inactive distant relation | A frameshift in the middle changed sequence, but restored downstream |
|  |  |  |  |
| Isoform 2 Derl2 (NM_001291146.1/NP_001278075.1) [4 forms] | Mouse | Rhomboid pseudoprotease | Alternate methionine used resulted in the deletion of TMD1 and the first 2 residues in TMD2 |
| Isoform 3 Derl2 (NM_001291147.1/NP_001278076.1) [4 forms] | Mouse | Rhomboid pseudoprotease | Alternate methionine used resulted in the deletion of TMD1 and the first 2 residues in TMD2 |
|  |  |  |  |
| X1 Ubac2 (XM_006519492.1/XP_006519555.1) [3 forms] | Mouse | Rhomboid pseudoprotease | TMD4 is missing |
| X2 Ubac2 (XM_006519493.1/XP_006519556.1) [3 forms] | Mouse | Rhomboid pseudoprotease | Alternate starting methionine resulted in the deletion of the sequence upstream of the last 6 residues of TMD4 |
|  |  |  |  |
| RBL14 At3g17611 (NM_202600.1 / NP_974329.1) [3 forms] | Arabidopsis | Secretase (basic) | Alternate start methionine used resulted in the deletion of TMD1 and 8 residues into TMD2, including the conserved HxxxN motif |
| RBL14 At3g17611 (NM_001084701.1 / NP_001078170.1) [3 forms] | Arabidopsis | Secretase (basic) | Alternate starting methionine used resulted in the deletion of TMD1, TMD2 and TMD3 |
|  |  |  |  |
| RBL4 At3g53780 (NM_115238.2 / NP_566989.1) [2 forms] | Arabidopsis | Secretase type (6+1) | Alternate methionine used resulted in the deletion of TMD1 |
|  |  |  |  |
| RBL8 At1g25290 (NM_102339.3/NP_173900.2) [3 forms] | Arabidopsis | Secretase (basic) | 4 additional residues added before the RVL motif |
| RBL8/RBL10 At1g25290 (No accession - Short form) [3 forms] | Arabidopsis | Secretase (basic) | The 3 residue RVL motif was deleted |
|  |  |  |  |
| IsoformB ROM-4 (NM_001047549.2 / NP_001041014.1) [3 forms] | C elegans | Secretase (basic) | Alternate starting methionine and a frameshift resulted in a substantial deletion of the sequence |
