# Supplementary material for: Alternative splice variants of rhomboid proteins: Comparative analysis of database entries for select model organisms and validation of functional potential
Source: F1000Res. 2018 May 31;7:139. Originally published 2018 Feb 1. [Version 2] doi: 10.12688/f1000research.13383.2 (PMC7065720; doi:10.12688/f1000research.13383.2)
Supplement: Peer review history

# Peer review: Reviewer response for version 2

**DOI:** 10.5256/f1000research.16431.r34594

**Published:** 2020-03-09

**Authors:** Yuriy L Orlov

**Affiliations:** 1 Institute of Cytology and Genetics (SB RAS), Siberian Branch of the Russian Academy of Sciences (SB RAS), Novosibirsk, Russian Federation; 2 Novosibirsk State University, Novosibirsk, Russian Federation; 3 Sechenov University, Moscow, Russian Federation

I have no more comments. I just recommend to make the citations separately (like 5-8, 9-12, 14-20), citing one-two papers in time after each phrase or word. Such citations go in bulk, not convenient for readers.

I confirm that I have read this submission and believe that I have an appropriate level of expertise to confirm that it is of an acceptable scientific standard.

# Peer review: Reviewer response for version 2

**DOI:** 10.5256/f1000research.16431.r34592

**Published:** 2018-06-22

**Authors:** Martin Kollmar

**Affiliations:** 1 Group Systems Biology of Motor Proteins, Department of NMR‐Based Structural Biology, Max‐Planck‐Institute for Biophysical Chemistry, Göttingen, Germany

The authors Powles and Ko now provide a revised version. Although this version is improved compared to the previous version, the authors did not follow my suggestion to revise the manuscript except for the sentences, which I explicitly stated. The manuscript is still full of awkward phrases, where it is clear what the authors want to say but which read wrong as they are written. It is not my but the authors’ task to carefully go through the manuscript and correct all these issues. Citations are still extremely unbalanced. In the introduction and discussion, citations are pooled, while at many statements that would warrant citations those are missing. I also cannot accept the sequence-based analysis as it is. If the authors prefer not to revise it I would suggest to completely remove it or boil it down to a single schematic figure and at most half a page of text. As it is, it is completely useless for further research: researchers not aware of the problems with sequence analyses will be misguided, and researchers interested in sequence aspects will need to do a correct analysis anyway. The supplementary Figures S1 to S8 do not show any useful information (the figure legends do not describe what is shown, the structures in “A” do not correspond to the sequences shown in “B”) and need to be completely removed. The authors do not understand what RNA-seq mapping means and everything in the manuscript mentioning RNA-seq needs to be removed. The authors write “Entries were retrieved from databases for cDNA/EST sequences, RNA-seq,…”, but where are these entries? Where is the list of reads from SRA archives supporting any of the alternative splice variants? Which cDNA/EST clones support which splice variant? Many (especially the human) splice variants result from COMBINATIONS of alternatively spliced exons, e.g. from 2 alternatively spliced exons you might get 4 different transcripts and this exponentially increases with the number of alternatively spliced exons per gene. Where is the evidence (full-length cDNA) that all these combinations are found? Are there coupled splicing events? How does the number of “entries” reported (e.g. 95 for human) correspond to the number of possible combinations, and by which evidence are certain combinations excluded? By comparing human and mouse, isn’t it just a database problem that mouse contains less entries than human, although the total number of potential combinations is identical?

 In their response letter, the authors write “We did not include references dealing with alternative splicing because the study was not intended to be at this level, but working at the level of surveying and comparing available entries and what these entries may reflect at the protein level.  As described above, substantial revisions were focused on clarifying the intent of the work to avoid this impression.  This reasoning applies to why gene structures and splicing events were not provided.”. If the authors do not want to deal with alternative splicing at the gene structure level, than the provided data/figures are even more misleading and misguiding. What is the benefit for the reader to have all these figures (main figs and supplementary data/figs) if these are not based on and supported by real data? Either, do a thorough analysis based on gene structures and real RNA-seq mapping, or remove all the speculations on predicted sequences and structures (text, figs, supplements). Do the authors know which software was used to generate these predictions? Re-run the gene prediction software and you will get more predictions to speculate on.

 => Abstract, Results section, first sentence: “A comparison of database entries of six widely used eukaryotic experimental models […] revealed robust usage of alternative splicing…”. I think that the word “reveal” is overstating the findings, the database entries just “suggest” that alternative splicing might happen. What is “robust usage of alternative splicing”?

 => Introduction, first paragraph: Lemberg and Freeman do not mention “Derlin” in their paper at all. The current phrasing of this sentence is misleading in this respect. As far as I understand the Lemberg and Freeman paper (see Fig. 4 there), they distinguish 3 types of rhomboids, and the proteolytically active ones further subdivide into two subgroups. The sentence by Powles and Ko “Phylogenetic studies, such as that conducted by Lemberg and Freeman (2007), suggest that rhomboid genes can be divided into two subgroups encoding proteolytically active secretases- and Presenilin Associated Rhomboid Like (PARL)-type rhomboid forms, and two subgroups of catalytically inactive forms (“non-proteases”) such as iRhoms, Derlins, and other distantly related forms.” reads as if there were 2 main groups (proteolytically active and catalytically inactive) and that the catalytically inactive further subdivide into to two subgroups (mentioning even three subgroups, iRhoms, Derlins and other distantly related forms). This is not a clear description how this protein family is organized and which groups/subgroups are distinguished. I haven’t read the other 4 cited papers, just the one by Lemberg and Freeman, thus I cannot suggest how to rephrase this sentence to be consistent with the literature. In the end, it should be clearly stated which and how much major subgroups are distinguished, and which of these are further distinguished in minor subgroups, and which of the classifications is based on which publication. The next sections are at least not in-line with the Lemberg and Freeman classifications, as they state “The active category…” implying no subgroups and “The inactive categories…” implying multiple subgroups.

 => The citation of previous literature in the Introduction section is still not adequate. While the citations in the section about “active rhomboids” is appropriate putting citations to the listed functions, in the section about “inactive rhomboids” all citations are just combined at two occasions, although many more different functions of rhomboids are listed than in the “active” section. It should be the authors’ and not the readers’ task to put citations at appropriate statements. As it is, there are multiple citations for very general statements, while all the detailed information is not supported by citations.

 => “…At1g741301, also exists as three splice variants…”: What does “also” mean here? Where is the reference to other rhomboids with 3 splice variants?

 => “…changing from a seven predicted to six transmembrane structure”: I don’t understand that phrase, this does not make any sense.

 => “Functionality differences…”: What does this mean? There are many more awkward phrasings throughout the manuscript, that I will not list here. I highly recommend the authors to go through the manuscript again and improve the phrasing. There are examples in almost every second sentence… (e.g. “A limited selection of alternatively spliced variants were…”, “In many cases, alternative splicing events tend to influence one motif, but there were a number of cases where the impact may affect one or more motifs…”, etc. etc.)

 => “…we conducted a comparative analysis of entries available in the genetic databases…”: Did you really look in “genetic” databases (and if, I would be very curious to know which exactly) or genomic databases?

 => Methods section: “For instance, a frequent occurrence is an alternative splicing event which occurs in a particular motif that would likely impact the adjacent linker region.” Does this sentence provide any useful information? Is it related to any “method”? Does it implicate that “particular motifs” as part of alternative splicing events are only located in domains (e.g. TMs) and not in linker regions? Does it implicate that these motifs never impact the motifs themselves or the corresponding domains but only the linker regions? Wouldn’t such events rather impact spatially close regions (e.g. tertiary structure) instead of regions connected by sequence succession? I highly recommended in my first review to revise the Methods section with respect to removing unnecessary information not related to any Method, and instead provide a description what was actually done with which software (this includes mentioning all the software versions! BLAST v.1.0 is very different from BLAST v.2.28, for example). The Methods section needs further shortening (remove all what is not a method) and considerable rephrasing (to make exact statements; e.g. what is a “Linear protein alignments…”? Are there circular or other alignments? What are “Structural predictions…”, “Three-dimensional predictions…”?).

 => Shouldn’t it be “selected” instead of “select” in the title and at many other occasions in the manuscript, e.g. “Select proteins were synthesized…”?

 => I only looked at Figure S1 in detail, and because this is already that strange and misleading I stopped and did not look at the other supplementary figures. From its title “Examples of alternative splicing and their potential impacts on the structure of the amino terminal region” the reader would suspect to see a structure of the N-terminal region, and structures where this region is changed because of alternative splicing events. At first glance, Figure S1 shows 13 structures in “A” and an alignment of 13 sequences in “B”. However, the structure denotes TMD3-6, which are not part of the N-terminal region and not part of the red-boxed region of the alignment, which shows the differences due to alternative splicing. Where is the N-terminal region in the structure? According to the figure legend, “Key residues of the catalytic site are noted by colorized letters that match the colorized regions of the generated 3-D models”, but in the 3D-models all secondary structural elements are coloured, not the key residues. There is no way to identify these “key residues” anywhere in the structure. According to the alignment shown in “B” there are three splice events: i) there could be a short elongation at the N-terminus, ii) there is a differentially included region in the middle, and iii) almost the entire N-terminal region can be missing. But where do I see this in the structures? Sequences x1, x2, and x6 should have identical N-terminal sequences, but the corresponding structures in “A” are different. Or do numbers in “A” not correspond to numbers in “B” as stated in the figure legend? Same for sequences x3, x4, and x5, which are identical but do show different structures. Why do have the sequences x10, x11, and x12, which almost completely miss the N-terminus, structures at all? The sequences for the N-termini of all 13 variants are identical (except for including/excluding certain regions), why are the structures for these N-termini different?

I confirm that I have read this submission and believe that I have an appropriate level of expertise to state that I do not consider it to be of an acceptable scientific standard, for reasons outlined above.

# Peer review: Reviewer response for version 1

**DOI:** 10.5256/f1000research.14530.r31937

**Published:** 2018-04-03

**Authors:** Yuriy L Orlov

**Affiliations:** 1 Institute of Cytology and Genetics (SB RAS), Siberian Branch of the Russian Academy of Sciences (SB RAS), Novosibirsk, Russian Federation; 2 Novosibirsk State University, Novosibirsk, Russian Federation; 3 Sechenov University, Moscow, Russian Federation

The manuscript presents original materials on alternative splicing of rhomboid proteins.

 The authors discuss potential impacts on the rhomboid protein structure.

 The paper has good illustration and rich Supplementary materials. A lot of work done. Thus, it is technically sound.

 But the presentation has to be improved. A lot of unnecessary literature citations (such as 2-31).

 Thus, the work does not have clear literature citations.

 Choice of model objects is not clear.

 Alternative splicing prediction depends on computer tools. If we compare splice variants of the same protein (homolog) from different species, the parameters of prediction should be comparable. Some model organisms, such as human and mouse, have much more experiments and works, and just better gene annotation than for plant genomes. Thus, we can't directly compare number of splice variants.

 Thus, I consider the statistical analysis and its interpretation only partly appropriate.

 And not fully reproducible - to answer the question ' Are all the source data underlying the results available to ensure full reproducibility?'

 'Periodic comparison' or 'multi-year analysis' of the information on the proteins family databases should be explained. What is the time for database releases? What kind of trend in functional potential could annotation of these proteins prove?

 I recommend remove that part of the paper.

 The study is based on the work by the authors in 2012 on the Arabidopsis thaliana homologs containing alternative splice variants. The current article should show novel materials and conclusion. Comparison by the available information updates on these homologs from model organism databases is a weak idea. If you wait longer, more model species will be functionally annotated, and definitely some new splicing variants found. So, it could be either predictive model, or just description of all data available now on rhomboid proteins.

 'Multi-year analysis' is a redundant term - I recommend remove such part of text, or reformulate it.

 Overall, the article presents novel ideas on the cross-species comparison of alternative splicing. This science area is growing and we might expect new evidence for alternative splicing from genome sequencing projects.

 The conclusions drawn by the authors are adequately supported.

 Thus, this work is of interest for *F1000Research *readers.

 Thus, I recommend **'Approved with Reservations**' status.

I confirm that I have read this submission and believe that I have an appropriate level of expertise to confirm that it is of an acceptable scientific standard, however I have significant reservations, as outlined above.

# Response

**Published:** 2018-04-04

**Authors:** Kenton Ko

**Affiliations:** Queen's University, Kingston, Canada

Thank you for taking the time and effort to review our submission.  This is very much appreciated!

The feedback provided from the different reports is valuable to us and will guide our revisions for the next version of this contribution. 

We will be in touch soon with the next version.

# Response

**Published:** 2018-05-22

**Authors:** Kenton Ko

**Affiliations:** Queen's University, Kingston, Canada

Dear Dr. Orlov,

 Thank you for agreeing to review version 1 of our submission and helping us with your assessment.  This is much appreciated! 

 We now have version 2 available for your further assessment.  Based on the feedback, Version 2 was revised substantially to address the aspects raised and to enhance clarity in the areas of writing style and additional details. 

 As per your suggestions, improvements were made to the presentation by removing references that may not be necessary to the readers.  Removal of these extra references allowed us to revise the Abstract, Introduction, and Methodology sections to enhance clarity with respect to the study’s focus or purpose.   The title was also modified slightly to reflect these revisions.

 The choice of model organisms used was explained better by revising the style of writing/presentation and by characterizing the work as a comparative analysis or survey of available entries, as opposed to a direct comparison of splice variant numbers between species and its interpretation in this way.  We hope that we have interpreted this suggestion correctly.  This aspect, we believe, was also raised by Reviewer 1, Dr. Kollmar.

 We have revised the Methods section substantially to clarify the approaches used and to provide more needed details concerning the analysis of available database entries and the functionality assays using recombinant proteins.  Revisions were also made to the Source Datasets and the Supplementary Tables to enhance their presentation, to provide more descriptive titles and related aspects.  We hope that there is now sufficient methodological details for others to follow.

 The multi-year facet of the database work was removed to avoid confusion and to enhance clarity. This removal allowed us to streamline the database analysis protocols and provide more details of the tools used.  The Results and Discussion sections were modified similarly to reflect these changes.  The removal of the multi-year aspect in combination with the above revisions should help the readers interpret the work more clearly. 

 Sincerely,

 Kenton Ko and Josh Powles

# Peer review: Reviewer response for version 1

**DOI:** 10.5256/f1000research.14530.r31395

**Published:** 2018-03-12

**Authors:** Lu-Yuan Li

**Affiliations:** 1 State Key Laboratory of Medicinal Chemical Biology, College of Pharmacy ,Tianjin Key Laboratory of Molecular Drug Research, Nankai University, Tianjin, China

The rhomboids are a very large family of genes found across all kingdoms from bacteria to plants to animals. Based on what we know about the functions of these genes, which unfortunately is not very much at the moment, they often play critical roles in life wherever they are involved. Also interesting is that their functions are highly diversified, as mentioned in this paper. For instance, the human rhomboid family 1 gene, RHBDF1, was found to be expressed at significantly higher levels in breast cancer tissues than in normal breast tissues, with functions as different as promotion of G-protein coupled receptor mediated transactivation of epidermal growth factor receptor (reference 1), or protection of hypoxia-inducible factor 1-alpha from degradation under hypoxic conditions (reference 2); both functions are consistent with facilitating tumor growth, however. RHBDF1 belongs to the group of so-called inactive rhomboids (iRoms) because of an apparent lack of protease activities associated with otherwise enzymatically active rhomboids. This group of rhomboids, having lost their abilities to catalyze proteolysis reactions due to mutations, seem to have destined in evolution to perform other functions, such as those of chaperones, with their abilities to bind to a variety of proteins conserved and utilized in assisting protein folding, transportation, and degradation.

 The paper by Powles and Ko is interesting as it addresses a fundamental cause of the massive variations of the functions of the rhomboid gene products. The authors carried out an extensive data mining operation to reveal complex differential splicing patterns of the rhomboid genes. Their findings indicate that differential splicing is common and substantial within one gene as well as throughout the gene family. Apart from the multiple transmembrane domains, the rhomboid proteins often possess a large N-terminal domain before the first transmembrane domain and a sizable loop between the first and second ones. The N-terminal and the loop are likely located on opposite side of the endoplasmic reticulum, Golgi, or plasma membrane of the cell, indicating that they have opportunities to interact with different biomolecules. In addition, in the case of RHBDF1 there are a number of amino acid residues within the protein molecule that could be subjected to post-translational modifications such as phosphorylation and glycosylation. It would therefore seem highly likely that frequent and extensive splicing of the gene transcripts exerts considerable impact on the protein structures and thus their functions. Changes in the gene transcripts could also bring about alterations in terms of modulations by micro RNA and other non-translational means. The effort by the authors is very useful to put together a data base of immense and complex differential splicing patterns of nearly the entire rhomboid gene family. The findings should be beneficial to researchers in this field, even though many of the conclusions are speculative, as pointed out by the authors, because of the lack of our knowledge on the structures and functions of most of the individual products of this enormous gene family.

I confirm that I have read this submission and believe that I have an appropriate level of expertise to confirm that it is of an acceptable scientific standard.

## References

[1] : Human rhomboid family-1 gene RHBDF1 participates in GPCR-mediated transactivation of EGFR growth signals in head and neck squamous cancer cells. *FASEB J*.2009;23(2) : 10.1096/fj.08-112771 425-32 10.1096/fj.08-112771 18832597PMC2638965

[2] : Human rhomboid family-1 suppresses oxygen-independent degradation of hypoxia-inducible factor-1α in breast cancer. *Cancer Res*.2014;74(10) : 10.1158/0008-5472.CAN-13-1027 2719-30 10.1158/0008-5472.CAN-13-1027 24648344

# Response

**Published:** 2018-04-04

**Authors:** Kenton Ko

**Affiliations:** Queen's University, Kingston, Canada

Thank you for taking the time and effort to review our submission.  This is very much appreciated!

 The feedback provided from the different reports is valuable to us and will guide our revisions for the next version of this contribution. 

 We will be in touch soon with the next version.

# Response

**Published:** 2018-05-22

**Authors:** Kenton Ko

**Affiliations:** Queen's University, Kingston, Canada

Dear Dr. Li,

Thank you for agreeing to review version 1 of our submission and for your assessment.  This is much appreciated!  We now have version 2 available for viewing.  Based on the feedback, Version 2 was revised substantially to address the aspects raised and to enhance clarity for the readers in the areas of writing style and additional details.  The nature of the work reported was not altered by the revisions.

The presentation of the study’s purpose and approach used was revised for clarity.  This was achieved first by removing most of the references listed from 2 to 26 so that the references cited were more obviously directed at the study, as opposed to providing general background of the wider field.  Accompanying revisions were then made to the Abstract and Introduction.

The Methods section was revised substantially to clarify the approaches used and to provide more needed details concerning the comparative analysis of database entries and the functionality assays using recombinant proteins.  The title was also modified slightly to align with these revisions.  The Results and Discussion sections were modified similarly to reflect the changes.  The multi-year facet of the database analysis was deemed unnecessary and removed. Revisions were also made to the Source Datasets and the Supplementary Tables to enhance their presentation.  Collectively, these revisions should help the readers interpret the work more clearly with the additional details and streamlining of the protocol for the database work.

Sincerely,

Kenton Ko and Josh Powles

# Peer review: Reviewer response for version 1

**DOI:** 10.5256/f1000research.14530.r30490

**Published:** 2018-02-22

**Authors:** Martin Kollmar

**Affiliations:** 1 Group Systems Biology of Motor Proteins, Department of NMR‐Based Structural Biology, Max‐Planck‐Institute for Biophysical Chemistry, Göttingen, Germany

Joshua Powles and Kenton Ko conducted a study on rhomboid serine proteases with respect to their alternative splice variants. The study is based on a discovery of the same researchers in 2012 that two of the Arabidopsis thaliana homologs contain alternative splice variants. The manuscript displays several conceptual problems that would demand a major revision if touched. Therefore I suggest the authors to completely revise all in silico analyses and completely re-write the paper. The problems are as follows:

 => The authors provide much unneeded information for certain aspects (which would just be a style problem) but, most importantly, miss a lot of essential information to recapitulate and redo their analyses. Example: The authors state in the Methods section: “All variant RNA sequences were assumed to result in the generation of functional proteins, including extensively truncated products.” This, however, is not a method. A method would be that the authors write, that they used tool-x or tool-y to translate the RNA sequences, which they subsequently analysed by tool-z. Instead, the authors continue: “The resulting predictions did not indicate that this assumption would not hold true.” What does “resulting” mean here? Which tool was used? A translation of an RNA is usually not termed “prediction”. Which tool was used for the verification? Apart from these style problems, the authors did not provide any evidence that the translations “do” result in functional proteins. It still is just their assumption. In terms of missing information to recapitulate/redo the analyses, the authors do not provide any information on: A) how the set of starting proteins were assembled (search by name? Using BLAST?), B) how the entries were assessed for alternative splicing (on RNA? On protein level? By alignment?), C) the authors claim that their analyses required comparisons between genomic sequences but do not provide any genomic sequence in the main text or supplements, D) the authors claim “alignments with current models of rhomboid proteins” without providing the tool used for doing these alignments nor providing a list or reference for the “current models of rhomboid proteins”, E) the authors state that they used Ni-NTA chromatography for protein purification without providing any buffer. While column washing-buffers likely do not have any effect, the elution buffer has. Was the elution done in a gradient? By a step-wise process? Just by eluting with 500 mM Imidazole? Which pH? Was the purified protein dialysed afterwards? This is essential information, because the proteins were subsequently used in other assays, and these might be compromised by pH or imidazole concentration. F) the authors do not provide any information on ampicillin-concentration used for selection, nor any cell lysis procedure. G) was the purified protein always used directly? Frozen and reused later? How long is the protein stable in which buffer (pH? Imidazole?)? H) I do not understand this sentence: “If used, the rabbit polyclonal anti-rhomboid protein (At1g74130) antibodies were established by our lab and validated as reported in Powles et al. 51 .” Did the authors use this antibody for the western blots? Why did they “validate” the antibody again? I) which media were used for yeast cell growth? Just writing “Cells were grown in glucose-supplemented media” does not make the experiment reproducible. Which glucose concentration? What is a “complete medium”? J) The authors experimentally analyse three variants for the At1g74130 gene, termed L, M, and S, but it is not described anywhere to which splicing events these transcripts belong. Every study that I know that deals with alternative splicing contains a gene structure scheme and shows which exons would be present in which transcript. This is just an incomplete list of examples, where absolutely essential information is missing. The authors would need to check every sentence in the Methods section to see, whether it is really describing a method, and they need to check where and which information is missing.

 => I have looked at the provided accession numbers for the genes/proteins. The numbers for human and mouse all represent “predicted transcripts”, thus these could all be wrong. I recommend the authors to read a few papers about gene prediction software and pipelines, they will notice that on average every prediction in e.g. human, Drosophila, C.elegans contains 1 wrong exon (e.g. the paper from the eGASP comparison of gene prediction software on these 3 species would be a good starter). Prediction of alternative splice variants is even more error-prone. Thus, the authors should only use transcripts with cDNA evidence in their study (and keep in mind, that cDNA/EST data also sometimes contains errors from e.g. missplicing). Discussing just gene predictions is complete artificial and fiction. The authors could, for example, download a few RNA-seq datasets from the ENCODE and modENCODE data and do the RNA-seq mapping themselves to validate the gene predictions. If the authors cannot do this, they should only analyse and discuss transcript variants for which they find cDNA/EST evidence (and of course the accession numberss for these cDNA sequences need to be given). If the authors did a RNA-seq mapping themselves they could also provide some measure for the likeliness that a suggested variant is a true variant or the result of missplicing, transcription errors, etc. E.g. if thousand of reads are found for a certain gene, is it likely that a variant is a true variant and functional if only supported by 1-2 reads? Or are these 1-2 reads rather representing missplicing and other errors?

 => The authors claimed several times that they re-did the analysis every 6 months since 2012. Wouldn’t it be a much better way to just look first whether updates on these species were made available at all before spending time in redoing an analysis? I know that these updates only happen occasionally, and not even every second year. Also, the techniques completely changed since about 2010. At least I am not aware of any major study providing new EST/cDNA datasets for the species studied here. All the new data since about 2008 is generated as RNA-seq data. Thus, which further cDNA evidence did the authors expect for the transcripts since 2012 so that they decided to redo the analysis every 6 month? I highly recommend the authors to read all the README-files for the various gene prediction datasets that GenBank and the other databases provide. E.g. Ensemble decided in 2012 (if I remember correctly) to not use any cDNA data anymore for validating their gene predictions. Thus, many exons with cDNA data available (e.g. from isolating and sequencing single genes by research groups) are not present in Ensemble’s gene predictions anymore, if these exons are not predicted by gene prediction tools. Similarly, there are many predicted exons for which no evidence (cDNA/RNA-seq) is available. Please check the GETx-project: There you can see how many RNA-seq reads in each tissue are found for each transcript. You will find out, that for many of the “alternative” transcripts, there is not a single read. The presented discussion of the variants (figures 3 to 6) supports my assumption that most predictions are just wrong. Could the authors provide any reference that it would be possible for such a seven-transmembrane-protein (the rhomboid proteases) to result in a functional protein if e.g. 1 transmembrane region in the middle would be missing due to alternative splicing? If 1 transmembrane region would be missing (this is suggested by several variants the authors discuss) this would turn the direction of the rest of the transmembrane helices and regions: what was inside before would be outside, and what was outside would be inside. To all what I know from membrane protein structures, the transmembrane helices stick together forming a dedicated tertiary structure. Could the authors provide a reference that transcripts with early terminations would ever result in stable and functional proteins, if e.g. only some of the 7 transmembrane helices are present anymore? The C.elegans ROM-4 was stated to contain a variant with just the first TM-helix present. Do the authors really think that this would result in a functional protein? How do the authors exclude that such misspliced/unstable variants would not result in NMD?

 => The authors claim several times throughout the entire manuscript that they analysed selected model species without giving any information how these were selected. E.g. wikipedia contains a list of about 100 model organisms. Which was the rationale to just look at the 6 in the manuscript? Why not more plants? In this respect, there are many statements of the authors that are just wrong, such as “Currently, of the sequenced model genomes available,”. Of the 100 model organisms at wikipedia (and there are likely more model organisms if other researchers were asked) at least for 80 of them complete genomes are available. Altogether, there are about 5000 eukaryotes with genome assemblies available, of which at least 4000 are complete (e.g. check www.diark.org). In this respect, all the speculations and discussion about which organism contains the most homologs or the most variants are just speculations and should explicitly termed as such. E.g. Brassica species underwent another whole-genome-duplication after separation from the Arabidopsis, and will thus contain many more than the 22 Arabidopsis homologs. Fish also underwent another 1 or 2 (depending on lineage) whole-genome-duplication, therefore will also contain more homologs.

 => The authors state several times that, although not observed, they expect more alternative variants for the Arabidopsis homologs, because many more variants were identified for human. What is the basis for this expectation? Is there any reference that demonstrates, that Arabidopsis genes have as many alternative splice variants as mammalian genes, on average? All what I am aware of just contradicts this expectation. Arabidopsis genes have fewer exons (thus less possibilities for alternative splicing) and less alternative splice events. Why do the authors not expect more alternative variants for Drosophila or C.elegans, based on their rationale? Shouldn’t yeast have at least some variants (doesn’t have any yet)? Of course, yeast doesn’t, but this should make the authors aware of the problems in their argumentation.

 => Although the manuscript deals with alternative splice variants, I did not find any reference on the ample literature on this subject. Not even references to a few reviews. Similarly, there is not even mentioning of the accepted types of alternative splicing, e.g. differentially included exons, alternative 5’/3’ splice sites, mutually exclusive splicing, etc. Categorizing the variants detected with these categories would be much more informative than categorizing by region. Based on the descriptions in the supplementary tables, many variants are highly likely just the result from sequencing errors leading to frame-shifts or alternative amino acids. There is only a single alternative splice form that would lead to alternative amino acids for a certain regions, which is mutually exclusive splicing, but this information I did not find anywhere. This could easily be confirmed.

 => I did not see any gene structure in the manuscript or supplements. The usual procedure in the field is to provide a gene structure drawing of each gene and mark the splicing events on these structures. This is common practice since >30 years. The authors do not show any protein sequence, nor any cDNA sequence, nor any alignment. I cannot see any use of the provided tables in the supplements for other researchers. Terms such as “Original UTR missing, new UTR generated from coding region” (table S3) are not useful. How can a UTR be generated from a coding region? Does this mean that this is just an alternative translation start site? What does “Default 5’UTR missing, extended downstream from isoform 1” mean? When I think of a gene structure with exons and introns, which splice event would represent this prosaic description? I will not provide more examples here, but by browsing through the supplementary tables I did not find a single useful description. Thus, the authors should provide a gene structure scheme for each gene and mark the events for each gene accordingly. Such schemes would represent exact descriptions of the splicing events. All prosaic descriptions need to be removed.

 => The authors state in the Methods section: “This was necessary to acquire sequences that resulted from alternative splicing only, as opposed to derivations from other routes.” Which other routes would lead to variants, if not alternative splicing?

 => The authors state in the Methods section: “A prime example of such a situation occurred with the human database entries, where the newest version contained more verifications than the previous versions.” How did the authors determine, whether the newest version contains “more verifications”? Is there any reference that GenBank obtained more full-length cDNA data in 2016 and that these data were used in the gene predictions?

 => The authors confirm by experiments the alternative splicing of two Arabidopsis genes, but exactly these variants have already been published by the same group some years ago. Thus the authors do not provide any further and new evidence that any of the predicted variants of the other genes is present and functional. The authors would not even need to do experiments to show functionality. If they only analyse those transcripts for which cDNA/EST data are available, these cDNA/EST data are already the evidence. Further evidence could be obtained if the same transcripts were found for closely related species.

 => The Introduction is extremely unbalanced. E.g. for this very general statement “Rhomboids are also part of an even larger group of proteins involved in regulated intramembrane proteolysis 2–31.” the authors provide 30! references, but the authors miss to provide a useful description of the protein superfamily in general. As far as I understand, these proteins are present in bacteria, archaea and eukaryotes. It would be essential information how the various subgroups are phylogenetically separated. Without such information, there is no possibility to understand the variants. For example, which of the Arabidopsis homologs are the result of the many plant whole-genome duplications? Do such duplicates have identical alternative splicing? Similar for human and mouse: Which homologs are related to which Drosophila and C.elegans homologs, do the orthologs in human/mouse (derived by the two whole-genome duplications at the origin of the vertebrates) have identical splice variants?

I confirm that I have read this submission and believe that I have an appropriate level of expertise to state that I do not consider it to be of an acceptable scientific standard, for reasons outlined above.

# Response

**Published:** 2018-05-22

**Authors:** Kenton Ko

**Affiliations:** Queen's University, Kingston, Canada

Dear Dr. Kollmar,

Thank you for agreeing to review version 1 of our submission and helping us with your assessment.  This is much appreciated! 

We now have version 2 available for your further assessment.  Based on the feedback, Version 2 was revised substantially to address the aspects raised and to enhance clarity for the readers in the areas of writing style and additional details. 

As per your suggestions, improvements were made to the presentation by removing references that may not be necessary to the readers.  Removal of these extra references allowed us to revise the Abstract, Introduction, and Methodology sections to enhance clarity with respect to the study’s focus or purpose.   The title was also modified slightly to reflect these revisions.

We have revised the Methods section substantially to clarify the approaches used and to provide more needed details concerning the analysis of available database entries and the functionality assays using recombinant proteins.  Revisions were also made to the Source Datasets and the Supplementary Tables to enhance their presentation, to provide more descriptive titles and related aspects.  We hope that there is now sufficient methodological details for others to follow.

The style of writing and presentation in the Methods was revised substantially.  We have removed unneeded information and added needed details/information on the tools used, tools that reflect what was being done in the comparative analysis of available entries – namely that we compiled all available entries and assessed their status as splice variants using RNA and protein data.  We have added information in the text and in the tables to clarify what the compiled entries represent, e.g., entries or accession numbers listed could represent predicted entries but they are listed and used as reference points after assessment with RNA and protein data.

We have added more information (or information deemed missing) concerning the recombinant protein work and revised the information to enhance clarity.  For splicing information pertaining to the At1g74130 (L, M, S) and At1g25290 (L, S) protein variants used later in the study, we have added citations and direction to the Supplementary Material for protein information.

The multi-year facet of the database work was removed to avoid confusion and to enhance clarity. This removal allowed us to streamline the database analysis protocols and provide more details of the tools used.  The Results and Discussion sections were modified similarly to reflect these changes.  The removal of the multi-year aspect in combination with the above revisions should help the readers interpret the work more clearly. 

Concerning the aspect of protein variants with substantial changes being potentially functional, we have provided citations as possible indications that major changes could still give rise to rhomboid proteins with functionality, such as early termination resulting in proteins with one missing transmembrane region.

The choice of model organisms used was explained better by revising the style of writing/presentation and by characterizing the work as a comparative analysis or survey of available entries, as opposed to a direct comparison of splice variant numbers between species and its interpretation in this way.  We hope that we have interpreted this suggestion correctly.  This aspect, we believe, was also raised by Reviewer 3, Dr. Orlov.

We did not include references dealing with alternative splicing because the study was not intended to be at this level, but working at the level of surveying and comparing available entries and what these entries may reflect at the protein level.  As described above, substantial revisions were focused on clarifying the intent of the work to avoid this impression.  This reasoning applies to why gene structures and splicing events were not provided.  All of the supplementary tables were revised to enhance the writing style of the descriptions and render them more useful.  We hope that the revisions provided clarity on these issues.

The latter comments concerning passages in the Methods section were addressed by the revisions outlined above.  This would also be the case for the comments pertaining to the Introduction – addressed by the revisions outlined above.

Sincerely,

Kenton Ko and Josh Powles
